
JOURNAL INFORMATION
==============================
NLM Title Abbreviation: Insights Imaging
Journal ID: Insights Imaging
NLM Journal ID: 101532453

Insights into Imaging

EISSN: 1869-4101
Publisher: Springer

ARTICLE INFORMATION
==============================
PMCID: PMC9149671
PMID: 35635582
DOI: 10.1186/s13244-022-01225-4
Article ID: 1225
Article version: 2
Subjects: Meeting Abstracts

ESGAR 2022 Book of Abstracts

Electronic publication date: 2022 May 30
Publication date: 2022 May
Volume: 13
Issue: Suppl 2
Issue sponsor: Publication costs were funded by the European Society of Gastrointestinal and Abdominal Radiology.
Electronic Location ID: 92
Copyright: © The Author(s) 2022
License: Open Access This article is licensed under a Creative Commons Attribution 4.0 International License, which permits use, sharing, adaptation, distribution and reproduction in any medium or format, as long as you give appropriate credit to the original author(s) and the source, provide a link to the Creative Commons licence, and indicate if changes were made. The images or other third party material in this article are included in the article's Creative Commons licence, unless indicated otherwise in a credit line to the material. If material is not included in the article's Creative Commons licence and your intended use is not permitted by statutory regulation or exceeds the permitted use, you will need to obtain permission directly from the copyright holder. To view a copy of this licence, visithttp://creativecommons.org/licenses/by/4.0/.
License URL: https://creativecommons.org/licenses/by/4.0/

Conference name: 33rd Annual Meeting and Postgraduate Course ESGAR 2022
Conference location: Virtual
Conference date: 31 May-3 June 2022
issue-copyright-statement: © The Author(s) 2022

==============================

European Society ESGAR Gastrointestinal and Abdominal Radiology

### Insights into Imaging

Education and strategies in European radiology

ESGAR 2022 Book of Abstracts / Volume 13 / Supplement 2 / May 2022

ESGAR 2022 / May 31 – June 3 / Lisbon, Portugal
33rd Annual Meeting and Postgraduate Course

ESR European Society of Radiology

European Society
ESGAR
Gastrointestinal and Abdominal Radiology

### ESGAR

### Annual Meeting and Postgraduate Course

### 2022

### May 31 - June 3

LISBON
PORTUGAL

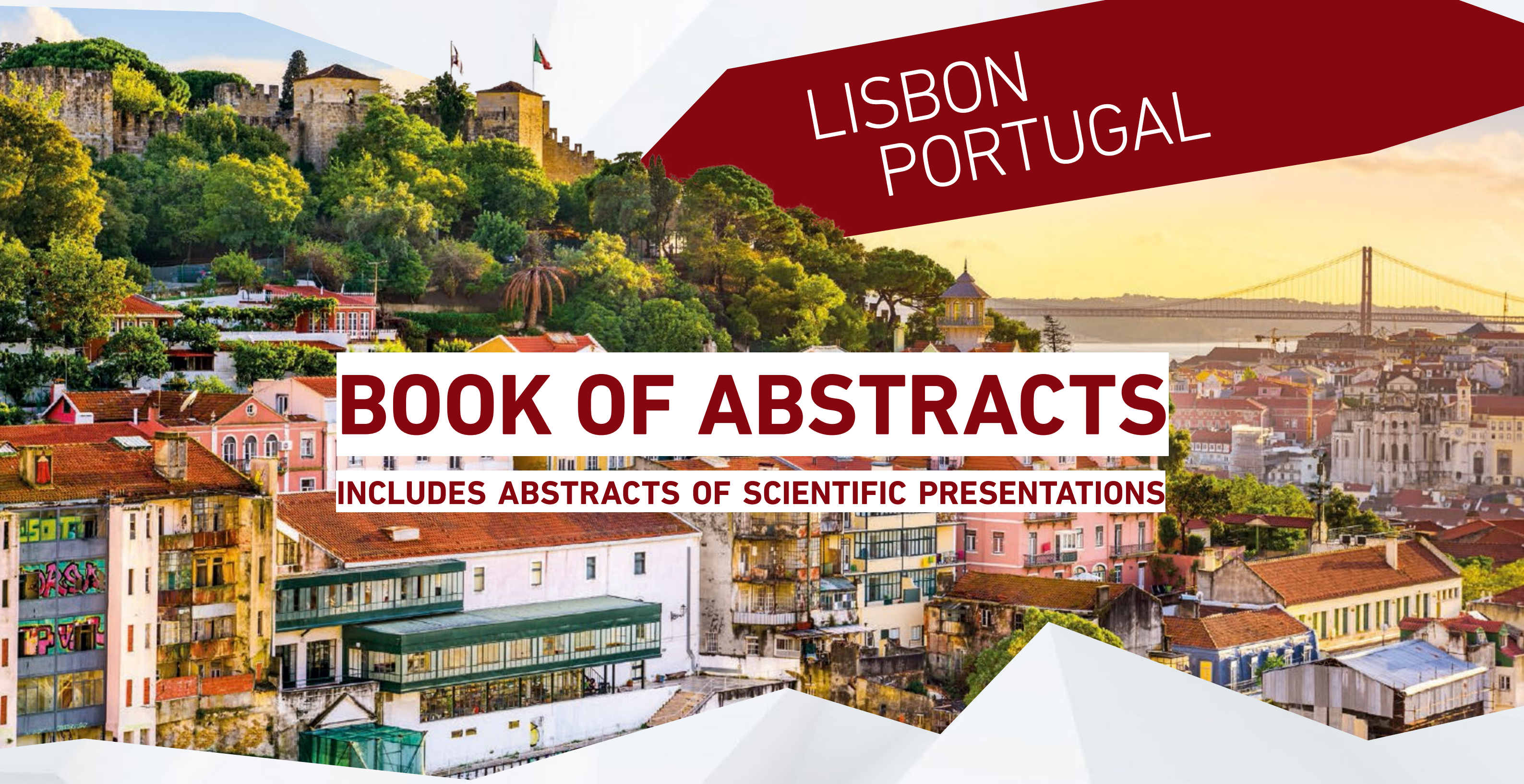

### BOOK OF ABSTRACTS

**INCLUDES ABSTRACTS OF SCIENTIFIC PRESENTATIONS**

### *IMPORTANT ADDRESSES*

**ORGANISING SECRETARIAT**
**Central ESGAR Office**
Esslinggasse 2/3
AT – 1010 Vienna
Phone: +43 1 535 89 27
E-Mail: office@esgar.org www.esgar.org

### CME CREDITS

The "ESGAR European Society of Gastrointestinal and Abdominal Radiology" (or) "ESGAR 2022 – 33rd ANNUAL MEETING AND POSTGRADUATE COURSE" is accredited by the European Accreditation Council for Continuing Medical Education (EACCME) to provide the following CME activity for medical specialists. The EACCME is an institution of the European Union of Medical Specialists (UEMS), www.uems.net.

The 33rd Annual Meeting and Postgraduate Course ESGAR 2022, Lisbon, Portugal, 31/05/2022-03/06/2022 has been accredited by the European Accreditation Council for Continuing Medical Education (EACCME®) with **26** European CME credits (ECMEC®s). Each medical specialist should claim only those hours of credit that he/she actually spent in the educational activity.

### CONTRIBUTING SOCIETIES

European Society of Urogenital Radiology

SAR Society of Abdominal Radiology™

### SPONSORS

ESGAR wishes to gratefully acknowledge the support of its Corporate Members:

BAYER

BRACCO LIFE FROM INSIDE

GE

Guerbet

The Final Programme of ESGAR 2022 is available on the ESGAR Website www.esgar.org

Date of publishing: 30 May 2022

Insights Imaging (2022) 13 (Suppl 2):XX
https://doi.org/10.1186/s13244-022-01225-4
Published: 01 March 2022

### *TABLE OF CONTENTS*

| | | |
|---|---|---|
| Scientific Sessions, Wednesday, June 1 | (SS 1.1 – SS 1.9) | 8-10 |
| Scientific Sessions, Wednesday, June 1 | (SS 2.1 – SS 2.10) | 10-13 |
| Scientific Sessions, Wednesday, June 1 | (SS 3.1 – SS 3.10) | 13-15 |
| Scientific Sessions, Wednesday, June 1 | (SS 4.1 – SS 4.10) | 16-18 |
| Scientific Sessions, Thursday, June 2 | (SS 5.2 – SS 5.10) | 19-21 |
| Scientific Sessions, Thursday, June 2 | (SS 6.1 – SS 6.9) | 21-23 |
| Scientific Sessions, Thursday, June 2 | (SS 7.1 – SS 7.10) | 24-26 |
| Scientific Sessions, Thursday, June 2 | (SS 8.2 – SS 8.10) | 26-28 |
| Scientific Sessions, Thursday, June 2 | (SS 9.1 – SS 9.10) | 29-31 |
| Scientific Sessions, Friday, June 3 | (SS 10.1 – SS 10.10) | 32-34 |
| Scientific Sessions, Friday, June 3 | (SS 11.1 – SS 11.10) | 35-38 |
| Scientific Sessions, Friday, June 3 | (SS 12.1 – SS 12.9) | 38-40 |
| Scientific Sessions, On Demand | (SSD 1.1 – SSD 1.4) | 41-41 |

AUTHORS’ INDEX 43-47

### ESGAR EXECUTIVE COMMITTEE

PRESIDENT
*A. Laghi, Rome/IT*

PRESIDENT-ELECT
*M. Zins, Paris/FR*

VICE PRESIDENT
*J. Stoker, Amsterdam/NL*

SECRETARY/TREASURER
*V. Vilgrain, Clichy/FR*

PROGRAMME COMMITTEE CHAIR
*J. Stoker, Amsterdam/NL*

EDUCATION COMMITTEE CHAIR
*C.J. Zech, Basel/CH*

MEMBERSHIP COMMITTEE CHAIR
*G. Brancatelli, Palermo/IT*

RESEARCH COMMITTEE CHAIR
*S.A. Taylor, London/UK*

WORKSHOP COMMITTEE CHAIR
*M.A. Bali, Brussels/BE*

MEETING PRESIDENT 2022
*L. Curvo-Semedo, Coimbra/PT*

MEETING PRESIDENT 2023
*A. Torregrosa Andrés, Valencia/ES*

MEETING PRESIDENT 2024
*L. Blomqvist, Stockholm/SE*

MEMBERS AT LARGE
*S. Gourtsoyianni, Athens/GR*
*M. Karcaaltincaba, Ankara/TR*
*N. Kartalis, Stockholm/SE*
*M. Radzina, Riga/LV*
*D. Tolan, Leeds/UK*

ESGAR EXECUTIVE DIRECTOR
*B. Lindlbauer, Vienna/AT*

### ESGAR 2022 MEETING PRESIDENT

*Luís Curvo-Semedo*
*Faculty of Medicine, University of Coimbra*
*Department of Medical Imaging,*
*Coimbra University Hospitals*
*CIBIT - Coimbra Institute for Biomedical*
*Imaging and Translational Research*
*Coimbra, Portugal*

### ESGAR 2022 PROGRAMME COMMITTEE

CHAIRMAN
*J. Stoker, Amsterdam/NL*

MEMBERS
*M.A. Bali, Brussels/BE*
*L. Blomqvist, Stockholm/SE*
*G. Brancatelli, Palermo/IT*
*L. Curvo-Semedo, Coimbra/PT*
*S. Gourtsoyianni, Athens/GR*
*M. Karcaaltincaba, Ankara/TR*
*N. Kartalis, Stockholm/SE*
*A. Laghi, Rome/IT*
*M. Radzina, Riga/LV*
*S.A. Taylor, London/UK*
*D. Tolan, Leeds/UK*
*A. Torregrosa Andrés, Valencia/ES*
*V. Vilgrain, Clichy/FR*
*C.J. Zech, Basel/CH*
*M. Zins, Paris/FR*

### ESGAR 2022 LOCAL ORGANISING COMMITTEE

*J. Brito, Faro/PT*
*F. Caseiro Alves, Coimbra/PT*
*L. Costa Andrade, Lisbon/PT*
*M. França, Porto/PT*
*L. Guimarães, Porto/PT*
*A.J. Madureira, Porto/PT*
*C. Matos, Lisbon/PT*
*M. Ramalho, Almada/PT*
*D. Ramos Andrade, Coimbra/PT*
*I. Santiago, Lisbon/PT*
*J. Traila Campos, Viana do Castelo/PT*
*J. Venâncio, Lisbon/PT*

*O. Akhan, Ankara/TR*
*T.V. Bartolotta, Palermo/IT*
*A. Ba-Ssalamah, Vienna/AT*
*N. Bastati-Huber, Vienna/AT*
*R.G.H. Beets-Tan, Amsterdam/NL*
*E. Biscaldi, Genoa/IT*
*A. Blachar, Tel Aviv/IL*
*L. Blomqvist, Stockholm/SE*
*I. Boulay Coletta, Paris/FR*
*G. Brancatelli, Palermo/IT*
*R. Cannella, Palermo/IT*
*F. Caseiro-Alves, Coimbra/PT*
*K. Cortis, Msida/MT*
*L. Crocetti, Pisa/IT*
*L. Curvo-Semedo, Coimbra/PT*
*G. D'Assignies, La Roche-sur-Yon/FR*
*M. Dioguardi Burgio, Villejuif/FR*
*A. Dohan, Paris/FR*
*R.F. Dondelinger, Liège/BE*
*M. D'Onofrio, Verona/IT*
*H. Fenlon, Dublin/IE*
*A. Furlan, Pittsburgh, PA/US*
*Y. Gandon, Rennes/FR*
*V. Goh, London/UK*
*M. Gollub, New York, NY/US*
*S. Gourtsoyianni, Athens/GR*
*J.A. Guthrie, Leeds/UK*
*T. Helmberger, Munich/DE*
*F. Iafrate, Rome/IT*
*D. Ippolito, Monza/IT*
*S. Jackson, Plymouth/UK*
*M. Karcaaltincaba, Ankara/TR*
*A. Laghi, Rome/IT*
*D. Lambregts, Amsterdam/NL*
*M. Laniado, Dresden/DE*
*J.M. Lee, Seoul/KR*
*O. Lucidarme, Paris/FR*
*M. Maas, Amsterdam/NL*
*A.J. Madureira, Porto/PT*
*M. Maher, Cork/IE*
*T. Mang, Vienna/AT*
*L. Martí-Bonmatí, Valencia/ES*
*C. Matos, Lisbon/PT*
*Y. Menu, Paris/FR*
*G. Morana, Treviso/IT*
*S. Mulé, Créteil/FR*
*E. Neri, Pisa/IT*
*P. Paolantonio, Rome/IT*
*N. Papanikolaou, Lisbon/PT*
*S. Pötter-Lang, Vienna/AT*
*R. Pozzi Mucelli, Verona/IT*
*P. Prassopoulos, Thessaloniki/GR*
*M. Ronot, Clichy/FR*
*W. Schima, Vienna/AT*
*S. Schmidt Kobbe, Lausanne/CH*
*A. Schreyer, Regensburg/DE*
*O. Seror, Bondy/FR*
*S. Skehan, Dublin/IE*
*M. Staunton, Cork/IE*
*S. Stojanovic, Novi Sad/RS*
*J. Stoker, Amsterdam/NL*
*C. Stroszczynski, Regensburg/DE*
*A. Taibbi, Palermo/IT*
*B. Taouli, New York, NY/US*
*J.P. Tasu, Poitiers/FR*
*S.A. Taylor, London/UK*
*D. Tolan, Leeds/UK*
*C. Triantopoulou, Athens/GR*
*V. Valek, Brno-Bohunice/CZ*
*F. Vernuccio, Padua/IT*
*V. Vilgrain, Clichy/FR*
*M.-P. Vullierme, Clichy/FR*
*M. Wagner, Paris/FR*
*G. Zamboni, Verona/IT*
*C.J. Zech, Basel/CH*
*M. Zins, Paris/FR*

European Society
ESGAR
Gastrointestinal and Abdominal Radiology

**09:00 - 10:30** **Auditorium 3 + 4**

*Scientific Session Live SS 1*
### Colorectal Cancer

### SS 1.1
**Improving the appropriateness of CT colonography referrals**
A.O. Yousif[1], M. Ismail[2], R. Vinayagam[3]; [1]*Leeds/UK*, [2]*Scarborough/UK*, [3]*Bradford/UK*

**Purpose:** Colonoscopy is the gold standard investigation for colorectal cancer but CT colonography (CTC) is used for those unable to tolerate. We aim to investigate the appropriateness of CTC referrals in a large district teaching hospital and how the CTC findings and patient management correlate.
**Material and Methods:** Retrospective audit and re-audit cycle looking at all the CTCs performed in patients aged 80 or above in a 6-month period (2019) was conducted. Consequently, a new referral pathway was implemented in which patients aged 85 or above were excluded from having a CTC unless exceptional circumstances. Indications for the CTC were also analysed as well as the average reporting time.
**Results:** 250 CTCs were performed in patients aged 80 or above. 18 (7%) had polyps. 13 were treated (72%) and the 5 (28%) not treated were 82 or older. The second cycle showed decreased number of CTCs, averaging 23/month (compared to 130/month) with only 6% of CTC in patients 85 or above. Higher percentage of positive patients were treated compared to before. Reduced reporting times were also achieved.
**Conclusion:** Although CTC allows other incidental assessment, it does not allow suspicious lesions to be biopsied and requires the patient to be mobile and competent. These factors among others are often not appreciated by the referrers; therefore, integrating an age limit with patient health status is suggested as part of the CTC referral pathway to reduce inappropriate CTC referrals. Performing sigmoidoscopy and a staging CT in the first line is also a suggestion if the patient cannot tolerate complete colonoscopy.

### SS 1.2
**Post-imaging colorectal cancers: evaluation of a 10-year experience of CT colonography in a high-volume centre**
D. Tolan[1], P. Melling[1], J. Taylor[1], N.E. Burr[2], P. Quirke[1], E. Morris[3]; [1]*Leeds/UK*, [2]*Wakefield/UK*, [3]*Oxford/UK*

**Purpose:** CT colonography (CTC) is an effective test to diagnose and exclude colorectal adenocarcinoma (CRC). However, missed CRC is recognised and defined as post-imaging colorectal cancers (PICRC). We evaluated the frequency and patterns of missed CRC at CTC in an established high-volume service in symptomatic and selected fetal occult blood test/fecal immunochemical test-positive bowel cancer screening cases in comparison with published standards.
**Material and Methods:** All CTC examinations from January 2011 to December 2020 were evaluated and cross-referenced with integrated electronic health records (Patient Pathway Manager—PPM+) for a diagnosis of CRC. PICRC was defined as CRC arising between 6 and 36 months following CTC performed as either a primary or secondary test for CRC. Imaging was evaluated to calculate the PICRC rate per CTC diagnosis of CRC and per 1,000 CTC examinations. Patterns of avoidable PICRC were evaluated.
**Results:** CTC detected 315 CRC when performed as a first test. PICRC occurred in 23 cases with mean delay to diagnosis being 625 days (range 329–1031). The PICRC rate for CTC as a first test was 6.8%, decreasing to 4.9% when including 131 CRC diagnoses known within 30 days prior to CTC. PICRC rate per CTC performed was 0.151% (23 cases per 15,224 CTC). PICRC cases were perceptual error in 48% (11/23), related to patient management in 26% (6/23) and occult in 26% (6/23).
**Conclusion:** PICRC is rare. However, avoidable CRC is present in 74% of PICRC in this series and radiologist education and patient pathway changes should focus on reducing this risk.

### SS 1.3
**Inter-reader agreement in staging colon cancer patients: the usefulness of CECT**
C. Maino[1], M. Ragusi[2], T. Giandola[2], R. Corso[1], C. Talei Franzesi[1]; [1]*Milan/IT*, [2]*Monza/IT*

**Purpose:** To evaluate the diagnostic accuracy of preoperative contrast-enhanced computed tomography (CECT) in local staging of colon cancer.
**Material and Methods:** Patients who underwent preoperative CECT and surgical resection were retrospectively enrolled. Images were reviewed by two independent radiologists, blinded to pathological data. CECT evaluation was based according to the WHO staging system. For T-stage, we included the presence of bowel wall thickness, tumor bulging, perivisceral fat stranding, and vessel enlargement (>2 mm); for the N-stage, we assessed shape (round vs ovoid), enhancement (homogenous vs inhomogeneous), and nodes' clusters (>3 nodes). K-statistics were used to determine the agreement between readers and pathological staging.
**Results:** A total of 80 patients were included (M:F=55:25) with a mean age of 67 (± 12) years. The most common tumor site was the cecum-ascending (n=32, 40%), followed by the descending and sigmoid colon (n=30, 37.5), and the transverse (n=18, 22.5). According to pathological grading, the majority of patients showed a T3 (n=48, 60%), followed by T2 (n=12, 15.0) and T4 (n=11, 13.8), while the majority was N0 (n=48, 60.0), followed by N1 (n=71, 88.7). The agreement between readers was moderate to good for all T parameters, in particular, tumor bulging (κ=0.657), perivisceral fat stranding (κ=0.757), and vessel enlargement (κ=0.610). Similar results were obtained for N-stage, in particular, the shape (κ=0.669), enhancement (κ=0.598), and nodes' clusters (κ=0.710). Overall, the CECT staging obtained showed a moderate agreement with the pathological one (overall κ=0.599).
**Conclusion:** CECT can be considered a reliable tool in staging colon cancer patients, offering detailed information related to histopathology.

### SS 1.4
**Fully automated body composition biomarkers for predicting overall survival at staging CT for colorectal cancer**
S.G. Pickhardt[1], J. Garrett[1], A.A. Perez[1], R. Zea[1], M.H. Lee[1], M.G. Lubner[1], R.M. Summers[2], P.J. Pickhardt[1]; [1]*Madison, WI/US*, [2]*Bethesda, MD/US*

**Purpose:** Staging CT scans performed after colorectal cancer (CRC) diagnosis but before treatment contain potentially valuable body composition data that could further augment its prognostic ability. We utilized automated CT-based quantification of muscle, fat, aortic calcification, and correlated results with overall survival (OS).
**Material and Methods:** Fully automated AI-based CT algorithms for quantifying skeletal muscle density (at L3), total/visceral/subcutaneous fat area (at L1), and abdominal aortic Agatston score were applied to staging abdominal CT scans in 1766 patients (mean age, 63.7±14 years; 862 men, 904 women) diagnosed with CRC. At 5-year longitudinal clinical follow-up, 701 patients had died. Hazard ratios (HR) and receiver operating characteristic (ROC) curve analyses according to survival were performed.
**Results:** Significant differences were observed for all muscle, fat, and calcium measures according to survival (p<0.001). Univariate 5-year ROC-area under the curve (AUC) = 0.648 for L3 muscle HU and 0.654 for aortic calcium score; AUCs for total and visceral fat area were 0.559 and 0.516, respectively. HRs for the highest risk quartile for muscle HU, aortic calcium score, and total fat area were 1.91, 1.80, and 1.41, respectively. Increased fat had a protective survival effect for all fat-based measures. When muscle HU and total fat area are combined, HR and ROC-AUC increased to 2.63 and 0.706, respectively.
**Conclusion:** Fully automated CT body composition tools confirm the importance of sarcopenic myosteatosis for CRC survival, as well as the "obesity paradox" related to the protective effect of abdominal fat. Quantification of atherosclerotic calcification also likely warrants inclusion for overall survival considerations.

### SS 1.5

### Prediction of nodal metastasis in right-sided colon cancer with node for node correlation: a novel approach using complete mesocolic excision with central vascular ligation

M. Widmar, H. Thompson, J.B. Yuval, C. Firat, J. Shia, J. Zheng, M. Capanu, A. Cercek, J. Garcia-Aguilar, M.J. Gollub, D.D. Bates; *New York, NY/US*

**Purpose:** This study evaluated lymph node features predictive of metastases using a novel technique of lymph node correlation between contrast-enhanced CT and surgical specimens with colon cancer.

**Material and Methods:** A group of patients with right-sided colon cancer underwent complete mesocolic excision with central vascular ligation (CME with CVL) over a period of 18 months. Intact gross surgical specimens were compared alongside annotated pre-operative CT images to ensure radiologic–pathologic correlation. CT scans were scored for node short axis, long axis, rounded morphology (short axis/long axis > 0.8), relative enhancement, heterogeneity, and irregular borders. Interobserver agreement was evaluated between two radiologists scoring CT images, and univariate and multivariate analyses were performed.

**Results:** Twenty-six patients (mean age 61, range 30–86; 62% female) were included with a total of 115 mesenteric lymph nodes correlated between CT and pathology, 34 (29.6%) of which were metastatic. Univariate analysis found significant associations between CT node long axis ($p = 0.007$), short axis ($p < 0.001$), and consensus heterogeneity ($p = 0.003$) with node metastases on pathology. The area under the receiver operating characteristic (AUC-ROC) curve was 0.757 for the multivariable model with CT lymph node long axis length and heterogeneity on CT, with a sensitivity of 0.715 and specificity of 0.697. Interobserver agreement for heterogeneity was fair (kappa = 0.387) and for irregular borders was slight (kappa = 0.038).

**Conclusion:** Mesenteric lymph node correlation between CT and pathology is possible for right-sided colon cancers when CME with CVL is performed, and node long axis and heterogeneity yielded an AUC-ROC curve of 0.757 for detecting malignancy.

### SS 1.6

### A new insight into mismatch repair status, systemic inflammatory markers and tumour differentiation and the implications for nodal staging of colon cancer with CT

J. Platt, J. Ansett, J. Seligmann, N. West, D. Tolan; *Leeds/UK*

**Purpose:** Radiological staging in colon cancer (CC) has significant implications for treatment decisions. Mismatch repair (MMR) deficiency accounts for 15–20% of CC diagnoses and is characterised by systemic inflammation and greater local invasion. However, the impact of MMR status on radiological staging is unclear. This study compared radiological staging accuracy between MMR-deficient (dMMR) and MMR-proficient (pMMR) CC.

**Material and Methods:** Radiological and pathological staging were compared for patients who underwent curative resection for CC between April 2019 and May 2020. Staging accuracy was assessed as T and N stages, and as favourable or unfavourable ‘statuses’ (T1/2 vs. T3/4 and N0 vs. N1/2). Incorrect staging was assessed for under- or over-staging. Patient characteristics were analysed for factors to support staging.

**Results:** No difference in overall T or N staging accuracy was seen between 44 dMMR and 57 pMMR tumours. However, dMMR tumours with incorrect N stage or ‘status’ were more likely to be over-staged than pMMR tumours (90% vs. 59%; $P=0.02$ for N ‘status’). Neutrophil count, platelet count, C-reactive protein (area under the curve 0.70 ($P=0.04$), 0.76 ($P=0.007$), and 0.75 ($P=0.03$), respectively) and poor differentiation ($P=0.004$) were associated with N1/2 ‘status’ in dMMR tumours.

**Conclusion:** Whilst T and N staging accuracy were similar between groups, patients with dMMR CC were at significant risk of N over-staging, and potentially inappropriate treatment. We describe novel relationships between inflammatory markers and tumour differentiation and pathological N1/2 ‘status’ in dMMR CC. Integrating these factors into routine assessment has the potential to improve radiological staging and clinical outcomes.

### SS 1.7

### Is CT radiomics superior to morphological evaluation for PN0 characterization? A pilot study in stage II colon adenocarcinoma

I. Nacci, M. Zerunian, M. Polici, D. Caruso, E. Iannicelli, A. Laghi; *Rome/IT*

**Purpose:** Lymph node (LN) involvement is among the most important prognostic factors for patients with colon adenocarcinoma (CAC). However, CT morphological analysis is not a reliable method to assess LN status. Thus, we compared morphological and radiomic features extracted from contrast-enhanced computed tomography (CECT) images, in assessing locoregional LNs in patients with CAC.

**Material and Methods:** This retrospective study included 45 patients with stage II CAC who underwent preoperative portal-venous phase CT examination and diagnosed as pN0 after surgery. Patients with motion artifacts or lack of pathology report were excluded. Locoregional LNs were scored with a qualitative Likert-scale based on morphological imaging (node score with range 0–5) and divided into two groups: low likelihood (0–2 points) and high likelihood (3–5 points) of malignancy. Then 107 radiomic features were extracted from CECT for each LN. T-test and Mann–Whitney were performed to compare radiomic features between the two groups. $P<0.05$ was considered significant.

**Results:** A total of 115 negative LNs were analyzed and divided into 48 with low likelihood and 67 with high likelihood of malignancy based on the node score. Radiomic analysis showed 70 features (5/13 shape, 16/19 first-order and 49/75 second-order features) with no significant difference between the two groups, according to pathology (all $P>0.05$). On the other hand, 37 features (8/13 shape, 3/19 first-order and 26/75 second-order features) were significantly different (all $P<0.04$).

**Conclusion:** Our preliminary experience showed that CT radiomics characterizes locoregional LN status better than CECT morphological evaluation and could be used as a non-invasive preoperative tool in patients with CAC.

### SS 1.8

### MRI staging of colon cancer

S.R. Rafaelsen, C. Dam, C.A. Vagn-Hansen, J. Møller, H. Rahr, M. Sjöström, J. Lindebjerg, T.F. Hansen, M.R. Pedersen; *Vejle/DK*

**Purpose:** The main purpose was to investigate whether MRI could be used in the local staging of colonic cancer.

**Material and Methods:** The study is a prospective blinded observational study. Patients had standard CT scans. For the MRI scan, a 3 Tesla MRI unit was used. Experienced radiologists reported the scans blinded to the other modality and pathological description. Surgery was performed within a median of 7 days after the MDT. The resected specimens were classified according to the pTNM system. The endpoint of the study was the histopathological surgical specimen.

**Results:** From October 2019 to December 2020, 134 patients were included in the study. CT was able to identify 118 of the 134 tumors, whereas MRI identified all tumors. For discriminating T3ab and T3cd cancers, CT had a sensitivity and specificity of 33.3% and 77%, whereas MRI had higher values with 55.6% and 100% ($p<0.05$). The CT had a sensitivity and specificity in lymph node involvement of 59.0% and 56.3%. The same values for MRI were 57.4% and 54.2%, respectively. For the evaluation of extravascular involvement, CT had a sensitivity of 33.3% and specificity of 84.4%, whereas MRI had higher values with a sensitivity of 54.1% and specificity of 77.1%. The mean tumor ADC value tended to be lower in patients with distant metastases compared to those without. Tumors containing mucin had statistically significantly less diffusion restriction.

**Conclusion:** MRI had higher sensitivity compared to CT in detecting advanced T3 tumors. Diffusion-weighted MRI was supportive in the diagnosis of mucin in colonic tumors.

### SS 1.9

### Performance evaluation of CT as a biomarker for neoadjuvant chemotherapy in colon cancer: FOxTROT experience

D. Tolan[1], D. Morton[2], M. Seymour[1], P. Quirke[1], K. Handley[2], L. Magill[2], N. West[1], J. Seligmann[1]; [1]*Leeds/UK,* [2]*Birmingham/UK*

**Purpose:** Neoadjuvant chemotherapy improves outcomes in patients with advanced colon cancer. Imaging with standard abdominopelvic CT is a tool for stratifying patients to benefit from this treatment. Trained radiologist investigators in the FOxTROT trial identified patients with locally advanced tumours (T3 or T4, N (any), extramural vascular invasion (EMVI) (any), M0). T1 and T2 tumours by radiological assessment were excluded. Cases were then allocated to either straight to surgery or two cycles of neoadjuvant chemotherapy in a 1:2 randomisation. We analysed the straight to surgery cohort to determine whether CT reliably detects advanced cancers and avoids over treatment for early tumours.
**Material and Methods:** 354 patients were randomised straight to surgery. The reported radiology variables were compared to the final pathology variables.
**Results:** CT had an accuracy of detecting tumours either extending beyond the bowel wall and/or with malignant nodes and/or with extramural venous invasion of 96.7% (323/334) with T1/T2 N0 EMVI-ve tumours present in 11/334 cases (3.3%). Radiological tumour thickness was associated with extension beyond the muscularis propria (Chi square 27.4072, p=<.0001) and radiological extension beyond the muscularis propria was correlated with pathological distance beyond the muscularis propria (r-0.423, p<0001).
**Conclusion:** CT is an effective biomarker to select higher risk patients with advanced colon cancer that benefit from neoadjuvant chemotherapy.

**09:00 - 10:30** **Auditorium 8**

*Scientific Session Live SS 2*

### Pancreatic adenocarcinoma

### SS 2.1

### Higher volume growth rate is associated with the development of worrisome features in patients with branch duct-intraductal papillary mucinous neoplasms

F. Fedeli, D. Palatresi, G. Danti, T. Innocenti, A. Galli, V. Miele; *Florence/IT*

**Purpose:** Branch duct-intraductal papillary mucinous neoplasms (BD-IPMNs) are common pancreatic cystic tumours with low risk of malignant transformation. Current guidelines do not consider cyst volume and its correlation with risk of malignant degeneration, but only diameter. The purpose of our study is to assess both BD-IPMNs' diameter and volume growth rate and evaluate their correlation with the development of malignant characteristics.
**Material and Methods:** We retrospectively reviewed CT and MRI exams of patients with a known BD-IPMN and at least two contrast-enhanced studies at our centre and a 12-month minimum follow-up time. The diametral size was measured along the 3 major diameters; the volume expressed in $cm^3$ was calculated by a manual region of interest segmentation along the edge of the neoplasm. Changes in size over time were measured, and the development of worrisome features and high-risk stigmata was evaluated.
**Results:** About 98 patients were evaluated across a 40.5-month median follow-up time, of which 10 developed worrisome features. In patients who developed worrisome features, cysts at baseline were significantly larger (diameters p=0.0035, p=0.00652, p=0.00424; volume p=0.00222). Volume growth rate was significantly higher too (1.12 vs 0 $cm^3$/year, p=0.0001); diameter growth rate was higher, but the difference did not always reach statistical significance. In addition, first-year follow-up volume but not diameter growth rate was higher in patients who developed worrisome features (0.46 vs 0 $cm^3$/year, p=0.00634).
**Conclusion:** The measurement of baseline volume and its variation over time is a reliable tool for the follow-up of BD-IPMNs. Volume measurement could be a better tool than diameter measurement to predict the development of worrisome features.

### SS 2.2

### Preclinical diffusion tensor imaging of the pancreas for the characterization of pre-malignant lesions

C. Bilreiro, F.F. Fernandes, R.V. Simões, A. Ianus, C. Chavarrias, M. Castillo-Martin, T. Carvalho, C. Matos, N. Shemesh; *Lisbon/PT*

**Purpose:** To explore diffusion tensor imaging (DTI) metrics for the identification and characterization of pancreatic pre-malignant lesions, namely pancreatic intraepithelial neoplasia (PanIN).
**Material and Methods:** Genetically engineered mouse models (GEMM) developing pancreatic cancer and pre-malignant lesions were used: N=10 KC (Pdx1-Cre, LSL-Kras$^{G12D}$/Ptf1a-Cre, LSL-Kras$^{G12D}$) and N=10 KPC mice (Ptf1a-Cre, LSL-Kras$^{G12D}$, p53$^{LoxP}$). The animals were imaged periodically in a 9.4T scanner, until pancreatic abnormalities were seen in T2WI. Pancreases from these animals and 5 healthy controls were removed and MR-microscopy was performed on a 16.4T scanner using a novel diffusion-weighted GRE pulse sequence, providing ultrahigh-resolution 3D images with both diffusion and T2* weighting. Histology (H&E) slides were co-registered with MRI for identifying contrasts sensitive for PanIN. The method was adapted for in vivo imaging (9.4T scanner) on N=6 KPC and N=6 control mice. Finally, N=5 pancreatic specimens obtained from human patients subjected to pancreatic cancer resection underwent the MR-microscopy protocol.
**Results:** Fractional anisotropy (FA) maps from the MR-microscopy approach revealed significantly higher FA values in PanIN lesions (histologically validated) compared with normal mouse pancreas (medians: 0.2 vs. 0.1, p<0.0001). We confirmed these findings when imaging live animals, deconfounding possible fixation artifacts and providing a practical application for the non-invasive characterization of PanIN in GEMM. Finally, results obtained with human samples corroborated the findings in mice, providing initial insight into the imaging characterization of PanIN using microstructural modeling approaches.
**Conclusion:** FA, a DTI-derived metric, can be used to detect and characterize PanIN in GEMM and in human pancreatic samples. Our findings hold promise for future clinical applications of diffusion-driven metrics for pancreatic pre-malignant lesions' characterization.

### SS 2.3

### Efficacy of contrast-enhanced magnetic resonance imaging in the differentiation between malignant and benign pancreatic cystic neoplasms

A. Kristic, A. Messner, N. Bastati-Huber, S. Poetter-Lang, R. Ambros, M. Schindl, A. Ba-Ssalamah; *Vienna/AT*

**Purpose:** To evaluate the efficacy of contrast-enhanced magnetic resonance imaging (CE-MRI) in the differentiation between malignant and benign pancreatic cystic neoplasms (PCNs).
**Material and Methods:** We included 101 patients (62 ± 14 years), who underwent, dynamic, CE-MRI. The final diagnosis was histopathologically proven either after surgery (n = 75) or by endoscopic, ultrasound-guided, fine needle aspiration (n = 26). The imaging features of PCNs were retrospectively reviewed and the total number of pancreatic cysts and the maximum diameter, location, and characteristics of the cystic lesion were recorded, including worrisome features or high-risk stigmata, such as 1) size of the cyst (more than 30mm), 2) duct dilatation 3) mural nodules and 4) solid components. The final diagnosis, based on MRI, was compared to histopathology, using the Cohen's kappa coefficient.
**Results:** In 77 (76%) of 101 patients, MRI features provided an accurate diagnosis, leading to a Cohen's kappa agreement κ=0,760. The presence of at least one worrisome feature (WF) and/or high-risk stigmata (HRS) revealed a sensitivity of 89%, a specificity of 38%, a PPV of 62%, and an NPV of 75%. The diagnostic accuracy when three or more WF and/or HRS were present showed a sensitivity of 71%, a specificity of 78%, a PPV of 75%, and an NPV of 75%. The presence of at least one HRS revealed a sensitivity of 41%, a specificity of 77%, a PPV of 67%, and an NPV of 53%.
**Conclusion:** CE-MRI is a valuable non-invasive diagnostic tool that can differentiate malignant and benign PCNs.

### SS 2.4

### Coronary artery calcium score as a predictor of major adverse cardiac events in patients undergoing pancreatic surgery

B.I. van Rosmalen, M.A. Brouwer, A.M. Hogenes, M. Meij, M. Van der Kolk, C.J. Van Laarhoven, M. Prokop, J.J. Hermans; *Nijmegen/NL*

**Purpose:** Major adverse cardiac events (MACE) constitute 30% of peri-operative risk after pancreatic surgery. The currently used revised cardiac risk index (RCRI) is complex and time consuming. Qualitative coronary artery calcium (CAC) scoring on routine low-dose contrast-enhanced computed tomography (CECT) is an alternative to quantitative assessment on computed tomography coronary angiography (CTCA). We investigated whether CAC score predicts MACE within 30 days after pancreatic surgery.
**Material and Methods:** Consecutive patients from the Dutch Pancreatic Cancer Audit dataset were included. Non-electrocardiographic-gated low-dose CECT scans were assessed with a 5-point scale: 0 = no calcification, 1 = mild calcification, 2 = moderate calcification, 3 = severe calcification, 4 = extreme calcification. The overall coronary CAC score per patient was assessed. We determined the predictive accuracy of the CAC score in relation to MACE, independent of covariates (baseline variables, peri-operative data) and compared it to RCRI. We explored the optimal pre-operative risk model.
**Results:** MACE occurred in 21.8% (50/232). The mean CAC score was 1.19. In patients with MACE, the mean CAC score was higher than in those without MACE (2.08 resp. 0.95). After adjustment for BMI, previous MI and dyslipidemia, the CAC score was independently associated with MACE (OR 2.35 (1.65–3.35 95%CI)). Predictive accuracy of the CAC score only was significantly higher than the RCRI ((AUC=0.757 (p=0.001) resp. AUC=0.568 (p=0.145)). The optimal risk model consisted of BMI, previous MI, dyslipidemia and the CAC score (AUC=0.780, p=0.001).
**Conclusion:** In patients undergoing pancreatic surgery, CAC score assessment on low-dose CECT showed a superior predictive accuracy for MACE compared to the currently used RCRI.

### SS 2.5

### A CT-based radiomics study using tumour and vessel features to predict non-resectability in chemonaive patients with cancer of the pancreatic head

G. Litjens[1], J.J. Hermans[1], J. Broekmans[2], T. Boers[2], M. van den Hurk[1], D. Ozdemir[1], C. Schaik[1], M. Janse[3], E. van Geenen[1], K. van Laarhoven[1], M. Prokop[1], H. Huisman[1], P. de With[2], F. van der Sommen[2]; *[1]Nijmegen/NL, [2]Eindhoven/NL, [3]Utrecht/NL*

**Purpose:** Surgical resection is the only potential cure for patients with pancreatic ductal adenocarcinoma (PDAC). However, about 25% is unexpectedly unresectable during surgery. Radiomics could improve the prediction of non-resectability and prevent futile surgery.
**Material and Methods:** Retrospective single-center study on chemonaïve pancreatic head PDAC. Tumour, surrounding veins, and arteries were segmented. Stable PyRadiomics and tumour-vessel contact features were selected based on an interobserver study (3 radiologists, 9 scans), collinearity, and LASSO. Three support vector machine models (5-fold cross-validation) were built to predict non-resectability, using tumour features, vessel features and a combination of both. Optimal point of the ROC curve was determined with MATLAB perfcurve method. Results were validated with a test set. Pancreatic surgery was performed in pairs by four experienced pancreatic surgeons following a strict protocol.
**Results:** 88 patients were included in the training set and 15 in the test set. The best performing model (vessel features only) reached an AUC of 0.89 with a sensitivity, specificity, positive predictive value, and negative predictive value of 58%, 96%, 90%, and 81%, respectively. Validation with the test set showed a sensitivity of 100% and specificity of 88%. Prediction of non-resectability by the multidisciplinary team (MDT) reached a significantly higher sensitivity (97%, p<0.001), but a lower specificity (72%, p<0.001).
**Conclusion:** We developed three radiomics models based on tumour and vessel features that were able to accurately predict non-resectability in chemonaïve patients with pancreatic head cancer. The high specificity of the model could aid in preventing futile surgery. Prediction by the MDT and the model are complementary.

### SS 2.6

### Diagnostic performance of MRI to predict the resectability of pancreatic adenocarcinoma according to National Comprehensive Cancer Network criteria

L. Soydan, C. Karabiber Deveci; *Istanbul/TR*

**Purpose:** To evaluate the diagnostic performance of MR to predict the possibility of margin-negative resection (R0) in patients with pancreatic adenocarcinoma (PAC) according to National Comprehensive Cancer Network (NCCN) criteria.

**Material and Methods:** Preoperative MR images of PAC patients who underwent upfront surgery with the intent of curative resection between January 2013 and March 2017 were retrospectively reviewed by two radiologists to assess preoperative resectability (resectable, borderline resectable or unresectable) according to the NCCN criteria. The study group consisted of 50 PAC patients (mean age, 62.3 years ±11.64). The association between R0 resection rates and MR-determined resectability status was evaluated. Factors associated with R0 resection were identified by logistic regression analysis.

**Results:** None of the patients in the study group was interpreted as unresectable by both readers. In 50 patients who underwent surgery, R0 resection rates for resectable and borderline resectable disease were for Reader 1: 74.2% (23/31), 42.1% (8/19) and for Reader 2: 72.4% (21/29), 47.6% (10/21), respectively (p <.005), with a very good inter-reader agreement (k: 0.75). Combined sensitivity, specificity, positive predictive value and accuracy of MR to predict R0 resection in MR-resectable patients were 84.4%, 26.5%, 74.1% and 68%, respectively. At multivariable analysis, tumor diameter >4 cm on preoperative MR was significantly associated with margin-positive (R1/R2) resection and emerged as an independent predictor for R1/R2 in operated patients (p =0.048; 1). R0 resection rates were 90% (9/10) for tumors <2 cm and 33.3% (2/6) for tumors >4 cm.

**Conclusion:** MR may be used to identify PAC patients who have a higher likelihood of R0 resection. Our study results showed that larger tumor size is associated with margin-positive resection in patients with resectable pancreatic cancer.

### SS 2.7

### Pancreatic ductal adenocarcinoma: CT texture analysis of the area lesion to predict metastasis

L. Geraci, L. Tomaiuolo, C. Longo, D. Autelitano, M. Todesco, A. Carli, F. Verrengia, A. Olivieri, R. De Robertis, M. D'Onofrio; *Verona/IT*

**Purpose:** The aim of this study is to evaluate the presence of metastasis through CT texture analysis in patients with pancreatic ductal adenocarcinoma (PDAC).

**Material and Methods:** 3 cohorts of patients were retrospectively enrolled: 1218 with anatomopathological diagnosis of PDAC; 1383 received surgery of head pancreatic cancer; 1260 with head pancreatic cancer extracted from our radiological archive. Essential for each one were arterial and venous phases in CT examination available in DICOM format; clinical and biological parameters including CA19.9, CEA, CA125, AST, ALT, GGT and bilirubin levels were recorded. Texture features were extracted from 2D regions of interest (ROIs) based on unenhanced, arterial- and portal-phase CT images and compared with the presence of metastasis. Clinical features and texture-derived parameters were compared; receiver operating characteristic (ROC) curves were constructed for the features that showed a significant difference between groups. Kruskal–Wallis and ANOVA tests were used to assess differences of texture parameters between metastatic (M+) and non-metastatic (M−) groups.

**Results:** 321 patients (167 males, 154 females; age range, 30–87 years) were included. Several features were different with statistical significance. Arterial and venous HU_min and HU_mean were significantly minor in M+ (p = 0.00 for all). Arterial and venous SHAPE_Volume_(ml) were significantly greater in M+ (p = 0.00 both). Furthermore, significant differences were found with p <0.05 in other 7 first-order features, in 3 venous GLCM features, in 12 GLRLM features, in 6 NGLDM features, and in 9 GLZLM features.

**Conclusion:** CT texture analysis could predict PDAC metastasis through several parameters with statistically significant differences between M+ and M− groups.

### SS 2.8

### Radiogenomics of pancreatic ductal adenocarcinoma

C. Longo, L. Tomaiuolo, L. Geraci, G. Aluffi, M. Todesco, D. Autelitano, V. Rossi, F. Cicalo', M. D'Onofrio; *Verona/IT*

**Purpose:** Certain mutated genes in pancreatic ductal adenocarcinoma (PDAC) carcinogenesis are KRAS, CDKN2a/INK4a, TP53 and DPC4/SMAD4. The aim of our study is to find correlations between CT and MR texture analysis parameters with PDACs mutated genes, in particular with KRAS and TP53 expression.

**Material and Methods:** 30 patients with PDAC diagnosis between 2010 and 2016 were enrolled retrospectively for CT/MR imaging and genomic studies. Two independent radiologists analysed the radiomic parameters. 47 texture features were extracted from 3D regions of interest (ROIs) based on unenhanced, arterial-, portal- and tardive-phase in CT and MR images. The genetic study was performed at Verona Research Center. For the correlation between variables, the following tests were used: Chi-square test or Fisher's test for qualitative parameters; Chi-square test or Spearman's Rho test for quantitative parameters; T Student's test for parametric variables and Matt–Whitney test for non-parametric variables.

**Results:** Texture analysis in PDACs showed that in MRI and CT, the first-order parameters (CONVENTIONAL_HUmax, HISTO_Skewness and SHAPE_Volume) are related to the percentage of KRAS mutations while other first-order parameters (CONVENTIONAL_min; _mean; _Q1; _Q2; _Q3, HISTO_Skewness [r= −0.854]) are related to TP53 mutations. The second-order parameters (GLZLM [grey-level zone length matrix] and GLRLM [grey-level run-length matrix]) are highly correlated with the presence and increase of TP53 mutations in venous CT, T1 Fat Sat sequences (both pre- and post-contrast) and in T2 Fat Sat sequences (respectively, r = -0.903 and r= -0.854).

**Conclusion:** This study looks for radiogenomic data for PDACs through MRI and our results underline the power of second-order statistical parameters in PDAC radiogenomics.

### SS 2.9

### Comparison between CT texture data, resection margin status and clinical outcomes in pancreatic adenocarcinoma resected after induction chemotherapy and neoadjuvant stereotactic body radiation therapy

R. De Robertis, L. Geraci, L. Tomaiuolo, D. Autelitano, M. Todesco, A. Carli, C. Longo, M. D'Onofrio; *Verona/IT*

**Purpose:** The aim of this study is to compare CT texture analysis parameters with the resection margin status, the recurrence-free survival (RFS) and the overall survival (OS) in patients with pancreatic ductal adenocarcinoma (PDAC) resected after induction chemotherapy and neoadjuvant stereotactic body radiotherapy (SBRT). Preoperative prediction of patients who are likely to have positive resection margins and worse clinical outcome is beneficial to avoid early disease recurrence after surgery.

**Material and Methods:** Between January 2017 and May 2019, 134 patients with histologically proven unresectable PDAC underwent induction chemotherapy (FOLFIRINOX or Gemcitabine + Nab-Paclitaxel), followed by radiotherapy. Patients who received surgery after neoadjuvant SBRT were included. 47 texture features were extracted from 3D regions of interest (ROIs) based on unenhanced, arterial- and portal-phase CT images performed for SBRT planning. Clinical features and texture-derived parameters were compared; receiver operating characteristic (ROC) curves were constructed for the features that showed a significant difference between groups. Cox regression analysis and Kaplan–Meier curves were used to determine the association of clinical–pathological variables and texture parameters with RFS and OS.

**Results:** 43 patients (27 males, 16 females; mean age, 65 years; age range, 48–81 years) were included in this retrospective study. 14 patients (32.6%) had vascular resection. Patients with higher arterial HU_Q1 had significantly shorter RFS than patients who did not meet this criterion (p=.032 and .005; mean RFS 9.6 vs 14.8 months and 8 vs 14.3 months, respectively); no significant predictors were found for OS.

**Conclusion:** CT texture analysis could predict RFS in patients with PDAC resected after induction chemotherapy and neoadjuvant SBRT.

### SS 2.10

### Diffusion-weighted magnetic resonance imaging in restaging of pancreatic ductal adenocarcinoma after neoadjuvant therapy: a pilot study

B. Miseur, V. Vandecaveye, L. Cockmartin, B. Topal, E. Van Cutsem, T. Roskams, R. Dresen; *Leuven/BE*

**Purpose:** To investigate the utility of diffusion-weighted magnetic resonance imaging (DWI-MRI) to predict operability (R-status) and survival of patients with borderline resectable (BR) and locally advanced (LA) pancreatic ductal adenocarcinoma (PDAC) after neoadjuvant chemotherapy (NACT).

**Material and Methods:** Two readers retrospectively analysed 25 consecutive patients with BR/LA-PDAC who underwent restaging DW-MRI after NACT prior to surgery. Interreader agreement was assessed using intraclass correlation coefficient (ICC). Whole tumor apparent diffusion coefficient (ADC) was correlated with 1-year overall survival with a cut-off value determined by receiver-operator characteristic (ROC) curve. Additionally, for each vessel circumferential extent-, change- and length of contact, stenosis, contour irregularity/attraction, perivascular ADC value, qualitative degree- and circumferential extent of diffusion restriction were determined. Chi-square test or regression analysis was performed to determine correlation between these features and the need for vascular resection.

**Results:** Interobserver agreement for whole-tumor ADC was moderate (0.650). Post-NACT whole tumor ADC was significantly higher (p=0.034) for patients with 1-year overall survival post-surgery with cut-off value of $1.278*10^{-3}$ mm²/s and area under the curve (AUC) of 0.822. No arterial resections were performed, 10/21 operable patients required venous resection. Circumferential extent of soft tissue contact, stenosis, contour irregularity/attraction, change in vascular contact and perivascular ADC value were associated (p<0,05) with a need for venous resection, of these perivascular ADC values less than 1,480*10-3 mm³/s were most accurate (AUC 0.815).

**Conclusion:** Post-NACT tumor mean ADC was predictive for postoperative survival. Addition of DW-MRI features may result in more accurate preoperative restaging of PDAC patients after NACT.

**11:00 - 12:30** **Auditorium 3 + 4**

*Scientific Session Live SS 3*

### Rectal cancer staging

### SS 3.1

### Rectal MRI quality assessment by radiographers: do they see it the same way as a radiologist?

D. Tolan[1], J. Taylor[1], H. Rossington[1], D. Lambregts[2], P. Quirke[1], P.J. Brown[3]; *[1]Leeds/UK, [2]Amsterdam/NL, [3]York/UK*

**Purpose:** Variation of interpretation content and quality in rectal cancer staging MRI (RC-MRI) is well recognised. RC-MRI image quality (IQ) is also critical. We evaluated IQ assessment by experienced radiographers performing RC-MRI in routine practice.

**Material and Methods:** 20 experienced MRI radiographers from 13 hospitals serving a population of 5.2 million attended 90 minutes of tutorials on clinical and technical aspects of RC-MRI provided by expert radiologists and radiographers. Subsequently, they were asked to qualitatively evaluate 8 RC-MRI scans on workstations by rating 7 aspects including clarity of tumour location; scan centring on tumour; effect of artefacts; tumour coverage and suitability of scan planes. They used a subjective 5-point scale (4=excellent to 0=non-diagnostic), converted to a score out of 100. A GI radiologist with 15 years' experience was the gold standard (GS). One sample T test assessed differences between radiographers versus GS.

**Results:** Assessment was significantly different in 50% (4/8) of cases between GS and group mean radiographer score with 3 mean IQ 'over-estimations' and 1 mean IQ 'under-estimation' (p ≤0.0014). 40.9% of all case assessments were >25% from the GS (range 0.0% to 90.5% per case). The median per radiographer cumulative percentage difference from GS was 44% (range 21–73%) with 8/20 radiographers more than 50% from GS.

**Conclusion:** Radiographer's evaluation of RC-MRI IQ is highly variable, despite focused prior training and clinical experience. This should be addressed in any programme to improve the quality of RC-MRI in patient care.

### SS 3.2

### Value of pelvic CT in addition to MRI to differentiate between rectal and sigmoid cancer on imaging using the sigmoid take-off as a landmark

N. Bogveradze[1], M. Maas[1], N. El Khababi[1], N.W. Schurink[1], M.J. Lahaye[1], F. Bakers[2], P.J. Tanis[1], M. Kusters[1], G.L. Beets[1], R.G.H. Beets-Tan[1], D. Lambregts[1]; *[1]Amsterdam/NL, [2]Maastricht/NL*

**Purpose:** The sigmoid take-off (STO) was recently introduced as a preferred landmark to discriminate between rectal and sigmoid cancer on MRI. A previous study has shown that the varying angulation of typically oblique-axial imaging planes on MRI is a potential pitfall. Aim was to establish the benefit of adding a true-axial CT plane to the MRI assessment.

**Material and Methods:** A senior and junior radiologist retrospectively re-classified n=40 patients (clinically staged as rectal/rectosigmoid cancers) using the STO as a landmark; first on MRI-only (sagittal and oblique-axial) and then using a combination of MRI and true-axial portal-venous phase CT. Tumours were classified as rectal/rectosigmoid/sigmoid (according to originally published STO definitions) and then dichotomized into rectal vs. sigmoid. Diagnostic confidence was documented using a 5-point scale.

**Results:** Addition of CT impacted tumour localization in 15% (6/40) for the junior and 10% (4/40) for the senior reader. Diagnostic confidence increased significantly after addition of CT for the junior reader (p<0.001) to a level similar to that of the senior reader. Confidence of the senior reader was not affected by the addition of CT. Interobserver agreement was similarly good for MRI-only (κ=0.77) versus MRI+CT (k=0.76). The two readers reached consensus on the diagnosis of rectal versus sigmoid cancer in 78–85% of the cases.

**Conclusion:** Availability of a true-axial imaging plane — in the case of this study provided by CT — in addition to a standard MRI protocol with sagittal and oblique-axial imaging views can be helpful to more confidently localize tumours using the STO as a landmark, especially for more junior readers.

### SS 3.3

### MRI staging of ano-rectal malignancy — a reporting dilemma: is it adenocarcinoma or squamous cell carcinoma?

A. Chandramohan, K. Sathyakumar, A. Eapen, R. Mittal; *Vellore/IN*

**Purpose:** MRI of ano-rectal malignancy is reported assuming low rectal adenocarcinoma (LRC). However, when biopsy reveals squamous cell carcinoma (SCC), the stage and management significantly change. Thus, the aim was to compare the imaging findings of SCC and LRC.
**Material and Methods:** This was a retrospective study of patients who underwent staging MRI for ano-rectal malignancy (<5 cm from anal verge) for adenocarcinoma or squamous cell carcinoma between 2016 and 2021. Two radiologists blinded to biopsy reviewed MRI. Imaging findings, three ADC values each and pattern of nodal metastases were compared among SCC and LRC.
**Results:** Total of 137 patients (n=60 SCC and n=77 LRC) with mean age of 50.4+/−12.4 years were included. Mean tumour length was 5.6+/−1.9 cm; majority (>80%) had infiltrating mass with no difference between SCC and LRC. Intermediate signal and diffusion restriction were seen in 97% and 98.2% of SCC vs. 75.3% and 77% of LRC, respectively. ICC coefficient (95% CI) for ADC value was 0.942 (0.918-0.959), p<0.001. SCC had significantly lower ADC value (0.910 x $10^{-3}$ $mm^2/s$) compared to LRC (1.126x$10^{-3}$ $mm^2/s$), p<0.001. But there was no difference in ADC after excluding T2 hyperintense LRC, p=0.132. LRC had more EMVI (35.1%) vs. 16.7% in SCC. Metastases to mesorectal nodes were similar, but internal iliac (46.7% vs 28.6%), external iliac (20% vs 4%) and inguinal nodes (36.7% vs 5.2%) were more common among SCC, p<0.05.
**Conclusion:** Morphology and ADC values were unhelpful in differentiating SCC and LRC. But EMVI may favour the diagnosis of adenocarcinoma, and significant inguinal, internal-iliac and external-iliac nodes may point towards squamous cell carcinoma.

### SS 3.4

### Staging of early rectal cancer, the diagnostic performance of MRI to identify tumors eligible for local excision

E. Viktil; *Oslo/NO*

**Purpose:** To test the diagnostic performance of MRI for early rectal cancers (ERC) when adding a pre-procedural micro-enema and concurrent use of a modified classification system. The micro-enema (Bisacodyl) induces submucosal edema and may hypothetically improve the visualization of tumor depth in submucosa. In addition, we also evaluated the lymph nodes as part of the preoperative staging.
**Material and Methods:** In this prospective study, we consecutively included 73 patients with newly diagnosed rectal tumors. Two experienced radiologists independently interpreted the MRI examinations and classified the tumors as eligible for local excision (LE) (Tis-T1sm2, n=43) or as too advanced for LE (T1sm3-T3b, n=30). Each reader registered sensitivity, specificity, PPV and NPV. Inter- and intrareader agreement were assessed by kappa statistics. Lymph node status was retrieved from the clinical MRI reports.
**Results:** When using the modified classification system, reader1/reader2 achieved sensitivities, specificities, PPV and NPV of 93%/86%, 90%/83%, 93%/88% and 90%/81%, respectively, for identifying tumors eligible for LE. Rates of over-staging of local tumors were 7% and 14%. Kappa values for the inter-and intrareader agreement were 0.69 and 0.80. For tumors ≤T2, metastatic lymph nodes or nodal tumor deposits were smaller than 3 mm.
**Conclusion:** MRI supplemented with a pre-procedure rectal micro-enema and concurrent use of a modified staging system achieved good diagnostic performance to identify tumors suitable for LE. The rate of over-staging of local tumors was comparable to results reported in previous endorectal ultrasound (ERUS) studies. For tumors ≤ T2, metastatic lymph nodes were too small to be characterized by MRI.

### SS 3.5

### High-resolution diffusion-weighted imaging for the evaluation of extramural tumor invasion in primary rectal cancer

S.H. Kim, E.J. Park, S.J. Jo, J.-H. Yoon; *Busan/KR*

**Purpose:** To determine the added value of high-resolution diffusion-weighted imaging (DWI) to T2-weighted imaging (T2WI) for the evaluation of extramural tumor invasion (EMTI) in patients with primary rectal cancer.
**Material and Methods:** Seventy-eight patients (51 men, 27 women; mean age, 67 years; range, 49–87 years) who had undergone 3.0-T MRI including high-resolution DWI ($b$=0, 1000 $s/mm^2$, 2mm iso-voxel) and subsequent surgery were included in this study. To evaluate the added value of DWI, two blinded radiologists independently read the T2WI first and then the combined (T2WI+DWI) set. They recorded their confidence level using a 5-point scale of EMTI. The diagnostic accuracy for each reviewer was calculated by receiver operating characteristic (ROC) curve analysis. The added value of DWI to T2WI for EMTI evaluation was determined by pair-wise comparison of the ROC curves. The histopathological results served as the reference standard for EMTI.
**Results:** The study population consisted of T1 (n=9), T2 (n=25), T3 (n=38) and T4 (n=6) patients. The area under the ROC curve (AUC) was not significantly increased after adding DWI to T2WI (for reader 1, 0.868 to 0.856, $P$=0.5618; for reader 2, 0.848 to 0.865, $P$=0.4539). The sensitivity and specificity of both readers also did not show significant difference (reader 1, 86% to 91% and 82% to 62%; reader 2, 77% to 82%, 76% to 79%).
**Conclusion:** Adding high-resolution DWI to T2WI showed no additional diagnostic value for the evaluation of EMTI in patients with primary rectal cancer.

### SS 3.7

### Impact of a quality improvement programme on rectal cancer MRI reporting in clinical practice: does it improve report content?

C. Muthoo[1], P.J. Brown[1], H. Rossington[2], S. Alderson[2], P. Quirke[2], D. Tolan[2]; [1]*York/UK*, [2]*Leeds/UK*

**Purpose:** Complete rectal cancer MRI (RC-MRI) reports are essential for colorectal cancer multi-disciplinary team (CRC-MDT) treatment planning. While template-style reporting improves reporting standards, adoption is not widespread, leading to variable content and completeness. We assessed clinical practice in specialist GI radiologists against a previous audit in 2016 to measure the change in quality of rectal cancer staging MRI reports in a regional quality improvement programme.
**Material and Methods:** Thirteen English CRC-MDTs serving a 5.2 million population submitted up to 10 consecutive RC-MRI reports per radiologist. Reports were compared to reference standard tumour features, prognostic features and prior audit data and the study was supported by Yorkshire Cancer Research-funded Bowel Cancer Improvement Programme L394.
**Results:** 318 reports were submitted from 33 of 42 (78.6%) eligible radiologists; 301 met inclusion criteria with 156 (51.8%) using templates. Template use significantly increased recording of key descriptors versus non-template reports for extra-mural venous invasion (EMVI) (100% vs 71.7%, p < 0.001) and circumferential resection margin (CRM) (98.1% vs 81.4%, p < 0.001). Local tumour stage (100% vs 99.3%, NS) and nodal stage (100% vs 99.3%, NS) were reported with similar frequency. Template-style reporting adoption (51.8% vs 22.5%, p < 0.001), recording of EMVI status (86.4% vs 62.2%, p < 0.001) and CRM status (90.0% vs 72.8%, p < 0.001) improved compared with 2016 data.
**Conclusion:** Template reporting significantly increases inclusion of key tumour descriptors. While a dedicated quality improvement programme has improved reporting of key tumour descriptors and adoption of template reporting, significant deficiencies persist.

### SS 3.8

### Upper abdominal imaging in rectal cancer staging MRI

C. Muthoo, M. Ting, J.D. Coates, P.J. Brown; *York/UK*

**Purpose:** Rectal cancer MRI staging (RectMR) at our institution has historically included an upper abdominal half-Fourier acquisition single-shot turbo spin-echo (UA-HASTE) sequence alongside pelvic sequences for all patients with rectal cancer in an attempt to characterise liver lesions, avoiding the need for a dedicated liver MRI (LiverMR) in some cases. This audit aimed to give an idea of sensitivity for this sequence and to assess the associated 'time cost'.
**Material and Methods:** 100 sequential RectMR cases from 23/11/19 were reviewed. Accurate identification of liver metastases on the staging CT and RectMR was compared to the final staging (which included LiverMR if required). Time cost was calculated based on LiverMR requiring 45 minutes versus UA-HASTE adding 5 minutes per RectMR.
**Results:** RectMR identified 18 liver lesions. 17 cases had staging CT scans (1 case was a benign rectal polyp). 12 of 17 cases were characterised as benign or malignant, 3 as indeterminate and 2 were not seen/reported on the staging CT. 1 of 3 indeterminate lesions was confirmed to be a simple cyst on RectMR, while 2 of 3 required a LiverMR (1 then proven as benign, 1 malignant). No liver metastases were identified on RectMR but missed by staging CT. Sensitivity and specificity were 1.0 and 0.97 for CT and 0.78 and 0.98 for RectMR. Omitting UA-HASTE from RectMR but performing LiverMR on select cases would result in a time saving of 365 minutes per 100 RectMR.
**Conclusion:** Omitting UA-HASTE from RectMR is safe and offers a significant time saving even accounting for selective LiverMR.

### SS 3.9

### Accuracy of endorectal ultrasound (EUS) in rectal cancer and the impact this has on management

A.O. Yousif[1], N. Jagirdar[2], E. Teodorescu-Arghezi[2];
*[1]Leeds/UK, [2]Bradford/UK*

**Purpose:** Accurate staging of rectal cancer is central to patient management and has both therapeutic and prognostic implications. Patient selection for minimally invasive surgical techniques requires accurate tumour depth assessment for T staging, which is best assessed at EUS. This study was undertaken to evaluate correspondence between EUS staging and the final histology in patients with early rectal cancer.
**Material and Methods:** This was a retrospective study looking at the final histological correlation between all EUS studies performed at the regional rectal cancer hospital over a 24-month period between Jan 2019 and Dec 2020. Patients were discussed at multidisciplinary team meeting and referred for EUS. Colonoscopy and MRI studies were reviewed and correlated with final histology in those patients proceeding to surgery.
**Results:** 71 patients with early rectal cancers/polyps were referred for endoscopic ultrasound. 8 patients were excluded. A total of 63 patients were included in the final analysis. In 59 (94%) patients, EUS findings of lesions confined to the mucosa/submucosa were concordant with the final histology. 35 (56%) of these were polyps with dysplasia or adenomas and 23 (37%) were early rectal cancers. 1 patient had T2 with focal T3 disease which was downstaged with neoadjuvant treatment with the final histology being pT2 with focal T3 extension.
**Conclusion:** EUS is a useful adjunct to MRI for accurate locoregional staging of early rectal cancers. It serves to increase clinician confidence and aids patient selection for organ preserving minimally invasive surgical techniques with a high degree of concordance on final histology.

### SS 3.10

### MRI surveillance after treatment with contact X-ray brachytherapy for rectal cancer: preliminary results

P. Custers, M. Maas, D. Lambregts, R.G.H. Beets-Tan, I. Huibregtse, M. van Leerdam, D. van der Reijd, G.L. Beets, F. Peters, C. Marijnen, B. van Triest; *Amsterdam/NL*

**Purpose:** To describe MRI characteristics during follow-up of rectal cancer patients treated with contact X-ray brachytherapy (CXB) after (chemo)radiotherapy aiming at organ-preservation.
**Material and Methods:** Between December 2017 and May 2020, 28 patients were treated with CXB after a delayed interval following short-course radiotherapy or chemoradiation. Three expert readers scored the pre-CXB and sequential post-CXB MRI scans (performed every three months in the first two years and every six months thereafter) assessing the following characteristics: ulceration, morphology of fibrosis on T2W-MRI (regular/irregular/layered), and patterns of high diffusion signal on diffusion-weighted imaging (DWI) (non/spots/linear/mass-like).
**Results:** Hundred nine MRI scans (median 3 per patient, range 1–7) were analysed. Ulcers were observed in 11%, 40%, 44%, and 46% at 3, 6, 9, and 12 months of follow-up. Morphology of fibrosis evolved from predominantly irregular after 3 months (43% versus 25% regular) to predominantly regular after 12 months (65% versus 27% irregular). Different DWI signal patterns were observed on the first post-CXB scan; no DWI signal (25%), small spots of diffusion restriction (21%), linear signal (32%), and focal mass-like diffusion restriction (21%). Additionally, in 61% of patients, reactive high diffusion signal was observed at 3 months, which decreased later during follow-up.
**Conclusion:** MRI characteristics after CXB differ from those typically observed after conventional (chemo)radiotherapy. Findings observed early after CXB include a more irregular fibrosis and diffuse (reactive) signal changes in DWI that tend to disappear during further follow-up. These preliminary results can help radiologists to interpret MRI scans following CXB. Future research should focus on correlating imaging findings with clinical outcomes.

**14:30 - 16:00** **Auditorium 3 + 4**

*Scientific Session Live SS 4*

### Hepatocellular carcinoma detection

### SS 4.1

### Noninvasive diagnosis of small HCC: can imaging hallmarks be extracted from different imaging modalities?

A. Chouard[1], A. Paisant[2], V. Vilgrain[1], C. Aubé[2], M. Ronot[1]; [1]*Clichy/FR*, [2]*Angers/FR*

**Purpose:** To assess if nonrim arterial phase hyperenhancement [APHE] and washout can be extracted from different imaging modalities and combined for the noninvasive diagnosis of HCC on cirrhosis.
**Material and Methods:** Ancillary analysis of two prospective multicentric studies including cirrhotic patients with up-to-three 10–30mm nodules undergoing contrast-enhanced CT, extracellular agent-enhanced MRI (ECA-MRI) within one month was performed. A subset also underwent gadoxetic-enhanced MRI (GA-MRI). The reference was a composite algorithm including pathology and noninvasive tests. Noninvasive diagnosis was performed as per the two-step EASLv2018 criteria. Nodules showing both APHE and washout on either the first- or the second-line imaging exam were considered as HCC. Among the remaining nodules, those strictly displaying each hallmark on two different imaging modalities were considered.
**Results:** Total of 534 nodules in 396 patients (326 [82.3%] male; mean 62.8±9.4 years) were considered. Of them, 516 were visible on CT, 534 on ECA-MRI and 218 on GA-MRI. After applying the two-step EASLv2018 criteria, 29.5–40.8% of nodules did not meet the HCC criteria depending on the combination of imaging modalities (e.g. CT then ECA-MRI, or GA-MRI then CT, etc.). Among them, only 12 (three HCC, 25%), three (two HCC, 67%), and five (three HCC, 60%) strictly displayed each hallmark on two different imaging modalities (CT then ECA-MRI, CT then GA-MRI, and ECA-MRI then GA-MRI, respectively).
**Conclusion:** After applying the two-step EASLv2018 criteria, very few nodules display imaging hallmarks of HCC on different imaging modalities. Extracting hallmarks from different imaging modalities results in positive predictive values ranging between 25% and 67% for the diagnosis of HCC, and should, therefore, be discouraged.

### SS 4.2

### Detection of early-stage HCC with abbreviated gadoxetate MRI: comparison with ultrasound -pilot study

N. Vietti Violi[1], M. Wagner[2], C. Besa[3], M. Hulkower[4], M. Schwartz[4], S. Lewis[4], K. Sigel[4], B. Taouli[4]; [1]*Lausanne/CH*, [2]*Paris/FR*, [3]*Santiago/CL*, [4]*New York, NY/US*

**Purpose:** To compare the diagnostic performance of AMRI to US for the detection of early-stage HCC in an at-risk population.
**Material and Methods:** This prospective study included 25 patients with cirrhosis (M/F:19/6, mean age: 59y), among them 15 patients had small HCC (<3 cm) diagnosed on recent cross-sectional imaging. US and gadoxetate AMRI were performed on the same day. AMRI included T2wi+DWI+T1wi at the hepatobiliary phase. No dynamic imaging was performed. US results were based on the clinical read. AMRI was read by 2 independent observers. Images were scored according to adapted LI-RADS scoring system: negative/subthreshold/positive. Reference standard was based on routine contrast-enhanced MRI performed within 1 month. Diagnostic performance of US and AMRI was calculated, without/with AFP.
**Results:** 15 patients presented early-stage HCC (mean size: 16mm, range: 10–25mm), while 10 had no HCC. Sensitivities were 54.5% for AFP alone, 60% for US and 86.7% for AMRI (pooled data), with no significant difference. Specificities were 100% for AFP, 90% for US and 80% for AMRI, with no significant difference. When combining US and AFP, sensitivity was 81.8% and specificity 87.5%, significantly higher compared to US alone (p=0.01). When combining AMRI and AFP, sensitivity was 100% and specificity 84.6%, significantly higher when compared to AMRI alone (p=0.02).
**Conclusion:** AMRI is accurate for early-stage HCC detection. The addition of AFP to US or AMRI improved diagnostic performance for early-stage HCC detection. The study will be extended to a larger sample size to confirm these initial results and evidence a difference in diagnostic accuracy between AMRI and US for the detection of early HCC.

### SS 4.3

### Fat-containing liver lesions: are usual MRI diagnostic criteria of both malignant and benign primary liver lesions still applicable?

E. Reizine, S. Mulé, G. Amaddeo, R. Kharrat, A. Laurent, J. Calderaro, A. Luciani; *Créteil/FR*

**Purpose:** To review the MRI features of all pathologically proven fat-containing liver lesions and to evaluate the accuracy of known MRI patterns to correctly classify them.
**Material and Methods:** This was a monocentric IRB-approved retrospective study including all patients with liver lesions containing fat according to pathological report and who underwent liver MRI for characterization. After evaluation of underlying liver's morphology on MRI, the sensitivity (Se) and specificity (Sp) of different MRI patterns were evaluated focusing on patients without dysmorphic liver features.
**Results:** Between March 2014 and November 2021, 67 lesions were finally included, corresponding to 26 HCC (26/67), 33 HCA (33/67), including 12 HHCA (12/33), 15 IHCA, (15/33) 2 BIHCA (2/33) and 4 UHCA (3/33); 6 FNH (5/67) and 2 hepatic angiomyolipoma (HAML) (2/67). All lesions developed on a dysmorphic liver were HCC. For non-dysmorphic liver, an arterial phase hyperenhancement with a washout and a delayed capsule still had a 100% Se and a 98% Sp for HCC diagnosis. A homogeneous dropout of signal on the opposed phase had a Se of 92% and a Sp of 89% for the diagnosis of HHCA. The Sp increased to 100% using the combination of a typical hypovascular pattern with reduced 75% Se. The FNH pattern was also specific at 100% for the diagnosis of FNH with a reduced 40% Se. Finally, the accuracy of IHCA pattern had a low 60% Se but a high 89% Sp for IHCA diagnosis.
**Conclusion:** Known MRI patterns remain reliable for accurate diagnosis of primary liver tumors even in fat-containing lesions.

### SS 4.4

### Influence of the imaging modality of detection of hepatic nodules on the noninvasive diagnosis of HCC in cirrhotic patients

A. Paisant[1], M. Roux[1], C. Cassinotto[2], C.M. Canivet[1], O. Sutter[1], V. Vilgrain[3], C. Aubé[1]; [1]*Angers/FR*, [2]*Castelnau-le-Lez/FR*, [3]*Clichy/FR*

**Purpose:** The aim of this study was to evaluate the influence of the imaging modality of detection (i.e. nodules initially depicted by US, CT, or MRI) on the performance of the v2018 EASL algorithm to diagnose HCC on cirrhosis.
**Material and Methods:** The study was prospective and inclusions were made in eight centers. Inclusion criteria were patient with cirrhosis undergoing surveillance or follow-up with up to three liver lesions identified by US, CT, or MRI (named "initial imaging modality"). After inclusion, all patients underwent CEUS, CT, and MRI within a month (the imaging modality of detection was repeated).
**Results:** 354 HCC and 250 non-HCC were analyzed in 422 patients (mean age, 61 years ± 9.3; 350 men). US was the initial imaging modality for 267 nodules, CT for 148 nodules and MRI for 189 nodules. The initial imaging modality had no influence on the diagnostic accuracy of CT (76.4%, 77.0% and 74.6% (p=0.76)) or MRI (74.5%, 79.7% and 79.9% (p=0.24)) to diagnose HCC. No influence on the EASL two-step algorithm was observed either (p=0.283).
**Conclusion:** The modality that initially depicts a hepatic nodule does not influence the overall accuracy of the v2018 EASL algorithm to diagnose HCC. The algorithm remains valid if nodules are initially depicted by CT or MRI rather than ultrasound.

### SS 4.5

### Adherence to LI-RADS and EASL high-risk population criteria: a systematic review

R. Cannella[1], M. Dioguardi Burgio[2], R. Sartoris[3], V. Vilgrain[3], M. Ronot[3]; [1]*Palermo/IT,* [2]*Paris/FR,* [3]*Clichy/FR*

**Purpose:** LI-RADS and The European Association for the Study of the Liver (EASL) criteria for the noninvasive diagnosis of HCC can be applied in patients at high risk of HCC only. This systematic review aims to evaluate the adherence to LI-RADS and EASL high-risk population criteria in currently published research study.

**Material and Methods:** PubMed was searched from January 2012 till December 2021 for original researches reporting on performance of LI-RADS and EASL criteria for CEUS, CT, or MRI. The algorithm version, year of publication, patients' risk status and etiology of chronic liver disease were collected. Adherence and reporting of high-risk population criteria were evaluated as optimal (strict and verifiable for all patients), suboptimal (equivocal or not verifiable for all patients) or inadequate (clear violation) and compared using the Pearson $\chi^2$ test.

**Results:** A total of 222 original studies (44,885 patients) were included of which 218 assessed the LI-RADS, four the EASL, and 15 evaluated both criteria. Optimal, suboptimal, or inadequate adherence and reporting of high-risk population criteria were observed in 111/218 (50.9%), 87/218 (39.9%), 20/218 (9.2%) LI-RADS and 6/19 (31.6%), 5/19 (26.3%), 8/19 (42.1%) EASL studies (P<0.001), respectively. Adherence to high-risk population criteria significantly improved according to CT/MRI LI-RADS versions (optimal v2013.1 33.3%; v2014 24.4%; v2017 45.8%; v2018 62.8% of studies, P<0.001) and publication year (2014–2017 39.3%; 2018–2019 33.3%; 2020–2021 62.6%; P=0.002). No significant differences were between CEUS LI-RADS (P=0.388) and EASL versions (P=0.293).

**Conclusion:** Adherence and reporting of high-risk population criteria are optimal only in half of the published studies, but a significant improvement is observed with the latest LI-RADS version and in more recent publications.

### SS 4.6

### Fat hepatocellular carcinoma in patients with cirrhosis: a new diagnostic algorithm proposal

A. Delagnes[1], M. Roux[1], V. Vilgrain[2], B. Guiu[3], V. Laurent[4], O. Sutter[5], I. Bricault[6], H. Trillaud[7], C. Aubé[1], A. Paisant[1]; [1]*Angers/FR,* [2]*Clichy/FR,* [3]*Montpellier/FR,* [4]*Vandoeuvre-lès-Nancy/FR,* [5]*Bobigny/FR,* [6]*Grenoble/FR,* [7]*Bordeaux/FR*

**Purpose:** Non-invasive diagnosis of HCC can be made in cirrhotic patients by MR based on defined hallmarks. Fat in MR inside the HCC limits its non-invasive diagnosis and biopsy is often necessary. The aim of this study was to describe the characteristics of these HCCs with fat in MR. The second aim was to define new MR criteria for the non-invasive diagnosis of fat HCC and to propose a related diagnosis algorithm.

**Material and Methods:** This ancillary study of a prospective multi-centric study included 84 patients with 77 fat HCCs and 11 non-HCC fat nodules in cirrhosis. All MR were reviewed with nodule characteristics on each sequence, as well as The European Association for the Study of the Liver (EASL) and LiRADS classification. The follow-up was at least 3 years.

**Results:** Fat HCCs presented arterial phase hyperenhancement (APHE) in 54 patients (70.1%), arterial phase vascularization (enhancement in the arterial phase compared to the nodule itself on T1 with unenhancement) in 62 (80.5%), washout in 43 (55.8%) and capsule in 20 (26.0%). For the diagnosis of fat HCC, EASL and LiRADS have a sensitivity of 37.7% and 36.4%, respectively, and a specificity of 100%. A new suggested fat-LiRADS algorithm, including a broadening of the APHE definition, increased sensitivity to 50.6%, with a specificity of 100%. Overall survival was at 1 year and at 3 years 87.2% and 67.2%, respectively; recurrence-free survival was 83.9% and 48.9%, respectively.

**Conclusion:** This new algorithm for the non-invasive diagnosis of fat HCC on cirrhosis has a higher sensitivity than the classic LiRADS, without degrading specificity.

### SS 4.7

### Positive predictive value of hepatocellular carcinoma surveillance ultrasound depends on the presence of cirrhosis

J. Tse, L. Shen, T. Liang, L. Yoon, A. Kamaya; *Stanford, CA/US*

**Purpose:** To determine how cirrhosis affects positive predictive values (PPV) of LI-RADS US-3 observations.

**Material and Methods:** In this retrospective study, 225 adult patients (100 women, 125 men; 59±14 years) high risk for HCC from 2017 to 2021 had an US-3 observation on surveillance ultrasound and underwent diagnostic testing with CT (93; 41%), MRI (130; 58%), or contrast-enhanced ultrasound (2; 1%). US-3 observations included focal observations ≥10 mm in 217 patients and venous thrombi in 8 patients. PPV were calculated using diagnostic testing as the reference standard.

**Results:** Of 225 patients, 116 (52%) had cirrhosis and 109 (48%) did not. Most non-cirrhotic patients had chronic hepatitis B (n=100). At diagnostic testing, a correlate was identified in 154 patients (68%) with the following distributions: 74 LR-1 or LR-2 (including 52 hemangiomas), 41 LR-3, 28 LR-4 or LR-5, 5 LR-M, 1 LR-TIV, and 5 bland thrombi. Among cirrhotic patients, the PPV for LR-3 or higher was 51% (95% confidence interval 42–60%) and LR-4 or higher was 26% (18–34%). Among non-cirrhotic patients, the PPV decreased to 15% (8–21%; p<0.001) for LR-3 or higher and 4% (0–7%; p<0.001) for LR-4 or higher. At multivariable analysis, cirrhosis was the most important predictor of LR-4 or higher (p<0.001; odds ratio OR 20.4), followed by ultrasound observation size (p<0.001; OR 2.65) and age (p=0.004; OR 1.05). Alpha-fetoprotein and modality (CT vs MRI) were not significant predictors.

**Conclusion:** PPV for LR-4 or higher (probable or definite HCC) in US-3 observations decreases from about 1 in 4 among cirrhotic patients to 1 in 25 among non-cirrhotic patients.

### SS 4.8

### Impact of COVID-19 pandemic on hepatocellular carcinoma ultrasound surveillance volumes at a liver transplant center

J. Tse, L. Shen, L. Yoon, A. Kamaya; *Stanford, CA/US*

**Purpose:** The COVID-19 pandemic led to nationwide postponement of outpatient preventative health services. The purpose of our study was to determine the effect of the COVID-19 pandemic on hepatocellular carcinoma ultrasound (HCC US) surveillance volumes at a liver transplant center.

**Material and Methods:** HCC US surveillance volumes were recorded 12 months prior to the pandemic (March 1, 2019, to February 29, 2020) to serve as baseline comparison to the months following the start of the pandemic (March 1, 2020–December 31, 2021). HCC US findings and recommendations for additional imaging were extracted from the report.

**Results:** From March 1, 2019–February 29, 2020, a total of 2,468 HCC US surveillance examinations were performed (206±21 per month; mean±standard deviation). This decreased to 1,835 in the first pandemic year from March 1, 2020, to February 28, 2021 (153±58 per month; p=0.007) and 1870 in the first 10 months of the second pandemic year from March 1, 2021, to December 31, 2021 (187±22 per month; p=0.042). April 2020 had the sharpest decline in HCC US surveillance examinations, with monthly volume 2% of baseline. Cumulative HCC US surveillance examinations with an US-2 (focal observation ≤10 mm) or US-3 finding (focal observation ≥10 mm or new venous thrombus) decreased from 274 (baseline) to 183 (first pandemic year) to 169 (10 months of second pandemic year).

**Conclusion:** HCC US surveillance volumes and cumulative number of observations requiring additional workup decreased during the COVID-19 pandemic. Despite gradual re-opening of outpatient preventative health services, volumes are still below that of pre-pandemic.

### SS 4.9

### Low contrast agent and radiation dose liver CT with deep-learning denoising model in participants at high risk for HCC: prospective, randomized, single-blinded preliminary study

H.-J. Kang, J.M. Lee, J.H. Kim, S. Han; *Seoul/KR*

**Purpose:** To investigate image quality and clinical feasibility of the simultaneous reduction of contrast doses and radiation at liver CT with deep-learning denoising (DLD) algorithm in participants at high risk for HCC.

**Material and Methods:** In this prospective study, participants at high risk of HCC were consecutively recruited and underwent liver CT. They were assigned to either the standard-dose (standard-dose group) or the double-low dose acquisition (double-low dose group), and further processed by DLD (double-low dose with DLD group). The contrast-to-noise ratio (CNR), qualitative image quality, and per-lesion sensitivity of HCC were compared among groups by t test or ANOVA. Lesion detectability was also compared by Jackknife alternative free-response receiver operating characteristic curve analysis.

**Results:** Sixty-eight participants with 57 focal liver lesions (FLL) were evaluated (20 HCCs, 37 benign). The CTDIvol (8.6±2.2 mGy vs. 6.9±0.6 mGy, P<.001) and a dose of contrast agent (106.9±15.0 mL vs. 77.9±9.4 mL, P<.01) were higher in the standard-dose group than the double low-dose group. The comparative analysis demonstrated that the CNR (P<.01) and vessel conspicuity (P<.01) were significantly higher on DLD in double-low group than standard-dose group. There was no significant difference in lesion detectability between the three groups (P=0.74). The per-lesion sensitivity of an HCC was lower in double-low dose group than standard-dose group (P=0.045), whereas there was no significant difference between standard-dose and double-low dose with DLD group (P=0.25).

**Conclusion:** The DLD provided improved image quality without significant impairment of per-lesion sensitivity of an HCC than standard-dose acquisition, even with simultaneous reduction of radiation and contrast doses in participants with high risk for HCC.

### SS 4.10

### Determining the radiological outcome of subcentimeter-sized hepatic nodules demonstrating non-rim arterial phase hyperenhancement in at-risk patients under surveillance for hepatocellular carcinoma in the Indian population

D. Mehta[1], D. Lokhandwala[1], S. Sabnis[1], S. Siddiqui[1], S. Nellore[2], S. Jalkote[1], K. Ganesan[1]; [1]*Mumbai/IN,* [2]*Navi Mumbai/IN*

**Purpose:** To evaluate the interval growth and outcome of subcentimeter-sized observations (SCSOs) demonstrating non-rim like arterial phase hyperenhancement (APHE) detected during surveillance for HCC in at-risk livers using LIRADS in the Indian population.

**Material and Methods:** This prospective observational study included 74 patients with at-risk livers, serially evaluated between October 2014 and April 2021, for SCSOs demonstrating non-rim APHE using two contrast-enhanced cross-sectional (CECT/CEMRI) imaging studies performed at 3–6-month interval. Statistical analysis was performed using t test or Mann–Whitney U test for continuous data, and categorical variables were compared using the chi-square test or Fisher's exact test.

**Results:** We prospectively evaluated 115 SCSOs demonstrating non-rim like APHE. The results predicted a 23% probability of developing HCC on a per-person basis and 15.7% probability on a per-observation basis. Nine of these observations were upscaled to a higher LIRADS category without definitive development of malignancy. Patient age, gender, initial size of observation, initial serum AFP levels and etiology of liver disease were not found to have any significant statistical difference in the HCC and non-HCC groups.

**Conclusion:** Early detection of HCC is vital. Precise characterization of SCSOs is delayed due to the necessity of follow-up imaging to assess interval growth or stability. Interval threshold growth represents an important surrogate marker for malignant transformation of subcentimeter size observations in at-risk liver. The overall incidence of HCC developing in a SCSO in our Indian study population was comparable to the results of previous studies.

**09:00 - 10:30** **Auditorium 3 + 4**

*Scientific Session Live SS 5*
### Interventional radiology

### SS 5.2
### Radiofrequency ablation of HCC: CT texture analysis of the ablated area to predict local recurrence

D. Autelitano, L. Geraci, L. Tomaiuolo, C. Longo, M. Todesco, A. Carli, R. De Robertis, M. D'Onofrio; *Verona/IT*

**Purpose:** To investigate the role of CT texture analysis in the risk assessment of local recurrence after HCC ablation.
**Material and Methods:** Patients treated with percutaneous liver ablation were retrospectively enrolled between January 2015 and December 2018. CT texture analysis was performed both on the core and borders of the ablation area 1–2 months after procedure. Tumors were grouped according to the onset of local recurrence at follow-up (persistence, recurrence-free, short- or long-term recurrence). ANOVA/Kruskal–Wallis tests and a multivariable Cox regression model were used to assess differences in texture parameters and which parameters were predictive of recurrence risk.
**Results:** 263 tumors were treated in 200 patients; 98 patients (72±9 years, 83 men) met the inclusion criteria for a total of 151 HCCs considered independently. 68 HCCs reported no disease recurrence, 32 persistent disease, 19 short-term and 32 long-term recurrence. Median follow-up was 121 [range: 29–1680] days. VenSkewness (p=0.02) and VenKurtosis (p=0.01) of the ablation core were predictive of short-term recurrence. VenHUmean (p=0.02) and VenGLRLM_HGRE (p=0.02) of the core were independent predictors of tumor recurrence. ArtEntropy of ablation border predicted the recurrence risk (p=0.04) and values higher than 3.71 reported an increased recurrence incidence (p=0.05). ArtHUstd (p=0.01), LateHUmean (p=0.04), LateGLRLM_HGRE (p=.003), LateGLZLM_HGZE (p<0.01) and LateGLZLM_SZHGE (p=.02) of ablation border were independent predictors of local recurrence risk.
**Conclusion:** CT texture analysis of the ablation area performed at 1–2-month follow-up allows to estimate the risk of local recurrence of HCC treated by radiofrequency ablation; one may be able to modify clinical–therapeutic decisions accordingly.

### SS 5.3
### Prospective assessment of liver health following liver resection and yttrium-90 radioembolization in patients with hepatocellular carcinoma using multi-parametric MRI platform

M. Pansini[1], J. Connell[1], A. Fichera[1], L. Nuñez[1], E. Pickels[1], D.C. Ng[2], A. Gogna[2], B. Goh[2], D. Wai Meng Tai[2], H. Chong Toh[2], S.P. Choo[2], W.Y. Chan[2], T. Priyanthi[2], I.B. Tan[2], R.H. Lo[2], S.X. Yan[2], C. Chua[2], S.L. Koo[2], K.H. Lim[2], J.A. Latiff[2], H.S. Chua[2], T.H. Choon[2], R. Banerjee[2], M. Brady[1], M. Kelly[1], P. Chow[2]; [1]*Oxford/UK,* [2]*Singapore/SG*

**Purpose:** For unresectable HCC, AASLD guidelines recommend locoregional therapy such as transarterial chemoembolization (TACE). Yttrium (Y-90) radioembolization as locoregional treatment for HCC is becoming a more frequent approach to treat unresectable tumours. However, the effect on the surrounding healthy parenchyma is poorly understood and not well monitored. In this study, we assess the use of corrected T1(cT1) as biomarker of liver fibro-inflammation and the efficacy of multi-parametric MRI to monitor liver health in patients affected by HCC.
**Material and Methods:** This investigator-initiated pilot study (RCT: NCT04451603) consisted of 13 participants diagnosed with HCC; 7 received selective internal radiotherapy (SIRT) with Y-90 in one or both lobes; the remaining 6 had a surgical resection. All patients have been monitored with multi-parametric MRI before and sequentially after undergoing therapy (1 and 3 months post-treatment). Multi-parametric MRI allowed quantification of liver fibro-inflammation and liver fat, and calculation of total liver volume and relative change in treated and untreated lobes, before and after the treatment.
**Results:** A significant reduction in cT1 was observed in the liver of patients 3 months after radioembolization with Y-90 (p=0.007). Two of the three patients who received single-lobe radioembolization showed signs of contralateral hypertrophy, with an increased volume of ~14% in the untreated lobe. Patients undergoing surgical resection showed a range of liver fibro-inflammation responses.
**Conclusion:** Multi-parametric MRI monitoring enables a personalized assessment of the effects of HCC treatment on liver health. Our results indicate the region of the liver undergoing contralateral hypertrophy is of good health, based on regional cT1.

### SS 5.4
### Safety and efficacy of hepatic intra-arterial treatments in patients contraindicated for hepatectomy after portal vein embolization

R. Bacquet[1], M. Dioguardi Burgio[2], L. Raynaud[1], L. Paulatto[3], A. Sibert[1], L. Garzelli[1], J. Gregory[1], J.-C. Bijot[1], A. Chouard[1], V. Vilgrain[1], M. Ronot[1]; [1]*Clichy/FR,* [2]*Paris/FR,* [3]*Montreuil/FR*

**Purpose:** To assess the safety and efficacy of hepatic intra-arterial therapy (IAT, i.e. transarterial chemoembolization [TACE] and radioembolizationin [TARE]) in patients who underwent portal vein embolization (PVE) before major hepatectomy but were subsequently contraindicated for surgery.
**Material and Methods:** Patients who underwent PVE from 2007 to 2019 and were subsequently contraindicated for surgery were identified. Among them, those who underwent IAT were included. Adverse events (AE) were graded according to the SIR classification. The tumor response rate was assessed with RECIST 1.1. Overall (OS) and progression-free survival (PFS) were estimated by plotting Kaplan–Meier curves.
**Results:** One hundred ten of 383 patients (28%) who received PVE did not undergo surgery. Of these, 15 (14%) patients (12 men, median 61 years old [IQR 56–67]) underwent 37 IAT procedures (26 TACE and 11 TARE). Most patients (n=11/15, 73%) had hepatocellular carcinoma. Nine AE occurred in six patients after IAT (seven minor, two major). Best response according to RECIST 1.1 was PR, SD, and PD in four (27%), 10 (66%), and one patient (7%), respectively. Patients were followed for a median of 37 months (IQR 17–44). Eleven of 15 patients (73%) showed progression and 8 (53%) had died at the time of data collection. The median estimated OS was 42 months (CI 95% 35–49 months), and the median estimated PFS was 33 months (CI 95% 24–42 months). There was no difference in OS between patients treated with TACE and TARE (p = 0.33).
**Conclusion:** Hepatic IAT can be safely performed in patients contraindicated for liver surgery after PVE, with acceptable efficacy.

### SS 5.6

### Percutaneous management of benign biliary strictures after liver transplantation in pediatric patients: a single-center experience

L. Dulcetta[1], P. Marra[1], F. Carbone[1], R. Muglia[1], P.A. Bonaffini[1], S. Sironi[2]; *[1]Bergamo/IT, [2]Milan/IT*

**Purpose:** To assess the role of percutaneous transhepatic cholangiography (PTC), transluminal bilioplasty, and internal–external biliary drainage (IEBD) placement for diagnostic confirmation and treatment of benign biliary strictures (BBSs) in pediatric orthotopic liver transplantation (OLT). To evaluate technical success and short-term clinical outcome.
**Material and Methods:** Clinical, laboratory, imaging and procedural data of 52 pediatric patients (median age = 10.5 months; IQR = 15; 28 males), who underwent PTC after OLT between 2009 and 2020 in a single center, were analysed. Indication to PTC was evidence of cholestasis at laboratory data or at liver biopsy, otherwise bile duct dilation at pre-procedural imaging.
**Results:** A total of 122 PTCs were performed (median procedures per patient = 2; IQR = 2) and all were technically successful. In 108/122 (88.5%) cases, BBS was found and treated at PTC. In 11/122 (9%) cases, radiological management was not possible and patients underwent retransplantation. In 3/122 (2.5%), there was no evidence of stenosis at PTC. Overall, IEBD was maintained for a median of 31 days (IQR = 54.5). Procedure-related complications were 35 (28.9%) cholangitis, 4 (3.3%) bilomas, 3 (2.5%) sepsis, 1 (0.8%) fatal septic shock and 1 (0.8%) melena. Reduction in cholestasis after biliary interventions was indicated by significantly improving in serum levels of γ-glutamyl transpeptidase ($p < .001$), total bilirubin ($p < .005$), direct bilirubin ($p < .05$) and alkaline phosphatase ($p < .05$).
**Conclusion:** In pediatric OLT, PTC with bilioplasty and IEBD placement are technically feasible, have acceptable complications rate and improve cholestasis by short term.

### SS 5.7

### To retrospectively report 10-year experience in diagnostic and interventional imaging versus conservative management of pediatric acquired arterioportal fistulas

B. Di Fazio, P. Marra, F. Carbone, L. Dulcetta, P.A. Bonaffini, S. Sironi; *Bergamo/IT*

**Purpose:** To retrospectively report 10-year experience in diagnostic and interventional imaging versus conservative management of pediatric acquired arterioportal fistulas (APFs).
**Material and Methods:** Post-orthotopic liver transplantation (OLT) pediatric patients with diagnosed arterioportal shunt by color Doppler ultrasound (CDUS) or computed tomography angiography (CTA) were finally included. CTA was performed only when CDUS was not conclusive and for urgent clinical conditions. APFs were presumed as iatrogenic when first detected after percutaneous liver procedures. Interventional versus conservative approach was chosen on imaging findings (hemodynamic effect on portal vein flow at CDUS), emergent or elective status, APF morphology and location.
**Results:** 23 pediatric patients were selected with 24 imaging-proven diagnoses of APF. Eleven underwent at least one attempt of IR treatment; twelve were conservatively managed. Primary or secondary technical success was obtained in 12/16 procedures (75%). Technical failure was attributed to various reasons: hepatic artery dissection, lack of exact localization of the shunt, anatomical complexity of the shunt and portal thrombosis. Five (22%) patients underwent reinterventions. Clinical success was achieved in 8/11 (73%) patients, with improvement of CDUS findings. One patient died of multiorgan failure 1 month after angiography without embolization. Two patients with shunt persistence required OLT. Complications without sequelae occurred in 17% of procedures. Conservatively treated patients never required other intervention during follow-up, most with stable CDUS findings.
**Conclusion:** Early treatment of APFs is required in pediatrics although uniform technique for APF percutaneous embolization cannot be established because of multiple features. CDUS is a reliable tool to guide the management.

### SS 5.8

### Percutaneous stenting of malignant biliary hilar obstructions using the moving cell stent: a single-center experience

F. Cortese, F. Acquafredda, A. Mardighian, M.T. Zurlo, M. Conticchio, R. Memeo, R. Inchingolo; *Acquaviva delle Fonti/IT*

**Purpose:** Percutaneous bilateral biliary stenting is an established method for the management of hilar strictures. The aim of this study was to evaluate the safety and efficacy of a novel uncovered biliary stent, specifically designed for hilar reconstruction.
**Material and Methods:** This single-center, observational study included consecutive patients undergoing percutaneous transhepatic moving cell stent placement for hilar reconstruction using the stent-in-stent technique for malignant biliary strictures, between November 2020 and July 2021. Primary endpoints were technical (appropriate stent placement) and clinical (relief from jaundice) success. Secondary endpoints included stent patency, overall survival, complication rates and dysfunction stent.
**Results:** In total, 18 patients (mean age 71 ± 11 years; 61.1% male) were enrolled, suffering from cholangiocarcinoma (12/18; 66.6%), gallbladder cancer (5/18; 27.7%), and colorectal liver metastasis (1/18; 5.5%). The technical and clinical success rate was 100% (18/18 cases). According to Kaplan–Meier analysis, the estimated overall patient survival was 80.5% and 60.4% at 6 and 12 months, respectively, while stent patency was 90.9% and 68.2% at 6 and 12 months, respectively. The median stent patency was 172,5,3±58,8 days. A single periprocedural complication was reported. Stent dysfunction was observed in 5 patients (27.7%), including 1 occlusion stent (5.5%) and 1 migration stent.
**Conclusion:** The moving cell stent resulted in excellent technical and clinical success rates following hilar bifurcation biliary stenting, with acceptable complication rates. Further research is required to confirm this initial positive experience.

### SS 5.9

### Analysis of postpancreatectomy hemorrhage outcomes after interventional radiology treatment

F. Mambrin, H. Nguyen, C. Gasparini, A. Contro, G. Mansueto, R. De Robertis Lombardi; *Verona/IT*

**Purpose:** Postpancreatectomy hemorrhage (PPH) is a feared complication of pancreatic surgery. According to ISGPS PPH classification, interventional radiology (IR) is indicated to treat grade B or C PPH. In our institution, a worse outcome was observed in patients with central PPH (cPPH) than in patients with peripheral PPH (pPPH). The purpose of this study is to investigate whether a significant difference between the two groups exists, to define predictors of PPH severity.
**Material and Methods:** Patients who reported grade B or C PPH treated with IR between 2008 and 2021 were included (n=39). Patients were divided into two groups based upon the distance between the aortic wall and the bleeding onset point: distance ≤2 cm (cPPH) or >2 cm (pPPH). Type of endovascular treatment (embolization or stent-graft placement), hospital-acquired infections (HAI) onset and expiration occurrence were logged.
**Results:** cPPH was more commonly treated with stent-grafts (69%) than pPPH (29%), $p=0.020$. Further surgical or IR treatments were required in 54% of cPPH and in 38% of pPPH, $p=0.360$. cPPH reported a worse outcome than pPPH group, with death occurrence of 31% and 23%, respectively, $p=0.710$. When no complication occurred, PPH recovery was 100%; however, when HAI complicated PPH recovery, 43% of patients deceased during hospitalization, $p=0.002$. When PPH was complicated by HAI, 31% of cPPH patients expired versus 23% of pPPH ($p=0.650$).
**Conclusion:** Stent-graft placement is a viable option for cPPH treatment. The distance of bleeding onset from the aorta can be used as a predictor of PPH severity. HAI strongly impact the outcome of PPH, especially of cPPH.

### *SS 5.10*

### Completeness of reporting in abstracts of published liver interventional radiology randomized controlled trials

J. Gregory[1], I. Boutron[2], V. Vilgrain[1], M. Ronot[1]; [1]*Clichy/FR,* [2]*Paris/FR*

**Purpose:** An extension to the CONsolidated Standards Of Reporting Trials (CONSORT) statement for abstracts was published in 2015 to ensure quality of reporting for non-pharmacological treatments (NPT). Our aim was to assess the completeness of reporting of abstracts of published randomized controlled trials (RCTs) for liver interventional radiology.

**Material and Methods:** MEDLINE and EMBASE were searched for liver interventional radiology RCTs (January 2015–September 2020). Two reviewers checked whether the 32 items of the CONSORT-NPT were adequately reported. Additional information included date of publication, number of centers, university-affiliated center, phase of the RCT, funding, journal specialty, open access, number of authors, authors' gender, number of words, and number of keywords.

**Results:** In total, 107 abstracts of RCTs published in 61 journals were included. Overall, 13/61 journals (21%) endorsed the CONSORT-NPT for abstract. The median number (Q1-Q3) of items adequately reported was 14 (11–16.5). Overall, 32% (34/107) and 7% (8/107) of abstracts reported ≥50%, and ≥75% of items. The three items specific to NPT were reported in nine (8%, eligibility of care provider), five (5%, attempt for intervention standardization) and 36 abstracts (34%, significant deviation from planned intervention). The publication of the CONSORT-NPT for abstract improved reporting in non-open access journals (*P*<0.01), single-center RCTs (*P*<0.01), and for RCTs with non-corporate authors (*P*<0.01). All other factors had no influence. There was a correlation between the quality of reported and the number of words in abstracts (r=0.57).

**Conclusion:** Despite improvement over time, completeness of reporting is poor in abstracts of RCT for liver interventional radiology. Items specifically dedicated to describing interventions are particularly overlooked.

**09:00 - 10:30** **Auditorium 8**

### *Scientific Session Live SS 6*
### Bowel inflammation

### *SS 6.1*

### Diagnostic performance of magnetic resonance enterography disease activity indices compared with a histological reference standard for adult Crohn's disease: experience from the METRIC trial

S. Kumar, T. Parry, S. Mallett, G. Bhatnagar, A. Plumb, S. Halligan, S. Taylor, . METRIC study Investigators; *London/UK*

**Purpose:** The simplified magnetic resonance enterography (MRE) index (sMARIA), London and "extended" London scoring systems are widely used in Crohn's disease (CD) to assess disease activity, although validation studies have usually been single centre, retrospective and/or used few readers. Here, we evaluated these MRE indices within a prospective multicentre, multireader diagnostic accuracy trial.

**Material and Methods:** A subset of participants (newly diagnosed or suspected of relapse) recruited to the METRIC trial with available terminal ileal (TI) biopsies was included. Using pre-specified thresholds, the sensitivity and specificity of sMARIA, London and "extended" London scores for active and severe (sMARIA) CD were calculated using different thresholds for the histological activity index (HAI).

**Results:** We studied 111 patients (median 29 years, interquartile range 21-41, 75 newly diagnosed, 36 suspected relapse) from 7 centres, of whom 22 had no active TI CD (HAI=0), 39 mild (HAI=1), 13 moderate (HAI=2), and 37 severe CD activity (HAI=3). In total, 26 radiologists prospectively scored MRE datasets as per their usual clinical practice. Sensitivity and specificity for active disease (HAI >0) were 83% (95% confidence interval 74–90%) and 41% (23–61%) for sMARIA, 76% (67–84%) and 64% (43–80%) for the London score, and 81% (72–88%) and 41% (23–61%) for the "extended" London score, respectively. The sMARIA had 84% (69–92%) sensitivity and 53% (41–64%) specificity for severe CD.

**Conclusion:** When tested at their proposed cut-offs in a real-world setting, sMARIA, London and "extended" London indices achieve high sensitivity for active disease against a histological reference standard, but specificity is low.

### SS 6.2

### Diagnostic performance of apparent diffusion coefficient to assess therapeutic response to biological therapy in Crohn's disease

J. Rimola[1], N. Capozzi[2], A. Fernandez-Clotet[1], S. Rodriguez[1], B. Caballol[1], I. Ordás[1], J. Panes[1]; [1]*Barcelona/ES,* [2]*Bologna/IT*

**Purpose:** To determine prospectively the performance of apparent diffusion coefficient (ADC) to assess response to biological therapy in Crohn's disease (CD) using magnetic resonance enterography (MRE) and ileocolonoscopy as reference standards.

**Material and Methods:** 74 CD patients underwent MRE at baseline and at 46 weeks after treatment with biological drugs. MRE images were analysed including measurement of ADC. Segmental MaRIA was considered the reference standard to identify presence and severity of inflammation. Similar analysis was done in the 41 subjects who underwent ileocolonoscopy at both time points.

**Results:** 125 intestinal segments had severe inflammation according to MaRIA score (segmental MaRIA >11) at baseline. Using a receiver operating characteristic (ROC) curve, we determined an ADC threshold of $1.301\times10^{-3}mm^2/s$ to predict severity, yielding a sensitivity of 0.78 and specificity of 0.94 to identify segments with MaRIA >11 (AUROC=0.92). Using the same threshold, ADC showed a sensitivity and specificity of 0.75 and 0.78, respectively, to identify healing of severe inflammation (MaRIA<11). Using ileocolonoscopy as reference, the ADC threshold to predict endoscopic severity (presence of ulcers) was very similar ($1.382x10^{-3}mm^2/s$) yielding a sensitivity and specificity of 0.76 and 0.88, and 0.95 and 0.71 to identify ulcer healing. The MaRIA score was superior to ADC to predict endoscopic ulcer healing (AUROC 0.94 vs. 0.83 p=0.01).

**Conclusion:** ADC showed robust performance to predict severe inflammation in CD using either the MaRIA index or ileocolonoscopy as reference standards, and could be used as an alternative to the MaRIA index to assess therapeutic response in CD.

### SS 6.4

### MR elastography of the ileocecal mesentery in Crohn's disease

K.J. Beek, N.P. Wassenaar, A.-S. van Schelt, J. Runge, J. Stoker; *Amsterdam/NL*

**Purpose:** Creeping fat (CF) is involved in Crohn's disease (CD) pathophysiology. Ileocecal resection (ICR) with mesentery resection is associated with reduced reoperations versus ICR alone. We hypothesized that CF has a different stiffness than healthy mesentery and this could function as a biomarker for active CD (aCD). Magnetic resonance elastography (MRE) is a non-invasive MR method that measures visco-elastic properties of soft tissue. The purpose of our pilot study was to investigate differences in shear stiffness and phase-angle of the ileocecal mesentery with MRE.

**Material and Methods:** Multifrequency (30, 40, 50, 60Hz) MRE was performed at 3.0T (Ingenia, Philips) in five male patients (age 36.22; IQR 22.88–51.69) with aCD, counseled for ICR and compared with five healthy males (28.75 IQR 25.34–49.77). Within aCD patients, diseased mesentery was compared with presumably healthy mesentery at least 50cm proximal to CF. Tissue of interest was manually drawn in the mesentery at the ileocecal junction. Shear stiffness (|G*|) and phase-angle (ϕ) were calculated using MDEV inversion and analyzed.

**Results:** For |G*| and ϕ significant differences (p<0.05) were found between healthy volunteers and aCD patients; mean |G*|=578.2Pa (SD27.7) and 656.8Pa (SD39.1) and median ϕ=0.44rad (IQR 0.40–0.47) and 0.51rad (IQR 0.47–0.67), respectively. Within aCD patients, |G*| and ϕ showed no difference between CF and presumed normal mesentery, mean difference 36.4Pa (p=0.5) and Z=8.0 (p=0.9), respectively.

**Conclusion:** Based on this pilot study, we showed that CF has both a higher shear stiffness and higher phase-angle than healthy mesentery in healthy volunteers. This implies that MRE could function as a biomarker for active CD. Within aCD patients, no differences were identified.

### SS 6.5

### Crohn's disease: evaluation of intestinal fibrosis by fibroblast-activating protein inhibitor-PET-MR-enterography

M. Scharitzer, A. Beer, T. Mang, A. Haug, L.W. Unger, W. Reinisch, M. Weber, L. Nics, T. Nakuz, M. Hacker, M. Bergmann, S. Rasul; *Vienna/AT*

**Purpose:** In patients with Crohn's disease, differentiation between intestinal inflammation and fibrosis has implications on treatment. However, no imaging modality exists to specifically discriminate an inflammatory from a fibrotic component within a bowel stricture. This proof-of-concept study aims to assess the feasibility of $^{68}$Ga-labelled fibroblast-activating protein inhibitor (FAPI) PET-MR-enterography (PET-MRE) to assess bowel wall fibrosis in patients with Crohn's disease.

**Material and Methods:** In this prospective study, patients with Crohn's disease and intestinal stricture undergoing PET-MRE using [$^{68}$Ga]Ga-DATA$^{5m}$.SA.FAPI as radiotracer and subsequent surgical resection were eligible for enrollment. Based on PET-MRE findings, intestinal segments were defined for histopathological analysis within the surgical specimen, including inflamed and non-inflamed segments. The association of [$^{68}$Ga]Ga-DATA$^{5m}$.SA.FAPI PET uptake intensity (SUVmax) and histopathological presence of fibrosis by hematoxylin and eosin stain was evaluated for each segment (t test).

**Results:** 29 bowel segments with histopathological workup in seven patients were included for analysis. Fibrosis was diagnosed histopathologically in 16 segments (fibrosis grade 1, n=9; grade 2, n=7). The association between [$^{68}$Ga]Ga-DATA$^{5m}$.SA.FAPI uptake intensity and histopathological diagnosis of fibrosis was significant (p<.001) with a mean SUVmax in fibrotic segments of 9.95 (SD 5.87) and nonfibrotic segments of 2.58 (SD 1.26). No significant difference could be found between SUVmax and the histopathological grade of fibrosis (p=.592).

**Conclusion:** We observed a strong association of [$^{68}$Ga]Ga-DATA$^{5m}$.SA.FAPI uptake intensity and histopathological bowel wall fibrosis in patients with Crohn's disease, implying high diagnostic potential for treatment decision.

### SS 6.6

### Untangling and segmenting the small intestine in 3D cine-MRI using deep learning

L.D. van Harten, C.S. de Jonge, K.J. Beek, I. Išgum, J. Stoker; *Amsterdam/NL*

**Purpose:** In the absence of image analysis tools enabling investigation of the intestines as 3D structures, quantitative assessment of small intestinal motility in 3D cine-MRI is limited to the evaluation of movement in separate 2D slices. To allow extensive analysis with higher level of detail, we propose a method to automatically untangle and segment the small intestine in 3D cine-MRI.

**Material and Methods:** We used retrospectively collected 3D cine-MRI scans (Philips Ingenia 3T, spacing: 2.5x2.5x2.5mm, FOV: 400x400x35mm, 1.0 second per volume) from 14 healthy volunteers (used in method development) and 10 patients scheduled for ileocecal resection surgery (used only for evaluation). The small intestine was manually segmented and centerlines were manually annotated for intestinal segments (185 in the set of volunteers, 127 in the set of patients). The method automatically tracks and segments the small intestine in a time-point from 3D cine-MRI scans using a convolutional neural network-based stochastic tracker. The untangled representation of the intestines is constructed by resampling the image along the extracted centerlines; the automatic segmentation is produced by the network directly. The method was evaluated using cross-validation in the set of healthy volunteers, as well as by evaluating on the separate set of patients.

**Results:** Our method achieves a centerline tracking recall of 0.74±0.07 and 0.76±0.12 in the sets of volunteers and patients, respectively, a precision of 0.80±0.06 and 0.86±0.11 and a segmentation Dice coefficient of 0.88±0.03 and 0.79±0.09, respectively.

**Conclusion:** The presented method provides a major first step towards automatic detailed quantitative assessment of small intestinal motility in abdominal 3D cine-MRI.

### *SS 6.7*

### Comparative evaluation of perianal fistula by MR sequences with surgical reference

H. Özen Atalay, L. Soydan; *Istanbul/TR*

**Purpose:** We aimed to evaluate the diagnostic performance of MRI in preoperative evaluation of perianal fistula and to investigate if diffusion-weighted imaging (DWI) could be used to predict the activity of PF, using post-surgical findings as reference.
**Material and Methods:** Preoperative MR images of 42 patients who were operated because of a draining or suspected PF were retrospectively reviewed. Fat-suppressed T2-weighted imaging (FS T2WI), FS post-contrast (PC) T1WI and DWI sequences were used. With post-surgical findings taken as reference, the MR sequences and the apparent diffusion coefficient (ADC) of DWI were evaluated to determine the diagnostic performances of MRI and to investigate a correlation with PF activity, respectively.
**Results:** Overall accuracies of FS PC T1WI, DWI and FS T2WI were 93.1%, 91.4%, 83.3%, respectively. The overall accuracy of FS PC T1W was significantly higher than that of FS T2W TSE (p=0.007). The mean ADC values of active and inactive PF were (0.919 ± 0.165) x$10^{-3}$ $mm^2/s$ and (1.235 ± 0.220) x$10^{-3}$ $mm^2/s$, respectively, with a significant difference ($p < 0.0005$). A cut-off mean ADC value of 1.005 ×$10^{-3}$ mm/s to was used to differentiate active from the inactive PF with a sensitivity of 84% and specificity of 71.5%.
**Conclusion:** FS post-contrast T1WI imaging has the highest accuracy in identifying PF and its associated findings. DWI performs nearly equally efficiently and can be used in cases with contraindication to contrast. Mean ADC can differentiate active from the inactive fistulas and may predict cases with a higher likelihood of postoperative recurrence.

### *SS 6.8*

### Interobserver agreement of MAGNIFI-CD index and radiological visual analogue scale of disease activity in patients with perianal fistulizing Crohn's disease

K.J. Beek, L. Mulders, K. Horsthuis, J. Tielbeek, C. Buskens, K. Gecse, G.R. D'Haens, J. Stoker; *Amsterdam/NL*

**Purpose:** MRI is the recommended imaging modality for diagnosis, classification, and treatment monitoring in perianal fistulizing Crohn's Disease (pCD). Recently, the MAGNIFI-CD index was introduced for clinical trials. For daily clinical practice, a simple radiological visual analogue scale (rVAS) of disease activity might be a practical alternative. A requirement before MAGNIFI-CD index and rVAS can be used is substantial reliability. Our purpose was to test interobserver agreement of the MAGNIFI-CD index, its separate items and a rVAS of disease activity.
**Material and Methods:** Pelvic MRI of 76 pCD patients were scored in random order by two radiologists blinded for all clinical information. MRI included (fat sat; FS) T2 and FS T1 after contrast medium. Scored items were MAGNIFI-CD index items and a rVAS of disease activity (0 no disease; 100 worst disease encountered). All items were analyzed for interobserver agreement with intraclass correlation coefficients (ICCs) for continuous variables and a weighted Kappa for ordinal variables.
**Results:** Both MAGNIFI-CD index and rVAS had substantial ICCs of 0.78 (95%CI 0.62–0.87) and 0.70 (95%CI 0.36–0.84). Individual items of the MAGNIFI-CD index had fair to substantial Kappa: dominant feature 0.36 (95%CI 0.18–0.53), fistula length 0.57 (95%CI 0.42–0.73), T1 hyperintensity 0.59 (95%CI 0.36–0.81), inflammatory mass 0.59 (95%CI 0.45–0.74), number of tracts 0.63 (95%CI 0.46–0.79), and extension 0.67 (95%CI 0.53–0.81).
**Conclusion:** MAGNIFI-CD index and rVAS show substantial reliability. Further studies should include assessment of responsiveness for treatment effects. For some items of the MAGNIFI-CD score (especially dominant feature), additional training or stricter definitions seem mandatory.

### *SS 6.9*

### Bowel involvement is related to systemic inflammatory response in COVID-19 patients

N. Chaniotaki, K. Dionysopoulos, G. Schinas, P. Zampakis, M. Marangos, K. Akinosoglou, C. Kalogeropoulou; *Patras/GR*

**Purpose:** To evaluate bowel involvement in hospitalizing patients with COVID-19 and their relation to lung disease extent and systemic inflammatory response.
**Material and Methods:** In this retrospective study, patients consecutively admitted to our hospital from March 2020 to November 2021, positive for SARS-CoV-2 were included. Abdominal CT performed in these patients were reviewed, and bowel abnormalities were recorded. Patients with prior abdominal pathology were excluded. Systemic inflammatory response was evaluated by measuring subcutaneous fat (SF) attenuation values in the anterior part of the abdomen. Chest CT-severity score (CT-SS) was also recorded for each patient.
**Results:** A total of 66 patients [average age 63.6 yrs (25–91 yrs), 41 men and 25 women] were evaluated. Bowel-wall abnormalities were seen on 39% of CT studies (26 of 66) and were associated with ICU admission (P = 0.033). Bowel wall thickening was identified in 30% [20/66] and included small bowel [n=8] and large bowel thickening [n=12]. Bowel dilatation was found in 33% [22/66], included small bowel [n=9] and large bowel [n=13]. SF attenuation, did not differ significantly between ICU and non-ICU patients (p=0.533). However, SF attenuation was significantly varied [p=0.028] between bowel involvement [median -109HU] and no bowel involvement subgroup [median -105HU]. Bowel abnormality group did not differ across CT-SS (p=0.153); however, dilatation of the large bowel was the only finding noted to differ significantly across the CT-SS subgroups (p=0.032).
**Conclusion:** Bowel abnormalities in COVID-19 patients are related to systemic inflammatory response as depicted by SF attenuation and strongly associated with ICU admission.

**11:00 - 12:30** **Auditorium 3 + 4**

*Scientific Session Live SS 7*

### Pancreas

### SS 7.1

### Arterial bleeding complicating acute necrotizing pancreatitis: risk factors and predictive features

A. Guilloux, M. Ronot, A. Jemal Turki, I. Bouktif, M. Gasperment, P. Levy, L. de Mestier, F. Maire, B. Jais, V. Rebours, D. Lorenzo; *Clichy/FR*

**Purpose:** To identify risk factors for arterial bleeding in patients with acute necrotizing pancreatitis (ANP).

**Material and Methods:** This is a retrospective case–control study including patients with ANP (2017–2019). Cases and controls were patients with and without arterial bleeding. Clinical, laboratory, imaging, and follow-up data were collected. Two radiologists retrospectively reviewed CT scans to identify culprit arteries, vascular lesions, presence and location of drains. A Cox–Mantel model was adjusted to identify risk factors for bleeding.

**Results:** A total of 148 patients (101 [68%] men, mean 54±14.6 yrs) was included. Twenty-eight (19%) patients experienced bleedings after a mean 66±50 days. Warning signs (abdominal pain, deglobulization) preceded bleeding in 8/28 (29%) cases. The main culprit arteries were the gastroduodenal (n=12, 40%) and splenic arteries (n=11, 37%). Vascular anomalies included pseudoaneurysms (n=10), arterial irregularities (n=11), narrowing (n=5) and interruption (n=3). At least one was present on the previous CT in 13 (46%) cases. A drain was present in 23/28 (82%) cases and 82/120 (68%) controls (p=0.17). It was in contact with the culprit artery in 10 (35%) cases. Multivariate analysis identified anticoagulation (OR=4.0, 95% CI 1.4–11.1), p=0.005) and low albumin (OR=1.13, 95% CI 1.02–1.25, p=0.025) as predictors of bleeding. Embolization was performed in 24 (86%) cases. Bleeding recurred in 7 (33%) cases. Admission in ICU and mortality rates were significantly higher in cases compared with controls (93% vs. 58%, p<0.001, and 29% vs. 6%, log-rank p=0.001).

**Conclusion:** Arterial bleeding occurs in one out of five patients with ANP. Vascular anomalies are frequent, and not limited to pseudoaneurysms. Curative anticoagulation appears as the main risk factor for bleeding.

### SS 7.2

### Fat stranding in acute pancreatitis can be a marker of severity. Is it influenced by the fat content of the body?

N. Faluhelyi, U. Straube, R. Pilisi, P. Bogner; *Pecs/HU*

**Purpose:** Acute pancreatitis is often examined with contrast-enhanced CT to determine possible reasons, complications and severity. For the latter, modified CT severity score (mCTSI) is the most frequently used tool. We observed that next to the aspects of mCTSI, the size of peripancreatic fat stranding (FS) might be another contributing factor in disease severity. We planned to test diagnostic value of FS and its relation to the fat volume of the body.

**Material and Methods:** CT examinations of 55 consecutive patients with confirmed acute pancreatitis in 2017 were retrospectively analysed. CTSI, peripancreatic FS (in largest axial diameter), sagittal abdominal diameter (SAD) at the level of umbilicus, and complete fat volume (with the help of 3D slicer) were determined. FS to SAD and to fat volume ratios were calculated.

**Results:** Spearman correlations were tested; CTSI and FS showed $r_s$=0.74452, CTSI and FS pro SAD showed $r_s$ = 0.71024, while CTSI and FS pro fat volume reached only $r_s$ = 0.57943. Fisher r-to-z transformation resulted in z=0.37 and ruled out significant difference (p=0.71) between the first two correlation ratios.

**Conclusion:** In conclusion, peripancreatic FS shows good correlation with CTSI, can be used as an easy to measure factor of disease severity. In addition, the measurements of fat volume and calculation of ratios did not improve this correlation, FS seems to be independent from body size. However, our pilot study is of limited value due to the small sample size.

### SS 7.3

### Can preoperative CT scan and bioimpedance vector analysis help to predict the development of postoperative pancreatic fistula?

C. Maino[1], T. Giandola[2], M. Ragusi[2], R. Corso[2], C. Talei Franzesi[1]; [1]*Milan/IT,* [2]*Monza/IT*

**Purpose:** To evaluate the accuracy of computed tomography (CT) and bioimpedance vector analysis (BIVA) in predicting pancreatic steatosis (PS) and development of postoperative pancreatic fistula (POPF).

**Material and Methods:** A total of 75 patients who underwent pancreatic resection having preoperative CT staging for pancreatic cancer were prospectively enrolled. All the unenhanced CT phases were analyzed to determine the overall mean attenuation value of the pancreas, expressed as Hounsfield unit (HU), excluding focal lesions. A radiologist drew three different regions of interest (ROIs), located in the head, body, and tail, to calculate the mean attenuation value. Histological data were used as standard of reference. BIVA was performed the day before surgery and pancreatic steatosis was assessed calculating fat mass index (FMI). Spearman correlation and receiver operating characteristic curve analysis were used to analyze and compare the different techniques with pathological specimens.

**Results:** The mean preoperative computed tomography pancreatic attenuation value was 18 (–3 to 39), and the mean FMI was 7.2 (3.4–11). Positive linear correlations were found between mean HU value and FMI when compared to histologic data (r= –0.852, p<0.001 and r= 0.652, p<0.001, respectively), and a good correlation was found between HU value and FMI (r= –0.659, p<0.001). All methods reported a good diagnostic accuracy in determining POPF, resulting in an area under the curve of 0.924 (95% CI: 0.844–1), 0.884 (95% CI: 0.778–0.990), and 0.942 (95% CI: 0.879–1) for mean HU value, FMI, and histology, respectively.

**Conclusion:** Pre-operative fat mass evaluation by mean of unenhanced CT attenuation value and BIVA can be considered good predictors of POPF and also a reliable approach to quantify the pancreatic steatosis.

### SS 7.4

### Quantitative imaging analysis predicts pancreatic fatty infiltration on routine CT

C. Previtali[1], R. Sartoris[2], M. Dioguardi Burgio[1], A. Couvelard[2], V. Rebours[2], A. Sauvanet[2], V. Vilgrain[2], M. Ronot[2]; [1]*Paris/FR,* [2]*Clichy/FR*

**Purpose:** To assess the performance of quantitative CT imaging for the detection of pancreatic fatty infiltration using pathology as reference.

**Material and Methods:** Retrospective study including 60 patients (33 women [55%], mean age 56±12 years) undergoing pancreatic surgery for pancreatic tumor from 2016 to 2019. Patients with dilation of the main pancreatic duct, chronic pancreatitis, or preoperative treatment were excluded to ensure proper rad-path correlation. Pancreatic fatty infiltration was defined at pathology. Pancreatic surface lobularity (PSL), pancreatic attenuation (PA), visceral (VFA) and subcutaneous fat area (SFA) were measured on preoperative CT images. Performance for the prediction of fatty infiltration was assessed with receiver operating characteristic curve and backward binary logistic regression analysis. Results were validated in a separate cohort of 34 patients (17 women [50%], mean age 50±14 years).

**Results:** A total of 28/60 (47%) and 17/34 (50%) patients had pancreatic fatty infiltration in the derivation and validation cohort, respectively. In the derivation cohort, patients with fatty infiltration had significantly higher PSL (p<0.001), lower PA on both pre-contrast and portal venous phase (PVP) (p=0.010 and 0.003, respectively) and higher SFA and VFA (p=0.010 and 0.007, respectively). PSL > 7.6 and PA on PVP < 83.5 HU were identified as optimal cut-off values to identify fatty infiltration. Multivariate analysis identified PSL > 7.6 and PA on PVP < 83.5 HU as independently associated with fatty infiltration. Their combination resulted in an AUC of 0.85 (95% CI 0.74–0.95) and 0.83 (95% CI 0.67–0.99) in the derivation and validation cohorts, respectively.

**Conclusion:** CT-based quantitative imaging accurately predicts pancreatic fatty infiltration.

### SS 7.5

### Histopathologic correlation of magnetic resonance T1 signal intensity and enhancement ratio of pancreas with pancreatic fibrosis: comparison with Cambridge Classification

T. Tirkes, O. Saeed, V. Osuji, C. Kranz, A. A. Roth, N. Zyromski, E. Fogel; *Indianapolis, IN/US*

**Purpose:** To assess the histopathologic correlation of the T1-weighted signal intensity ratio of the pancreas (T1 SIR), arterial-to-venous enhancement ratio (AVR) of the pancreas and Cambridge grade with pancreatic fibrosis.

**Material and Methods:** We identified 160 adult patients who underwent pancreatic resection between 2017 and 2019. Sixty of these patients who had MRI/MRCP within 180 days prior to surgery were analyzed. Histopathologic grading was given using Ammann's fibrosis score. Three blinded observers independently measured the T1 SIR pancreas to spleen (T1 SIR p/s), T1 SIR pancreas to paraspinal muscle (T1 SIR p/m), AVR and Cambridge grade.

**Results:** The study population included 33 female and 27 male patients, with an average age of 52.1 (26–78 years). Correlations between fibrosis score and T1 SIR p/s, T1 SIR p/m and AVR were (ρ= −0.54, p=0.0001, ρ= −0.19, p=0.19, and ρ= −0.39, p=0.003, respectively). Correlation between the fibrosis score and Cambridge grade was ρ= 0.26, p=0.07. Interobserver agreement (weighted kappa) for T1 SIR p/s, T1 SIR p/m and AVR was substantial (0.78, 0.71 and 0.64, respectively). T1 SIR p/s of 1.20 or less provided sensitivity of 74% and specificity of 50% (area under the curve (AUC): 0.74) while AVR of 1.10 or less provided sensitivity of 75% and specificity of 55% (AUC: 0.68) to detect fibrosis score of 6 and higher.

**Conclusion:** There is a moderate negative correlation between the T1 SIR p/s and AVR with pancreatic fibrosis, together with substantial interobserver agreement for measurement. This correlation is higher than the correlation between the Cambridge grade and pancreatic fibrosis.

### SS 7.6

### High prevalence of multi-organ steatosis and fibroinflammation, identified by multiparametric magnetic resonance imaging, in people with type 2 diabetes

N. Eichert[1], K. Gibbons[1], A. Hamid[2], R. Ramkhelawon[3], J. Woolgar[1], R. Suriano[1], M. Pansini[1], H. Thomaides-Brears[1], R. Banerjee[1], G. Kemp[1], S. Ali[3], G. Thanabalasingham[1], D.J. Cuthbertson[1]; [1]*Oxford/UK*, [2]*Liverpool/UK*, [3]*London/UK*

**Purpose:** Type 2 diabetes (T2D) is a multi-system disease characterised by a high prevalence of co-morbidities and micro- and macro-vascular complications. This study aimed to provide comprehensive assessment of multi-organ health and pre-clinical detection of diabetes-related organ damage with a single, non-contrast MRI scan.

**Material and Methods:** 138 adults with T2D [62 yrs (54–70); 60% male; BMI of 31 kg/m$^2$ (28–35); 92% on metformin] were recruited. MRI data were acquired to derive organ-specific measures of size, fat deposition, fibroinflammation, body composition and aortic distensibility (CoverScan, Perspectum). Normative values of MRI metrics were based on 92 healthy volunteers and published literature. Statistical significance of co-prevalence was assessed by simulation assuming that values were independently binomially distributed.

**Results:** There was a high prevalence of multi-organ abnormality (86% with at least 2 organs affected). There was evidence of fatty infiltration and/or fibroinflammatory changes in the liver (72% of patients), co-occurring with abnormality in the pancreas (38%), spleen (44%), kidney (64%), and aorta (71%). High-risk non-alcoholic steatohepatitis (NASH) was significantly more prevalent in the obese ($p < 0.005$). Prevalence of low SMI and elevated pancreatic fat were associated with the duration of T2D ($p < 0.05$). Aortic stiffness was significantly associated with organ steatosis ($p < 0.05$).

**Conclusion:** Elevated levels of fat and fibroinflammation were highly prevalent in the liver, pancreas, spleen, and kidney with evidence of atherosclerosis and sarcopenia in most patients with T2D.

### SS 7.7

### Breathing task paradigm to improve the quality of pancreatic magnetic resonance elastography

N.P. Wassenaar[1], A.-S. van Schelt[1], E.M. Schrauben[1], R. van der Woude[1], J.E. de Jong[1], J.L. Nelissen[1], H.W. van Laarhoven[1], J. Guo[2], I. Sack[2], J. Runge[1], A.J. Nederveen[1], J. Stoker[1]; [1]*Amsterdam/NL*, [2]*Berlin/DE*

**Purpose:** Pancreatic MR elastography (MRE) can detect viscoelastic changes in the course of pancreatitis or pancreatic cancer. Free-breathing (FB) MRE acquisition can lead to mismatches in pancreatic location throughout the scan, potentially introducing errors in stiffness reconstruction. Breathing task (BT) paradigm timed such that MRE acquisition for a given slice occurs during the same respiratory phase could potentially increase MRE quality.

**Material and Methods:** Nine healthy volunteers (female=6, mean age=27±2years) underwent three MRE scans: FB-MRE scans with standard interleaved slice ordering and two MRE scans with synchronized BTs using interleaved and reversed central slice-order (BT-Int and BT-Rev, respectively). BT-Rev was used to test if central slices containing the pancreas within the multi-slice acquisition were scanned during end-expiration. The dice similarity coefficient (DSC) of the pancreatic region of interest drawn in three slices over eight phase-offsets was calculated. Strain-SNR was determined in the pancreas and in the whole abdomen. Repeated measures ANOVA with pairwise comparison and Bonferroni correction was used for statistical analysis ($p<0.05$).

**Results:** DSC was significantly higher for BT-Rev compared with FB-MRE only (p=0.026). No significant differences were found in pancreatic SWS and strain-SNR but whole abdomen strain-SNR was significantly higher for BT-Rev compared to FB and BT-Int (p value=0.010 and 0.014, respectively).

**Conclusion:** Though using breathing paradigms did not improve pancreatic strain-SNR nor SWS, it did improve whole abdomen strain-SNR and synchronization of pancreas location with image acquisition. Further investigations should focus on whether breathing paradigms increase MRE's reproducibility, thereby improving its sensitivity to detect smaller changes in viscoelasticity.

### SS 7.8

### Feasibility of compressed sensing accelerated Ristretto magnetic resonance elastography in the pancreas

A.-S. van Schelt[1], N.P. Wassenaar[1], J. Runge[1], M. Troelstra[2], J.L. Nelissen[1], C. Guenthner[3], R. Sinkus[4], J. Stoker[1], A.J. Nederveen[1], E.M. Schrauben[1]; [1]*Amsterdam/NL*, [2]*London/UK*, [3]*Zurich/CH*, [4]*Paris/FR*

**Purpose:** Magnetic resonance elastography (MRE) allows for non-invasive determination of pancreatic viscoelastic properties. Longitudinal changes in tissue stiffness in the course of pancreatitis, endocrine dysfunction or pancreatic cancer could reveal new insights into disease pathophysiology. Quantitative accuracy of MRE in small structures depends on local stability during acquisition. However, some MRE implementations require multiple consecutive breath-holds (BHs). These may introduce errors in pancreas location, limit spatial and temporal resolution and can be particularly uncomfortable for patients. The aim of this study was to develop and test a single BH compressed sensing (CS) accelerated MRE acquisition.

**Material and Methods:** MRE was performed on six healthy volunteers (♀=6, mean age=27±3 years). Six consecutive pancreatic MRE scans were performed: (I) standard multi-slice (MS), (II) 3D SENSE-accelerated four BH acquisition, (III) 3D CS-accelerated four BH acquisition and (IV–VI) three 3D CS-accelerated single BH acquisitions (BH=20, 18, 13s, respectively). Mean shear-wave-speed (SWS) and nonlinearity (quality parameter) in the pancreas were analyzed, with II allocated as index test for CS. Statistical analysis was done using repeated measures ANOVA and pairwise comparison with Bonferroni correction ($p<.05$).

**Results:** CS-accelerated MRE gave comparable stiffness values up to BH=18s. Nonlinearity showed no significant difference between all scans. However, accuracy of MRE inversion is only guaranteed for nonlinearity<50%; therefore, VI (BH=13s, nonlinearity=50.5%) could be considered borderline acceptable.

**Conclusion:** CS-accelerated single BH (BH=18s) MRE is feasible without hampering viscoelastic reconstruction in tissues with low shear stiffness. Further research is necessary to guarantee accuracy of the measured SWS, notably for high stiffness tissues such as tumors, and at higher acceleration factors.

### SS 7.9

### Abdominal imaging manifestations in COVID-19 and mucormycosis superinfection: experience from a tertiary care hospital in India

H. Goyal, B. Sureka, P. Khera, P. Garg, T. Yadav, S. Tiwari, A. Agarwal, V. Varshney, R. Rajagopal, M. K. Garg, M. Banerjee, N. Kothari, A. Agarwal, G. Choudhary; *Jodhpur/IN*

**Purpose:** COVID-19 is a multisystem disorder, caused by SARS-CoV-2. Incidence of mucormycosis superinfection has drastically increased in COVID-19 patients. Abdominal involvement in COVID-19 and COVID-19-associated mucormycosis (CAM) leads to significant increase in morbidity and mortality. The aims and objectives are as follows: 1. to identify the indications of abdominal CT in COVID-19; 2. to see the imaging findings in abdominal mucormycosis superinfection; 3. to study the spectrum of abdominal findings in COVID-19.
**Material and Methods:** Combined retrospective and prospective single-group observational study duration: March 2020–November 2021. Sample size: all patients (68 COVID and 7 CAM) who had abdominal complaints and required abdominal CT for assessment within the time period between March 2020 and November 2021. Scan protocols were optimized according to the patients' complains and clinical suspicion of treating physicians. Three radiologists (with 12 years, 10 years and 1 year of experience) interpreted the patients' CT images. Data collection: 1. abdominal CT findings and 2. relevant lab parameters and demographic details.
**Results:** 1. Abdominal manifestations were more common in ICU patients and elderly population. 2. Bowel wall ischemic changes, vascular thrombosis, solid organ infarcts and pancreatitis were most significant findings in COVID-19 and mucormycosis superinfection. 3. Abdominal pain was the most common indication for abdominal imaging in COVID-19.
**Conclusion:** Abdominal manifestations in COVID-19 and CAM can significantly increase morbidity and mortality in patients and cause extra burden on healthcare system. These conditions should be promptly suspected, evaluated and diagnosed to swiftly start the treatment and improve patients' outcome.

### SS 7.10

### The relationship of pancreatic and hepatic steatosis assessed on CT with COVID-19 pneumonia severity score and prognosis

H. Dogan[1], E. Uzer[1], O.T. Esengur[1], H. Hassoy[2], S. Guneyli[2]; [1]*Istanbul/TR,* [2]*Izmir/TR*

**Purpose:** To investigate the relationship of pancreatic steatosis (PS) and hepatic steatosis (HS) assessed on computed tomography (CT) with COVID-19 pneumonia severity score and prognosis.
**Material and Methods:** This retrospective study covers a total of 461 patients (255 male and 206 female patients, mean age of 52.27 ± 16.60 years) with a positive result of reverse transcription-polymerase chain reaction test for COVID-19, who underwent unenhanced chest CT. PS and HS assessed on CT were compared with patient demographics, pneumonia severity score, comorbidities, requirement of hospitalization, requirement of intubation, and mortality rates. Numerical parameters were compared using Student's t test, while categorical parameters were compared using chi-square test.
**Results:** There were 151 patients with PS and 104 patients with HS. Pneumonia severity scores were higher in patients with PS ($p < 0.001$) and in patients with HS ($p < 0.001$) than those in patients without PS and HS, respectively. Higher rates of requirement of hospitalization in patients with PS ($p < 0.001$) and in patients with HS ($p = 0.004$) were demonstrated compared to those in patients without PS and HS, respectively. Requirement of intubation ($p = 0.003$) and mortality rates ($p = 0.011$) were found to be significant in only patients with PS.
**Conclusion:** Pneumonia severity score and requirement of hospitalization correlated with PS and HS in COVID-19 patients. However, only PS was found to be as a prognostic factor for requirement of intubation and mortality rates in COVID-19 patients.

**11:00 - 12:30** **Auditorium 8**

*Scientific Session Live SS 8*

### Biliary tract/NET

### SS 8.2

### Simple diagnosis of relevant strictures in primary sclerosing cholangitis patients using gadoxetic acid-enhanced MRI

S. Poetter-Lang, N. Bastati-Huber, A. Messner, A. Kristic, R. Ambros, A. Ba-Ssalamah; *Vienna/AT*

**Purpose:** Relevant stricture (RS) in PSC patients has significant clinical and prognostic impact. However, RS definition and diagnosis remain controversial, relying upon morphology on T2-MRCP, which has a high interobserver variability. We introduce a function-based RS definition on T1-weighted hepatobiliary phase (HBP) images of gadoxetic acid (GA)-enhanced MR cholangiography (T1-HBP-MRC). Our purpose was to compare T1-HBP-MRC to T2-MRCP in distinguishing non-RS from RS and hepatocellular dysfunction (HD), and to correlate these with outcomes in PSC patients.
**Material and Methods:** Six independent readers blinded to patient data reviewed 130 MRIs, diagnosing RS on T2-MRCP using several guidelines. On T1-HBP-MRC images, RS was diagnosed if no GA excretion was seen in the common bile duct or the distal right or left hepatic duct. If, additionally, liver cirrhosis was present, HD was diagnosed. The three diagnostic groups from T1-HBP-MRC (non-RS=86; RS=32; HD=12) were correlated with clinical scores, labs, splenic volume, and outcomes. Statistical analyses included Kaplan–Meier curves, decision-tree, and Cox regression.
**Results:** Interobserver agreement was excellent for non-RS/RS/HD diagnosis on T1-HBP-MRC ($\kappa$=0.82), but poor to fair for T2-MRCP ($\kappa$=0.13-0.31). Laboratory tests and clinical scores were significantly worse in RS and HD versus non-RS patients ($p$<0.001). There were more clinical events in RS or HD than in non-RS patients ($p$<0.05). Non-RS/RS/HD diagnoses on T1-HBP-MRC correlated ($p$<0.001) with outcomes, whereas measurement-based T2-MRCP diagnoses did not ($p$>0.05). Multivariate analysis found T1-HBP-MRC-derived diagnosis was an independent risk factor for morbidity and mortality.
**Conclusion:** T1-HBP-MRC stratifies PSC patients into non-RS/RS/HD, which correlates with outcomes. Conventional T2-MRCP measurement-based criteria cannot.

Scientific Sessions

Authors' Index

### SS 8.3

### Development and assessment of prognostic value and agreement of a novel MRCP score for patients with primary sclerosing cholangitis

A. Grigoriadis[1], K.I. Ringe[2], J. Bengtsson[3], E. Baubeta-Fridh[3], C. Forsman[4], N. Korsavidou-Hult[4], F. Rorsman[4], E. Nilsson[3], A. Bergquist[1], N. Kartalis[1]; *[1]Stockholm/SE, [2]Hannover/DE, [3]Lund/SE, [4]Uppsala/SE*

**Purpose:** To develop, evaluate the reproducibility and prognostic value of a novel MRCP-based score for patients with primary sclerosing cholangitis (PSC).

**Material and Methods:** The MRCP score was developed based on the extent and severity of cholangiographic changes of intrahepatic and extrahepatic bile ducts (range 0–8) in coronal 3D MRCP sequences. In this ethics review-board-approved multicenter study, three pairs of radiologists (each pair from a national tertiary center) applied the score on MRCP examinations of a total of 219 patients (103, 51, and 65, respectively) from a prospectively collected cohort with a median follow-up of 7.3 years. Interreader and intrareader agreement was assessed with intraclass correlation coefficient (ICC). After consensus reading, the prognostic value of the score was assessed with Cox-regression analysis and outcome-free survival rates with Kaplan–Meier estimates. Area under the curve (AUC) was calculated and a 10-fold cross-validation was performed.

**Results:** 56 patients developed outcomes (i.e., liver transplantation, liver decompensation and liver-related death). Interreader agreement for radiologists from two tertiary centers was good (ICC=0.81, 95%CI: 0.74–0.89, and ICC=0.81, 95%CI: 0.70–0.88, respectively). Intrareader agreement was from good to excellent (ICC=0.93, 95%CI: 0.90–0.95, and ICC=0.87, 95%CI: 0.80–0.92, respectively). Patients with MRCP score 5–8 had four times higher risk (hazard ratio: 4.13, 95%CI: 2.37–7.19) for developing outcomes, and significantly lower survival rates (p<0.000), compared to those with MRCP score 0–4. AUC for the MRCP score was 0.76 and 0.74 after cross-validation.

**Conclusion:** The novel MRCP score is reproducible and strongly associated with clinical outcomes which indicates its value for the prognosis of patients with PSC.

### SS 8.4

### Changes in ductal disease captured by quantitative magnetic resonance cholangiopancreatography in primary sclerosing cholangitis

P.J. Trivedi[1], K. Arndtz[1], N. Abbas[1], M. Pansini[2], A. Telford[2], L. Young[2], C. Ferreira[3], R. Banerjee[2], P. Eddowe[4], K.S. Jhaveri[5], G.M. Hirschfield[5]; *[1]Birmingham/UK, [2]Oxford/UK, [3]Aveiro/PT, [4]Nottingham/UK, [5]Toronto, ON/CA*

**Purpose:** Primary sclerosing cholangitis (PSC) is a chronic, progressive liver disease, characterised by multi-focal stricturing throughout the biliary tree. Presently, no medical treatment is proven to slow disease course and clinical trials lack a quantitative radiological toolkit to accurately monitor disease progression. This study evaluated the utility of quantitative MRCP (MRCP+™) for characterising ductal disease progression over time in PSC.

**Material and Methods:** Patients with PSC (n=27) underwent prospective standardised MRCP imaging at baseline and 1-year thereafter. Scans were processed with MRCP+ (Perspectum Ltd) to enhance and quantify biliary structures. 24 MRCP+ metrics were analysed. Temporal variation was assessed using linear mixed-effects models.

**Results:** Non-cirrhotic patients with PSC were enrolled (median age 45 yr) with baseline PSC Mayo Risk Scores of −0.93 (IQR −1.2 to 0.3) and transient elastography scores of 7.7 kPa (IQR 6.2–11.5). Over a median 371-day follow-up, 67% of patients manifested features of ductal disease change, evidenced by an increase in the total number of strictures (p = 0.036), the total length of strictures (p = 0.030), the total stricture burden (p = 0.012), or a combination of these metrics. Liver biochemistry (p > 0.39), transient elastography score (p = 0.07) and the modified Amsterdam cholangiographic score (p = 0.80) did not change significantly over the same period.

**Conclusion:** Changes in MRCP+ metrics reflected ductal disease change in most patients with PSC over 1 year, despite liver biochemistry and fibrosis values remaining constant. Longer term observational studies are needed to assess the impact of these changes on patient's clinical outcomes.

### SS 8.5

### Prediction of intracholecystic papillary neoplasm with associated invasive carcinoma of the gallbladder using preoperative contrast-enhanced computed tomography and ultrasound

J.H. Kim, H.-J. Kang, J. Bae, J.H. Kim; *Seoul/KR*

**Purpose:** To assess the important features for predicting intracholecystic papillary neoplasm with associated invasive carcinoma (ICPN-IC) of the gallbladder (GB) using contrast-enhanced CT or US.

**Material and Methods:** This retrospective study enrolled 119 patients who underwent preoperative CT or US with pathologically confirmed ICPN of the GB (low-grade dysplasia = 34, high-grade dysplasia = 35, invasive carcinoma = 50). Two radiologists independently assessed image findings focusing on wall and polypoid lesion characters. They also graded the likelihood of ICPN-IC. Univariate and multivariate analyses were performed to identify significant predictors for ICPN-IC. The performance of CT and US was evaluated based on area under the receiver operating characteristic (AUROC) curves.

**Results:** Among 50 ICPN-IC (42%, 50/119), 25 (50%) showed T1 stage. Wall thickness was significantly different in ICPN-IC (5.38 ± 4.02 vs 3.12 ± 1.42, p< 0.0001). Wall thickness (odds ratio (OR), 1.4; 95% confidence interval (CI), 1.1–1.9) and mucosal discontinuity (OR, 5.6; 1.3–23.4) were independently associated with ICPN-IC in CT (p< 0.05). Among 119 ICPNs, 110 (92.4%) showed polypoid lesion. In polypoid lesion, multiplicity (OR, 4.0; 1.6–10.4) and polypoid lesion base wall thickening (OR, 6.0; 2.3–15.8) were independently associated with invasive carcinoma in CT, while polyp size (OR, 1.1; 1.0–1.2) was a significant predictor of invasive carcinoma in US (p< 0.05). The diagnostic performances for predicting ICPN-IC were better in CT (AUROC, 0.806 and 0.786) than US (AUROC, 0.706 and 0.647).

**Conclusion:** ICPN provided high incidence of ICPN-IC. Thick wall, presence of polypoid lesion base wall thickening, multiplicity, and large size of polyp could be useful findings favoring ICPN-IC.

### SS 8.6

### Metastatic pattern of carcinoma gallbladder on abdominal CT scan and its prognostic impact

G.R. Agarwal, Y. Gupta, N. Singh, S. Narayan, R. Yagnik, A. Gandhi, G. Gupta, P. Singh; *Lucknow/IN*

**Purpose:** This work aimed to study distribution of metastatic pattern of carcinoma gallbladder and to determine the prognostic impact of metastatic pattern on clinical outcome.

**Material and Methods:** This prospective cohort study was conducted on 74 patients of metastatic carcinoma gallbladder who underwent triple phase computed tomography between December 2019 and July 2021.

**Results:** In our study, we found that the most common pattern identified is lymphatic spread, i.e., 56(75.7%) cases. Most common lymph node to be involved is periportal, i.e., 48(85.7%) cases. Most common lymph nodal combination identified is periportal + aortocaval + paraaortic, i.e., 13(23.2%) cases. The 2nd most common site identified is liver, i.e., 45 (60.8%) cases. Less commonly involved sites are ovary 2(2.7%) cases, bones 4(5.4%) cases, adrenal 4(5.4%) cases, abdominal wall 3(4.1%) cases and lungs 5(6.8%.) cases. Survival of patients is not associated with metastatic sites of carcinoma gallbladder (p value >0.05); however, it has been seen in our study that the patients with peritoneal carcinomatosis have poor survival, i.e., <3 months (p value 0.036). In our study, patients who received palliative chemotherapy have better survival as compared to those who did not receive (5.90 ± 1.25 months vs 4.06 ± 1.61 months) (p value 0.001).

**Conclusion:** Metastatic spread in gallbladder carcinoma is predominately lymphatic with the combination of periportal, aortocaval and paraaortic lymph nodal involvement. Liver is the 2nd most common site. Uncommon sites are also seen to be involved in gallbladder malignancy. Survival of patient is not site specific for metastasis; however, peritoneal carcinomatosis is an exception. Patients subjected to palliative chemotherapy have better survival.

### *SS 8.7*

### Changes in volumetric enhancing tumor burden on CT predicts survival outcomes in patients with neuroendocrine liver metastases after intra-arterial treatments

J. Assouline[1], R. Cannella[2], G. Porrello[2], L. de Louis[1], M. Dioguardi Burgio[3], M.-P. Vullierme[1], O. Hentic[1], V. Vilgrain[1], R. Duran[4], M. Ronot[1]; [1]*Clichy/FR,* [2]*Palermo/IT,* [3]*Paris/FR,* [4]*Lausanne/CH*

**Purpose:** To investigate whether the liver enhancing tumor burden (LETB) assessed on contrast-enhanced CT could be a biomarker of early response and help predict survival outcomes in patients with multifocal neuroendocrine liver metastases (NELM) after intra-arterial treatment (IAT).
**Material and Methods:** This retrospective study included patients with NELM who underwent IAT with either transarterial embolization or chemoembolization. Tumor response in treated NELM was evaluated by the Response Evaluation Criteria in Solid Tumors (RECIST) and modified RECIST (mRECIST). LETB was measured as more than two standard deviations the attenuation of a region of interest in the nontumoral liver parenchyma using dedicated software. Overall survival (OS), time-to-unTA(C)Eable progression, hepatic and whole-body progression-free survival (PFS) were evaluated using multivariable Cox proportional hazards analyses, the Kaplan–Meier method, and the log-rank test.
**Results:** The study included 119 patients (mean age 59.8±10.7 years, 61 [51.3%] men) who underwent 161 treatments. A median LETB change of –25.8% best discriminated OS (83 *vs* 51 months in responders and nonresponders, respectively; *P*=0.021) and whole-body PFS (18 *vs* 8 months; *P*<0.001). An LETB change of –10% best discriminated time-to-unTA(C)Eable progression (32 *vs* 12 months, respectively; *P*<0.001) and hepatic PFS (18 *vs* 8 months, respectively; *P*<0.001). The LETB change remained independently associated with improved OS (hazard ratio (HR): 0.56), time-to-unTA(C)Eable progression (HR: 0.49), hepatic PFS (HR: 0.42), and whole-body PFS (HR: 0.47) on multivariable analysis. Neither RECIST nor mRECIST were predictors of patient outcome.
**Conclusion:** Response according to the change in LETB predicts survival outcomes in patients with NELM after IATs, with better discrimination than RECIST and mRECIST.

### *SS 8.9*

### CT texture end contrast enhancement features in preoperative pancreatic neuroendocrine tumors prediction

I. Gruzdev, V. Tikhonova, K. Zamyatina, V. Struchkov, E. Kondratyev, G. Karmazanovsky; *Moscow/RU*

**Purpose:** Pancreatic neuroendocrine tumor (PNET) treatment and prognosis based on the tumor grade. The texture and contrast enhancement features of pancreatic neuroendocrine tumors (PNETs) vary depending on the tumor grade and could be noninvasive predictors. The purpose of this study was to identify the best contrast enhancement and texture features for PNET grade prediction.
**Material and Methods:** 81 patients with 82 histologically proven hypervascular PNETs of different grades were selected in the study. All the patients underwent preoperative contrast-enhanced CT (CECT) with unenhanced, arterial, venous, and delayed phases. Immunohistochemistry was done to determine the tumor grade based on Ki-67 and mitotic count. We calculated lesion-to-parenchyma contrast (LPC) and relative tumor enhancement ratio (RTE) for contrast-enhanced phases. We extracted 52 texture features for each phase of the study. Contrast enhancement and texture features were compared between grade 1 and grade 2/3 PNETs. The LASSO regression was used to select predictors and build the diagnostic model.
**Results:** For tumor grade prediction in the final diagnostic model, 3 predictors were selected: arterial RTE and GLZLM_ZLNU, and venous SHAPE_Compacity. The resulting model had high discriminative characteristics (AUC = 88%) and showed sensitivity, specificity, and accuracy of 67.6%, 97.7%, and 84%, respectively, in the prediction of grade 2/3 PNETs.
**Conclusion:** The developed diagnostic model based on contrast and textural features has a high accuracy in determining the PNETS grade and can be used to determine the prognosis and treatment tactics in complicated clinical cases.

### *SS 8.10*

### Treatment assessment of PNET and NELM after everolimus by quantitative MRI parameters

M.K. Ingenerf, J. Ricke, C. Auernhammer, S. Kiesl, J. Rübenthaler, F. Berger, C. Schmid-Tannwald; *Munich/DE*

**Purpose:** To investigate quantitative MRI parameters in the assessment of therapy response of pancreatic neuroendocrine tumors (pNET) and their hepatic metastases (NELM) after everolimus.
**Material and Methods:** Retrospectively, 17 patients with 41 target lesions who were treated with everolimus and had abdominal MRI before and after therapy were included. Intralesional (primarius and if possible two NELM per patient) signal intensity (SI) of noncontrast T1 and apparent diffusion coefficients (ADCmean and ADCmin) were measured on baseline and follow-up MRI. Response assessment was categorized according to progression-free survival (PFS) defined by the local interdisciplinary tumor board. Responders (R) were defined by PFS of more than 11 months and non-responders (NR) less than 11 months.
**Results:** ADCmin of NELM decreased under everolimus in the R group whereas it increased in the NR group showing a significant difference before and after the start of everolimus comparing R and NR (p=.009). The change of ADCmin of NELM before and after therapy start correlated with PFS (0.825). By contrast, the change of ADCmin in pNETs was increasing in the R group and decreasing in the NR group. SI of T1 of NELM increased in the R group and decreased in the NR group showing a significant difference (p<.001). In the pNETs, T1 SI decreased in the R group and increased in the NR group.
**Conclusion:** DWI seems to allow treatment assessment of pNET and NELM after everolimus. Interestingly, there might be an opposite effect on MRI parameters of NELM in patients with pNET responding to everolimus.

**14:30 - 16:00** **Auditorium 3 + 4**

*Scientific Session Live SS 9*

### Small bowel and colon

### SS 9.1

### Factors predicting transmural necrosis in patients with acute mesenteric venous thrombosis

F. Salahshour[1], E. Shaker[1], A. Abkhoo[1], F. Shojaeshafiei[2], N. Ayoobi Yazdi[1]; [1]*Tehran/IR,* [2]*Ramsar/IR*

**Purpose:** To determine the clinical, laboratory, and computed tomography (CT) findings that indicate transmural necrosis in patients with acute mesenteric venous thrombosis (AMVT).

**Material and Methods:** We used the electronic records and CT scans of the patients hospitalized between 2014 and 2020 with the diagnosis of acute mesenteric ischemia due to AMVT. Various parameters were compared between the patients who had transmural necrosis on laparotomy and those without transmural necrosis who could be discharged from the hospital.

**Results:** Of 51 patients, 18 underwent laparotomy. Among them, 13 had transmural necrosis whereas, 5 patients had normal or ischemic bowel. 33 completed conservative therapy. Among the clinical findings, obstipation, and among the laboratory findings, the mean level of white blood cells, creatinine, urea, and lactate dehydrogenase were higher in the transmural necrosis group. Comparing the CT scan findings, the mean diameter of the abnormal loop, and diameter of the bowel loop proximal to the abnormal loop were higher in the patients with transmural necrosis. Also, these radiologic findings were more prevalent in the transmural necrosis group: loculated fluid beside abnormal loop, bowel wall thickening, adjacent focal mesenteric haziness, foci with decreased enhancement, and pneumatosis intestinalis. Among 32 patients with abnormal bowel wall thickening, the diameter of the abnormal loop, the diameter of the bowel loop proximal to the abnormal loop, and adjacent focal mesenteric haziness were higher in those with transmural necrosis.

**Conclusion:** Clinical, laboratory and radiologic findings could be used to differentiate transmural necrosis from non-transmural necrosis in patients with AMVT.

### SS 9.2

### Acute mesenteric ischemia: diagnostic value of QT parameters and their relationship with CT findings

B. Alan, S. Gurel, S. Alan; *Bolu/TR*

**Purpose:** Our aim to investigate diagnostic value of electrocardiographic QT parameters in acute mesenteric ischemia (AMI) and their relationship with CT findings.

**Material and Methods:** Patients referring to emergency department with abdominal pain were recruited from HIS. Grouping were made on the basis of AMI and Non-AMI. In both groups, QTcorrected (QTc) and QTdispersion (QTD) were measured on electrocardiographs while qualitative and quantitative CT findings were evaluated on CT examinations, patients with atrial fibrillation were excluded from the study

**Results:** QTc and QTD values were higher in the AMI group, respectively, QTc; 456.16 (422.88–483.16), 388.83 (359.74–415.83), p<0.001. QTD; 58 (50.3–68.25), 46 (42–50), p<0.001. A positive correlation was found between QTc and QTD and AMI Qtc (OR = 1.068, 95% CI: 01.041–1.096, p<0.001) and QTD (OR = 1.186, 95% CI: 1.093–1.287, p<0.001). AUC for QTC: 0.903 (95%CI: 0.857–0.950, p<0.001), sensitivity of 80.8% and a specificity of 82.3%. AUC for QTD: 0.821 (95%CI: 0.753–0.889, p<0.001) Sensitivity of 73.1% and a specificity of 82.3%. In CT analysis, QTc values were significantly higher in AMI patients with images of paper-thin bowel wall and absence of bowel wall enhancement. At the same time, QTD values were significantly higher in patients with venous pneumatosis findings on CT.

**Conclusion:** We found QTc and QTD values to be significantly higher in AMI patients. We saw a significant relationship between CT findings and QTc and QTD. In addition, we found a significant relationship between survival and QTc in the AMI group.

### SS 9.3

### Are qualitative and quantitative CT parameters prognostic in non-occlusive mesenteric ischemia?

B. Alan, S. Gurel; *Bolu/TR*

**Purpose:** Our aim is to evaluate the predictive value of quantitative and qualitative CT parameters in the prognosis of non-occlusive mesenteric ischemia (NOMI).

**Material and Methods:** In this study, 40 patients with diagnosis of NOMI between 2016 and 2021 were analyzed retrospectively. Patients were grouped as survivor and nonsurvivor. CT findings were classified as qualitative (vascular, intestinal, mesenteric, peritoneal, other visceral findings, comorbidities, surgical resection findings) and quantitative (diameters of superior mesenteric artery (SMA), celiac trunk (CT), inferior vena cava (IVC), superior mesenteric vein (SMV), bowel wall contrast enhancement difference (Delta HU)) parameters. Qualitative and quantitative CT parameters and patient prognosis were evaluated using Fisher's exact test, ANOVA test, logistic regression analysis and ROC analysis, respectively.

**Results:** Pneumatosis intestinalis (p=0.012), paper-thin bowel wall (p=0.015), and pale mesentery (p=0.008) were found to be higher in the non-survivor group. Mesenteric congestion (p=0.003), bowel wall thickening (p=0.001), bowel wall enhancement (p=0.044) and enhancing mucosa and edematous submucosa of bowel wall (p=0.042) were mostly found in the survivor group. Diameters of SMA, IMA, SMV and IVC, arterial enhancement of bowel wall and delta HU were significantly lower in the non-survivor group. Significant relationship was found with the VCI diameter in regression analysis. For VCI, high AUC values were detected. In the ROC analysis, AUC: 0.795 (95%CI: 0.658–0.932, p=0.002).

**Conclusion:** Pale mesentery, pneumatosis intestinalis, paper-thin bowel wall are qualitative, decreased SMA, IMA, SMV, IVC diameter, bowel wall enhancement are quantitative bad prognostic parameters which might contribute to early diagnosis and improvement in prognosis of NOMI.

### SS 9.4

### Reperfusion injury on computed tomography following endovascular revascularization of early acute mesenteric ischemia: incidence, risks factors and outcome

L. Garzelli[1], A. Nuzzo[1], A. Hamon[1], I. Ben Abdallah[2], L. Paulatto[1], L. Raynaud[1], Y. Castier[2], Y. Panis[1], O. Corcos[1], V. Vilgrain[1], M. Ronot[1]; [1]*Clichy/FR,* [2]*Paris/FR*

**Purpose:** To assess the prevalence and to identify risk factors of reperfusion injury (RI) after endovascular revascularization of acute arterial mesenteric ischemia (AMI).

**Material and Methods:** Study of prospectively included patients with early arterial occlusive AMI who underwent endovascular revascularization. Follow-up CT were retrospectively reviewed to identify features of RI (bowel thickening, hyper-enhancement, fat stranding, intraluminal hemorrhage). Baseline clinical, laboratory, and imaging data along with procedures details were then compared between RI and non-RI patients. Outcomes were also compared.

**Results:** Fifty patients (23 men, median 72 yrs [interquartile range [IQR] 60–77]) were included, among which 22 experienced RI (44%) after a median delay of 28 hours (IQR 22–48) after revascularization. The ileum and the jejunum were involved in 16/22 (73%) and 11/22 patients (50%). Patients with RI presented with abdominal pain (50%), rectal bleeding (10%) and had elevated CRP serum level. In multivariable analysis, a decreased enhancement of the bowel wall (OR=69; CI 95% 5–3346) and an occlusion of the middle/distal part of the superior mesenteric artery (OR=24; CI 95% 1.4–957) were independent predictors of RI. The resection rate was higher in patients with RI (32% vs. 7% in non-RI patients; p=0.03). The 3-month survival rate of the cohort was 78% with no difference between RI and non-RI patients (log rank p=0.99).

**Conclusion:** Reperfusion injury is frequent after endovascular revascularization of early arterial occlusive AMI, especially in patients with a decreased bowel wall enhancement on pre-revascularization CT. It should not be mistaken for the recurrence of ischemia. Reperfusion injury does not seem to impair survival.

### SS 9.5

### Beyond chest: ischemic and hemorrhagic complications of the abdomen in COVID-19 patients

P.N. Franco[1], P. Bonaffini[1], A. Bonanomi[1], C. Valle[1], P. Marra[1], S. Sironi[2]; [1]*Bergamo/IT*, [2]*Milan/IT*

**Purpose:** To describe the occurrence of ischemic and/or hemorrhagic abdominal complications detected on contrast-enhanced computed tomography (CECT) scans in a series of COVID-19 patients and to correlate these manifestations with lung involvement, laboratory tests, comorbidities, and ongoing anticoagulant treatment.
**Material and Methods:** Thirty COVID-19 patients, undergone a CECT between March 16th and May 19th, 2020, for not lung-related clinical symptoms (i.e., abdominal pain), were retrospectively assessed. Ischemic and haemorrhagic complications were described and compared with lung involvement, blood coagulation tests, concomitant anticoagulant therapy, and comorbidities (PE). For lung involvement, a parenchymal stage was assigned.
**Results:** Ten patients showed ischemic complications (7 receiving anticoagulant therapy, 70%): 6/10 small bowel ischemia (1 concomitant obstruction, 1 perforation) and 4/10 ischemic colitis. Two patients with ischemia presented concurrent splenic infarctions and 1 patient also showed bilateral renal infarction. Main mesenteric vessels were patent except for 1 superior mesenteric vein thrombosis. Bleeding complications were found in 20 patients (all receiving some anticoagulant treatment): spontaneous hematomas in soft tissues (15), retroperitoneal hematomas (2) and gastro-intestinal bleeding (3). Ten presented active bleeding. Platelet and lymphocyte counts were within the normal range. D-dimer levels demonstrated a significant difference between the two groups, with higher values in ischemic cases (p<0.001). Most of the patients had severe lung involvement and 2 patients had evidence of PE.
**Conclusion:** Ischemic and bleeding abdominal complications may occur in COVID-19 patients, particularly associated with severe lung disease. CECT plays a crucial role in the diagnosis of these potentially life-treating conditions.

### SS 9.6

### Endovascular thrombectomy for the revascularization of acute occlusive arterial mesenteric ischemia: no benefit of mechanical over manual thrombectomy

L. Garzelli[1], I. Ben Abdallah[2], A. Nuzzo[1], J. Gregory[1], L. Paulatto[1], J.-C. Bijot[1], L. Raynaud[1], M. Dioguardi Burgio[2], V. Vilgrain[1], O. Corcos[1], Y. Castier[2], M. Ronot[1]; [1]*Clichy/FR*, [2]*Paris/FR*

**Purpose:** To evaluate and compare the performance of manual and mechanical endovascular thrombectomy for the revascularization of arterial occlusive acute mesenteric ischemia (AMI).
**Material and Methods:** Study of prospectively included patients with arterial occlusive AMI (January 2018–November 2021) who underwent endovascular thrombectomy. Baseline clinical, CT and procedural data were reviewed. Thrombectomy was either manual (aspiration with a 50-mL syringe) or mechanical using the Penumbra pump (Penumbra, Inc., Alameda, CA), at discretion of the operator. Complete and partial recanalization were evaluated along with procedural adverse effect. Post-procedural needs for bowel resection as well as morbidity (short bowel syndrome [SBS]) and 30-day survival were compared between thrombectomy methods.
**Results:** Thirty-one patients (19 female, median age 66 years (interquartile range [IQR] 55.5–75.5) were included: 15/31 and 16/31 were treated with mechanical and manual thrombectomy, respectively. Some 26/31 patients (84%) had a low preoperative probability of bowel necrosis (Clichy score 0–1) and clot was the cause in 24/31 patients (77%). Revascularization was performed a median 6.7 hours (IQR 5.7–12.7) after CT. Technical success was achieved in 11/15 (73%) and 12/14 (86%, two patients not evaluable) patients with mechanical and manual thrombectomy, with no difference between groups (p=0.41). Five patients (16%) had procedure-related complications. Secondary bowel resection was performed in 13/31 (42%) patients. Overall, 7/31 (23%) patients had SBS with no difference between groups (p=0.99), and 3-month survival rate was 60% and 81% in the mechanical and manual thrombectomy groups (log-rank p=0.13).
**Conclusion:** Endovascular thrombectomy in arterial occlusive AMI provided satisfactory early outcomes with no benefit of mechanical over manual methods.

### SS 9.8

### Added value of positive intraluminal contrast CT over fluoroscopic examination for detecting gastrointestinal leakage after gastrointestinal surgery

S.H. Kim, M.G. Kim, S.K. Jeon, S. Han; *Seoul/KR*

**Purpose:** To evaluate the added value of positive intraluminal contrast CT over fluoroscopy to detect anastomotic leakage after gastrointestinal (GI) tract surgery.
**Material and Methods:** We retrospectively included 141 GI tract surgery patients who underwent both fluoroscopic examinations and CT on the same day between January 2015 and February 2021. Two radiologists retrospectively reviewed the fluoroscopic images with and without CT. They were asked to determine anastomotic leakage on a 5-point confidence scale and grade the leakage on a 4-point grading system. Data on the duration of hospital stay and type of treatment were also collected from the electronic medical records. Radiologists' diagnostic performances for determining leakage were evaluated and compared using the ROC analysis, and interobserver agreement was further analyzed.
**Results:** In total, 53 patients had GI leakage. When CT images were added to fluoroscopic images, the AUC values significantly increased for both reviewers (from 0.859 to 0.942 for reviewer 1, P=0.001; from 0.757 to 0.879 for reviewer 2, P=0.002). Interobserver agreement between the two reviewers for the presence of leakage was excellent and improved with the addition of CT (weighted-kappa value, 0.869 versus 0.805). Postoperative intervention was more frequently performed (79.2% [42/53] versus 15.9%, [14/88]) (P<0.001) and the mean duration of postoperative hospital stay was significantly longer in patients with leakage than in those without (45 days versus 27 days) (P=0.003).
**Conclusion:** Positive intraluminal contrast CT has an added value over fluoroscopic examination for detecting GI leakage in GI tract surgery patients, increasing AUC values and improving interobserver agreement.

### SS 9.9

### Correlation of CT radiomic features for GISTs with the pathological classification and molecular subtypes: preliminary and monocentric experience

D. Palatresi, F. Fedeli, G. Danti, G. Grazzini, S. Pradella, V. Miele; *Florence/IT*

**Purpose:** Primarily to search for CT radiomics features of GISTs that could potentially correlate with the risk class according to Miettinen's classification, subsequently, to assess the existence of features with possible predictive value in differentiating responder from non-responder patients to first-line therapy with Imatinib.
**Material and Methods:** A retrospective study was used between June 2009 and December 2020. We analyzed the preoperative CT of patients undergoing surgery for gastrointestinal stromal tumors (GISTs). We segmented both non-contrast-enhanced CT (NCECT) and contrast-enhanced venous CT (CECT) images obtained either on three different CT scans (heterogeneous cohort) or on a single CT scan (homogeneous cohort). We then divided the patients into two groups according to Miettinen's classification and to the predictive value of response to Imatinib.
**Results:** We examined fifty-four patients with pathological confirmation of GISTs. For the heterogeneous cohort, we found a statistically significant relationship between 57 radiomic features for NCECT and 56 radiomic features for CECT with Miettinen's risk classification, while in the homogeneous cohort, we found 8 features for NCECT and 5 features for CECT, all included in the heterogeneous group. We also found some features for groups predictive of response to first-line therapy with Imatinib.
**Conclusion:** We found radiomic features that correlate with statistical significance for both the Miettinen risk class and the molecular subtypes of response. CT radiomic features may be useful in assessing the risk class and prognosis of GISTs. Our study has many inherent limitations, relating both to the small number of patients evaluated and to the monocentric and retrospective analysis.

### *SS 9.10*

### Reclassifying abdominal non-uterine leiomyosarcoma: using a radiological anatomic approach with implications for patient outcomes

U. Tarique[1], D. Cyr[1], C. Morosi[2], G. Greco[2], B. Dickson[1], R. Gladdy[1], D. Collegaro[2], A. Gronchi[2], C. Swallow[1], K. Khalili[1]; [1]*Toronto, ON/CA,* [2]*Milan/IT*

**Purpose:** We hypothesized that abdominal non-uterine leiomyosarcoma (ANU-LMS) disproportionately originates in veins downstream from sex-hormone producing organs (DSPO). Our purpose was to classify ANU-LMS using imaging and explore prognostic implications.

**Material and Methods:** Single-sarcoma center database was used. Inclusion criteria: biopsy-proven LMS of non-uterine abdominal/pelvic origin, pretreatment CT/MRI available for review. Exclusion criteria: uterine LMS and prior radiation. Imaging was reviewed by an abdominal sarcoma radiologist. LMS site of origin was assigned with a confidence rating (1–5). All tumors with low confidence ratings (1–2) were reviewed with a 2nd external expert radiologist by consensus. Tumors originating from the IVC were subclassified as below right gonadal vein (I); between gonadal vein and liver margin (IIA); between liver margin and hepatic veins (IIB); at/above hepatic vein (III). DSPO was defined as originating from ovarian/testicular vein, paratesticular, left renal vein, adrenal vein or IVC IIA.

**Results:** 152/225 patients (67.6%) were included. 92/152 (61%) were female. FNCLCC grade was I in 22/152 (15%), II in 61 (40%), and III in 49(32%). Distant metastases were found at presentation in 23/152 (15%). The top 5 site of origin were ovarian vein 24 (15.8%), IVC IIA 24 (15.8%), renal vein 11 (7.2%), paratesticular 11 (7.2%), and IVC IIB 8 (5.2%). Most common organ systems were veins 84/152 (55.3%), GI 24 (15.8%). genital 11 (7.2%), paratesticular 11 (7.2%). 84/152 (55.3%) of tumors were DSPO. On multivariable analysis, both size and DSPO were significant predictors of distant metastases at presentation (p=0.01) while sex, age, organ system/site and grade were not.

**Conclusion:** For both sexes, tumors arising DSPO constitute the majority of ANU-LMS and may impart a significantly lower risk of metastatic disease at presentation.

**09:00 - 10:30** **Auditorium 3 + 4**

### *Scientific Session Live SS 10*
### Hepatocellular carcinoma/liver function

### *SS 10.1*
### Sarcopenia impairs survival and treatment efficacy in patients with hepatocellular carcinoma undergoing immunotherapy

K. Lampichler, K. Pomej, L. Beer, L. Balcar, T. Meischl, C. Müller, M. Trauner, M. Scharitzer, D. Tamandl, B. Scheiner, M. Pinter; *Vienna/AT*

**Purpose:** Sarcopenia is a common problem in patients with hepatocellular carcinoma (HCC) and impairs the clinical course of these patients. The impact of sarcopenia on survival and treatment efficacy in HCC patients undergoing immunotherapy has yet to be evaluated.
**Material and Methods:** Patients with HCC treated with PD-(L)1-based immunotherapy between June 2016 and October 2021 were included. Sarcopenia was defined by transversal psoas muscle thickness (TPMT) at <12 mm/m in men and <8 mm/m in women at the level of the third lumbar vertebrae using cross-sectional imaging (CT/MRI) at baseline. We investigated the impact of sarcopenia on radiological response (mRECIST 1.1) as well as time-to-progression, progression-free survival and overall survival.
**Results:** Sarcopenia was present in 37/83 (45%) patients and associated with a worse radiological response compared to patients without sarcopenia: complete/partial response: 11.5% vs. 35.7%, stable disease: 30.8% vs. 40.5, progressive disease: 57.7% vs. 23.8% (p=0.011). Patients with sarcopenia also had a significantly worse outcome: Time-to-progression: 2.5 (95% CI: 1.9–3.1) vs. 10.4 (95% CI: 8.7–12.1) months (p=0.010); progression-free survival: 2.3 (95% CI: 1.8–2.7) vs. 9.2 (95% CI: 2.5–15.9) months (p=0.002); overall survival: 5.3 (95% CI: 2.5–8.0) vs. 22.6 (95% CI: 8.6–36.6) months (p=0.012). Even after multivariable adjustment for baseline Child–Pugh stage, ECOG PS >1, presence of macrovascular invasion or extrahepatic metastasis and baseline AFP levels (IU/mL), sarcopenia (aHR: 2.24, 95% CI: 1.17–4.31) remained an independent predictor of survival.
**Conclusion:** The presence of sarcopenia as determined by TPMT measurement prior to treatment initiation significantly impairs survival and treatment efficacy in HCC patients undergoing immunotherapy.

### *SS 10.2*
### Prognostic values of LI-RADS categories vs. histopathological features in patients with solitary HCC undergoing hepatic resection: a multicentric study

F. Matteini[1], R. Cannella[1], R. Sartoris[2], M. Dioguardi Burgio[3], S. Mulé[4], M. Wagner[3], J.-C. Nault[5], F. Cauchy[3], O. Scatton[3], A. Laurent[4], O. Seror[5], A. Luciani[4], V. Vilgrain[2], M. Ronot[2]; *[1]Palermo/IT, [2]Clichy/FR, [3]Paris/FR, [4]Créteil/FR, [5]Bobigny/FR*

**Purpose:** To assess the prognostic value of LI-RADS categories in patients with solitary hepatocellular carcinoma (HCC) undergoing hepatic resection.
**Material and Methods:** Multicenter retrospective study including patients with solitary resected HCC from three institutions with available preoperative contrast-enhanced CT and/or MRI. The LI-RADSv2018 category was evaluated by three independent readers. Histopathological features including tumor differentiation (WHO), micro- and macrovascular invasion, and satellite nodules were collected. The overall (OS) and recurrence-free survival (RFS) were evaluated using multivariable Cox–Mantel proportional hazards models, Kaplan–Meier method, and log-rank test.
**Results:** 377 patients were included (292 men, mean 62.0±9.2 years, mean tumor size 51±8 mm). CT and MRI exams of 312 and 305 patients were analyzed (240 underwent both). After a median follow-up of 46.0 (24.0-70.5) months, no survival differences were observed between tumors categorized as LR-3/4 or LR-5 on CT (OS, p=0.789, RFS, p=0.849) or on MRI (OS p=0.682, RFS p=0.803). However, OS and RFS were shorter in patients LR-M/TIV vs. LR-3/4/5 tumors on MRI (OS, p=0.004, RFS, p<0.001), as was RFS with LR-M/TIV vs. LR-3/4/5 tumors on CT (OS, p=0.149, RFS, p=0.011). On multivariate analysis considering CT, tumor grade and size were independently associated with OS (HR, 2.01, p<0.001 and HR, 1.06, p=0.010) and RFS (HR, 1.34, p=0.035 and HR, 1.05, p=0.013), but not LR categories. On MRI, however, LR-M/TIV and tumor grade were independently associated with OS (HR, 1.76, p=0.044 and HR, 1.65, p=0.008), while LR-M/TIV was independently associated with RFS (HR, 1.81, p=0.005).
**Conclusion:** The LR-M/TIV categories on MRI bear independent prognostic values in patients with solitary HCC undergoing resection.

### *SS 10.3*
### Hepatocellular carcinoma treated by stereotactic ablative radiotherapy: response assessment and imaging features on MRI

C. Corallo, A. Littlejohns, C. Podesta, R. Goody, N. Casanova, A. Scarsbrook, R. Albazaz; *Leeds/UK*

**Purpose:** Stereotactic ablative radiotherapy (SABR) is an emerging treatment for hepatocellular carcinoma (HCC). Few studies report MRI features of HCC after SABR. The aim was to evaluate MRI characteristics of treated lesions to better understand their behaviour.
**Material and Methods:** Consecutive adults with HCC undergoing SABR Jan 2017–Dec 2020 at a single institution were retrospectively identified. MRI features at baseline and up to 24 months post-treatment were assessed (size, arterial phase hyperenhancement (APHE), non-peripheral washout, enhancing capsule, diffusion restriction, internal fat).
**Results:** 69 HCCs treated in 44 patients (F:M 1:4; mean age 71 years, range 30–89 years); MRI follow-up in 63/69. Follow-up 56/63 to 3 months, 39/63 to 6 months, 16/63 to 12 months, 4/63 to 18 months, 1/63 to 24 months (mean 7.8 months). Mean baseline lesion size 25mm (range 7–58 mm), gradually reducing in 45/56, 17/39 and 5/16 lesions at 3, 6 and 12 months, respectively (mean reduction 11mm, range 2–38mm). Two lesions progressed. Pre-treatment 87% demonstrated APHE, 63% non-peripheral washout, 21% capsular enhancement, 73% diffusion restriction and 13% internal fat; 12 months post-treatment these features reduced by 63%, 49%, 13%, 50%, and 6%, respectively. No lesion at 18 or 24 months showed further change in size or signal. At 3 months, peri-lesional APHE, diffusion restriction and reduced uptake of liver-specific contrast was common, with gradual progression to atrophy and fibrosis.
**Conclusion:** MRI features of HCC treated by SABR evolve over months. Interpretation should be guided by baseline characteristics. Changes in surrounding liver should not be misdiagnosed as progression.

### SS 10.4

### Can CT perfusion imaging predict tumor response to antiangiogenetic treatment in HCC patients?

C. Maino, M. Ragusi, T. Giandola, R. Corso, C. Talei Franzesi; *Monza/IT*

**Purpose:** To investigate whether perfusion-CT (p-CT) imaging could depict the inhibition of tumour neoangiogenesis induced by Sorafenib in advanced hepatocellular carcinoma (HCC), and whether it could be useful in predicting survival during treatment.
**Material and Methods:** Ninety-eight p-CT examinations were performed among 29 cirrhotic patients, with advanced HCC before and every 2 months after Sorafenib administration, on a 256-slice MDCT scanner. Perfusion parameters were considered and statistically compared, at baseline and follow-up, between non-progressor (complete response, stable disease or partial response) and progressor (progressive disease) group. Kaplan–Meier analyses estimated the time-to-survival in overall population, after stratifying patients according to mRECIST.
**Results:** The responder group showed a significant reduction of perfusion values in HCC target lesions after anti-angiogenic therapy ($p \leq 0.01$), in comparison with progressor group which demonstrated an increase or no significant variation. When patients were stratified into mRECIST categories, higher survival rate was observed in the non-progressor group compared to the progressor (48.6% vs 28.6%), and statistically significant correlation (p=0.01) was found between percentage variation of perfusion parameters, from baseline to follow-up, and overall survival rate.
**Conclusion:** The quantitative assessment of perfusion parameters represents a prognostic indicator useful in the assessment of response to anti-angiogenic therapy, allowing for optimization of individualized treatment.

### SS 10.5

### Hepatocellular carcinoma in patients with non-alcoholic steatoheaptitis is associated with heterogeneous pattern of fat infiltration in skeletal muscles

M. Nachit[1], M. Dioguardi Burgio[2], A. Abyzov[2], P. Garteiser[2], V. Paradis[3], V. Vilgrain[3], I. Leclercq[1], B. Van Beers[3]; *[1]Brussels/BE, [2]Paris/FR, [3]Clichy/FR*

**Purpose:** The prevalence of hepatocellular carcinoma (HCC) in patients with non-alcoholic fatty liver disease (NAFLD) is alarmingly increasing. Tools to assess the risk for HCC development are needed to optimize and tailor surveillance in the growing NAFLD population. Here, we evaluated the association between fat infiltration in skeletal muscles and HCC in patients with NAFLD.
**Material and Methods:** In a cross-sectional cohort of 72 histologically-proven NAFLD patients amongst which 20 had HCC diagnosed on biopsy, we used proton density fat fraction (PDFF) at MRI to evaluate fat infiltration in muscles (mean fat concentration and first-order radiomic-based pattern) at the third lumbar level in erector spinae (ES), quadratus lumborum, psoas, oblique and rectus muscles.
**Results:** In patients with non-alcoholic steatohepatitis (NASH) and HCC, mean muscle PDFF was twice as high compared to patients with NASH but without HCC (quadratus lumborum: 6±4% versus 3±2%, ES 11±5% versus 5.4±3.1% and oblique muscle: 13±7% versus 8±5%, all $p < 0.05$). $ES_{PDFF}$ was an independent and significant predictor of HCC in patients with NASH in multivariate models (all $p < 0.05$). Energy and entropy (radiomic features for heterogeneity) in ES were strong and independent predictors of HCC in patients with NASH (AUC = 0.92 and 0.88, respectively, $p < 0.001$).
**Conclusion:** The results of our study support that, in patients with NASH, HCC is associated with heterogeneous accumulation of fat in skeletal muscles.

### SS 10.6

### Combined functional liver imaging score derived from gadoxetic acid-enhanced MRI and spleen size as predictors of mortality and hepatic decompensation in chronic liver disease

N. Bastati-Huber, L. Beer, S. Poetter-Lang, T. Reiberger, M. Mandorfer, A. Ba-Ssalamah; *Vienna/AT*

**Purpose:** To investigate the accuracy of the functional liver imaging score (FLIS) plus spleen–craniocaudal diameter (SCCD) for predicting hepatic decompensation and transplant-free survival (TFS) in patients with chronic liver disease (CLD).
**Material and Methods:** FLIS derived from hepatobiliary phase (HBP) MRI was associated with graft survival in patients who underwent liver transplantation and TFS in patients with advanced chronic liver disease (ACLD). 397 patients with CLD who had undergone gadoxetic acid-enhanced liver MRI were included. A FLIS was assigned based on the sum of three HBP features: hepatic enhancement, biliary excretion, and the signal intensity in the portal vein. The SCCD was measured. Patients were stratified into three groups: non-advanced CLD, compensated-advanced CLD (cACLD), and decompensated-advanced CLD (dACLD). The predictive value of SCCD and FLIS for first hepatic decompensation and TFS was investigated.
**Results:** We observed a strong positive correlation between the measured spleen volume and the SCCD (Spearman's rho=0.887; P<0.001). In patients with ACLD, the FLIS was an independent risk factor for mortality (adjusted-hazard ratio, [aHR]: 2.38, 95%CI: 1.51–43.76, P<.001). Allocating patients into three groups based on their FLIS and SCCD enabled to further stratify patients according to their risk for mortality (log-rank-test: P<.001). The SCCD was further identified as an independent risk factor for first and further hepatic decompensation in patients with ACLD (aHR: 1.10, 95%CI: 1.02–1.19, P=.01; aHR: 1.13, 95%CI: 1.05–1.22, P=.001).
**Conclusion:** The functional liver imaging score derived from gadoxetic acid-enhanced MRI in combination with the SCCD identifies patients with advanced chronic liver disease who are at increased risk for a hepatic decompensation and for mortality.

### SS 10.7

### Automated liver segmental volume ratio quantification on non-contrast T1–VIBE Dixon liver MRI using an artificial neural network

L. Zbinden[1], D. Catucci[2], Y. Suter[1], A. Berzigotti[1], L. Ebner[1], A. Christe[1], V. Obmann[1], R. Sznitman[1], A. Huber[1]; *[1]Bern/CH, [2]Herrenschwanden/CH*

**Purpose:** To use an artificial neural network for automated liver segmental volume quantification and calculation of the liver segmental volume ratio (LSVR) on a non-contrast T1-volumetric interpolated breath-hold examination (VIBE) Dixon liver MRI.
**Material and Methods:** A dataset of 30 liver MRI with a non-contrast 3mm T1-VIBE Dixon sequence was manually labelled slice-by-slice by an expert for Couinaud liver segments by excluding the liver vessels. A state-of-the-art neural network for segmentation (3D U-Net) was trained with nested cross-validation using 27 liver MRI for training and 3 unseen by the neural network for validation in 10 distinct iterations. Liver segmental volumes were retrieved and the LSVR was calculated as the liver segment volumes I–III divided by the liver segmental volumes IV–VIII. The LSVR was compared with the expert manual LSVR calculation and the LSVR calculated on CT scans in 30 patients with CT and MRI within 3 months.
**Results:** The neural network model classified the Couinaud segments I–VIII with an average Dice score of 0.702±0.08, ranging between 0.676±0.10 (segment VI) and 0.763±0.06 (segment III). The calculated mean LSVR with 30 liver MRI unseen by the model was 0.328±0.13, as compared with manually quantified LSVR of 0.344±0.16, resulting in a mean absolute error (MAE) of 0.036. A comparable LSVR of 0.352±0.15 with a MAE of 0.044 resulted with the LSRV retrieved from the CT scans in the same patients. By the Kruskal–Wallis test, the sample differences are not statistically significant (p=.863).
**Conclusion:** A state-of-the-art artificial neural network allows automated liver segmental volume quantification and accurate calculation of LSVR based on a non-contrast T1-VIBE Dixon sequence.

### SS 10.8

### CT-based volumetry as complement to liver scintigraphy for the follow-up of auxiliary partial orthotopic liver transplantation

A. Kirchner[1], M. Ronot[1], F. Dondero[1], F. Durand[1], M. Lesurtel[1], R. Lebtahi[1], V. Vilgrain[1], M. Dioguardi Burgio[2]; [1]*Clichy/FR*, [2]*Paris/FR*

**Purpose:** To assess whether CT volumetry can be used to monitor liver recovery in patients who underwent auxiliary partial orthotopic liver transplantation (APOLT) for acute liver failure (ALF), and to compare it to scintigraphy with 99mTC-trimethyl-Br-IDA tracer (TBIDA scintigraphy).
**Material and Methods:** Retrospective cohort study of all patients who underwent APOLT (October 2006 to July 2019) in our institution. Collected data included liver CT volumetry measurements, TBIDA scintigraphy results, biological and clinical data including immunosuppression therapy after APOLT. Four follow-up time-points were defined (baseline, discontinuation of mycophenolate mofetil (MMF), beginning of tacrolimus reduction and tacrolimus discontinuation) for analysis.
**Results:** 24 patients (7 males; median age 28.5 years) were included. Main etiologies of ALF were acetaminophen intoxication (n=12), hepatitis B virus (n=5), and *Amanita phalloides* intoxication (n=3). Median native liver function fractions on scintigraphy were 22.0% (IQR 14.0–30.8), 30.5% (21.5–49.0), 32.0% (28.0–59.25) and 93.0% (75.6–100.0) at baseline, discontinuation of MMF, tacrolimus reduction and tacrolimus discontinuation, respectively. The corresponding median native liver volume fractions in CT were 12.8% (10.4–17.3), 20.5% (14.2–27.3), 24.7% (21.3–48.4) and 77.9% (62.5–96.9). Volumes and functions were strongly correlated (r = 0.894, 95% CI 0.844–0.929, p<0.01). Median time-to-immunosuppression discontinuation was 25.0 (17.0–35.0) months. Estimated time-to-immunosuppression discontinuation was shorter in patient with acetaminophen-associated ALF (22 vs. 35 months; p=0.035).
**Conclusion:** CT-based liver volumetry closely parallels function recovery, and could be used as complement to TBIDA to monitor the recovery of the native liver in patients undergoing APOLT for ALF.

### SS 10.9

### Evaluation of liver injury with MRI after chemotherapy

L. Tomaiuolo, L. Geraci, C. Longo, M. Todesco, D. Autelitano, A. Carli, F. Cicalo', F. Moro, R. De Robertis, N. Cardobi, M. D'Onofrio; *Verona/IT*

**Purpose:** The aim of the study is to evaluate long-term liver injuries as sinusoidal obstruction syndrome (SOS) and chemotherapy-associated steatohepatitis (CASH) with functional MRI in patients with pancreatic ductal adenocarcinoma (PDAC) after neoadjuvant chemotherapy.
**Material and Methods:** In this prospective study, 10 patients with borderline resectable/locally advanced PDAC underwent a MRI at 1.5T including gradient echo sequence (T2-FFE) before and after 12 weeks from the beginning of neoadjuvant chemotherapy (liposomal irinotecan + fluorouracil/leucovorin + oxaliplatin). Clinical parameters as body mass index, history of diabetes, hypertension and biological parameters including bilirubin levels, AST, ALT, GGT and drug administration dosage were recorded. A single region of interest (ROI) including the whole liver and other 10 ROIs for hepatic segments were reproduced obtaining a T2* mapping for each patient before and after treatment. T2* values were measured and compared with Wilcoxon test.
**Results:** Significant decrease of T2* values was observed after treatment (p < 0.05), demonstrating a lower hepatic oxygenation, especially for II, VII and VIII hepatic segments. For each patient, III segment T2* values were stable. In 3 patients, no significant changes of T2* values were recorded before and after chemotherapy.
**Conclusion:** Liver steatosis and fibrosis interpose with sinusoidal patency. Including T2* mapping in liver MRI protocol may supply estimation of liver functional changes after chemotherapy.

### SS 10.10

### Quantitative MRI in the evaluation of patients with non-alcoholic steatohepatitis

F. Pucciarelli, M. Zerunian, M. Polici, B. Masci, E. Iannicelli, D. Caruso, A. Laghi; *Rome/IT*

**Purpose:** To evaluate the reliability of quantitative magnetic resonance imaging (MRI) in the diagnosis and follow-up of patients with non-alcoholic steatohepatitis (NASH).
**Material and Methods:** From March to September 2021, twenty patients who met diagnostic criteria for NASH and twenty healthy volunteers were prospectively enrolled and underwent quantitative 1.5T MRI examination of the liver. Acquisition protocol comprised magnetic resonance elastography (MRE) and proton density fat fraction (PDFF). Quantitative image analysis was performed by a radiologist with 15 years of experience in abdominal MRI, on a dedicated workstation; liver stiffness (kPa) and grade of steatosis (%) were collected. Statistical analysis was performed using a dedicated software and a p value <0.05 was considered significant.
**Results:** Liver stiffness resulted higher in patients with NASH than healthy volunteers (10.8±6.42kPa vs 4.75±1.44kPa; p=0.0002). PDFF was higher in patients with NASH than healthy volunteers (2.52±0.56% vs 1.87±0.26%; p=0.0020). Steatosis was present in 100% of the NASH group patients, and in 15% of the healthy volunteers; the NASH group showed elevated liver stiffness in 80% of patients (0% in healthy volunteers). Quantitative MRI performance for liver stiffness and PDFF detection showed an area under the curve (AUC) of 0.915 (sensitivity 100%; specificity 75%) and 0.843 (sensitivity 60%; specificity 90%), respectively. The whole time examination was 72 seconds (55 seconds for MRE and 17 seconds for PDFF).
**Conclusion:** Quantitative MRI techniques are reliable in the study of NASH. Furthermore, being very fast and risk-free protocols, these techniques could also be used as screening methods in high-risk population at risk of developing diffuse liver disease.

**09:00 - 10:30** **Auditorium 8**

*Scientific Session Live SS 11*

### Rectal cancer/colorectal liver metastases

### SS 11.1

### Multireader comparison and validation of previously published MRI tumour response evaluation methods in rectal cancer

N. El Khababi[1], M.J. Lahaye[1], M. Maas[1], S. Nougaret[2], L. Curvo-Semedo[3], R. Tissier[1], N. Bogveradze[4], N.W. Schurink[1], J. van Griethuysen[1], R.G.H. Beets-Tan[1], D. Lambregts[1], +. Multicenter MRI rectal study group[1];
*[1]Amsterdam/NL, [2]Montpellier/FR, [3]Coimbra/PT, [4]Tbilisi/GE*

**Purpose:** To compare four previously published methods for rectal tumor response evaluation after chemoradiotherapy (CRT) in a multicenter study setting among readers with different experience levels.
**Material and Methods:** 22 international radiologists (5 rectal MR-experts, 17 general/abdominal radiologists) retrospectively reviewed the post-CRT MRIs of n=90 patients (from 10 centers), using four previously published response methods. Two based on T2W-MRI only: (1) mr-tumor-regression-grade (mrTRG) and (2) split-scar sign (Santiago et al. 2020). Two based on T2W-MRI + DWI: (3) modified-mrTRG, and (4) DWI-pattern approach (Lambregts et al. 2018). Mixed model linear regression was used to calculate average sensitivity/specificity/accuracy to predict a complete response (vs residual tumor) post-CRT and to assess the impact of reader experience level. Interobserver agreement (IOA) was calculated using Kendall's coefficient (W). Readers were asked to indicate their preferred scoring method(s).
**Results:** Average sensitivity/specificity/accuracy to predict a complete response were 52%/82%/73% (mrTRG), 25%/94%/74% (split-scar), 28%/95%/75% (modified-mrTRG), and 30%/96%/77% (DWI-patterns), respectively. Performance was significantly positively affected by higher reader experience level. Effect size was significantly negative (lower accuracy and specificity) for mrTRG compared to the other methods, though with a positive effect size for sensitivity. IOA was W0.48 (mrTRG), W0.25 (split scar), W0.46 (modified-mrTRG), W0.51 (DWI-patterns). DWI-patterns were chosen as the preferred scoring method by 73% (vs. 36% modified-mrTRG, 18% mrTRG, 5% split scar).
**Conclusion:** mrTRG showed significantly lower accuracy and specificity (but higher sensitivity) to diagnose complete responders. DWI-based methods achieved the best results based on a combination of IOA, reader preference and highest specificity to detect patients with residual tumor. Higher reader experience positively affected diagnostic performance.

### SS 11.2

### Impact of MR image quality on diagnostic performance to assess response after chemoradiotherapy in rectal cancer

N. El Khababi[1], M. Maas[1], M.J. Lahaye[1], S. Nougaret[2], L. Curvo-Semedo[3], R. Tissier[1], N. Bogveradze[4], N.W. Schurink[1], J. van Griethuysen[1], R.G.H. Beets-Tan[1], D. Lambregts[1], +. Multicenter MRI rectal study group[1];
*[1]Amsterdam/NL, [2]Montpellier/FR, [3]Coimbra/PT, [4]Tbilisi/GE*

**Purpose:** To assess the impact of MR image quality on diagnostic performance to assess rectal tumor response after chemoradiotherapy (CRT).
**Material and Methods:** 22 radiologists assessed response to CRT in a multicenter patient group of n=90 (from 10 centers with varying acquisition protocols) on post-CRT T2W-MRI (using the mr-tumor-regression-grade (mrTRG)) and a combination of T2W-MRI+DWI (using a modified-mrTRG incorporating DWI-findings), respectively. Impact of image quality on average sensitivity/specificity/NPV/PPV/accuracy to diagnose a complete response (versus residual tumor) was calculated using mixed model linear regression. Image quality was graded for each MRI with a 0–6-point score designed for the purpose of this study based on current guidelines. The score was composed of the following criteria: for T2W-MRI (1) slice-thickness ≤3mm, (2) in-plane-resolution ≤0.6x0.6mm, (3) angulation perpendicular to tumor-axis; for DWI (4) high b value ≥b800, (5) sufficient signal-to-noise ratio, and (6) absence of significant artefacts. A total score of ≤3 was classified as below-standard quality.
**Results:** Quality of 38/90 (42%) cases was classified as below standard, which had a significant negative impact on the readers' average specificity (effect size −0.096, P<0.001) and overall accuracy (effect size −0.045, P<0.001), with similar effects for the mrTRG (T2W-MRI) and modified-mrTRG (T2W-MRI+DWI) scorings. Average overall accuracy was 72% (mrTRG/T2W-MRI) and 77% (modified-mrTRG/ T2W-MRI+DWI) for the good-quality scans versus 67% (mrTRG/T2W-MRI) and 72% (modified-mrTRG/ T2W-MRI+DWI) for the below-standard quality scans.
**Conclusion:** MR image quality has a significant effect on radiologist's diagnostic performance to assess response of rectal tumours to chemoradiotherapy on restaging MRI. Optimizing image acquisition is, therefore, crucial to ensure optimal diagnostic performance.

### SS 11.3

### Predicting response to chemoradiotherapy in rectal cancer via visual assessment on baseline staging MRI: a multicenter and multireader study

N. El Khababi[1], M.J. Lahaye[1], M. Maas[1], S. Nougaret[2], L. Curvo-Semedo[3], N. Bogveradze[4], N.W. Schurink[1], J. van Griethuysen[1], R.G.H. Beets-Tan[1], D. Lambregts[1], +. Multicenter MRI rectal study group[1]; [1]*Amsterdam/NL,* [2]*Montpellier/FR,* [3]*Coimbra/PT,* [4]*Tbilisi/GE*

**Purpose:** van Griethuysen et al. (Abdom Radiol2020) proposed a 5-point confidence score to predict response to chemoradiotherapy (CRT) on baseline MRI based on visual morphologic assessment/staging. Aim was to test this scoring system in a multicenter setting among multiple readers with different experience levels, and compare it to an alternative 4-point risk-score, designed for the purpose of this study.

**Material and Methods:** 22 international radiologist (5 rectal MR experts, 17 general/abdominal radiologists) retrospectively reviewed the pre-CRT MRIs of n=90 patients (from 10 centers) to estimate the chance that patients would achieve a complete response (CR). Readers first applied the 5-point score of van Griethuysen. They then assigned a risk score with 1-point for each of the following features: bulky (cT3cd-4) tumour, obvious mesorectal fascia invasion, obvious nodal involvement, obvious extramural vascular invasion. Diagnostic performance to predict CR was assessed using ROC curves; interobserver agreement (IOA) was calculated using Kendall's coefficient (W). Readers were asked to indicate their preferred scoring method.

**Results:** Mean (+range) area under the ROC curve (AUC) for the 5-point confidence score was 0.67 (0.60–0.76) for the MR experts and 0.61 (0.52–0.73) for the abdominal/general radiologists; for the 4-point risk-score AUCs were 0.68 (0.62–0.80) and 0.62 (0.52–0.70), respectively. IOA was similar for both methods (W0.65 for the confidence score; W0.66 for the 4-point risk score), but higher for the MR experts (W0.76 and W0.78, respectively). Most readers (55%) favored the 4-point risk score.

**Conclusion:** Diagnostic performance to predict a complete response was moderate with similar results for the 5-point confidence score and 4-point risk-score. Diagnostic performance and IOA were better for the more experienced readers.

### SS 11.4

### The definition of a near-complete response after neoadjuvant (chemo)radiotherapy for rectal cancer: results of an expert survey

P. Custers, G.L. Beets, D. van der Reijd, M. van Leerdam, B. van Triest, D. Lambregts, M. Maas; *Amsterdam/NL*

**Purpose:** A variety of definitions is used for a near-complete response (nCR) following (chemo)radiotherapy for rectal cancer. This definition is important to guide treatment decision-making when considering patients for organ preservation. The aim of this study is to achieve an international consensus-based definition of nCR.

**Material and Methods:** An online survey on the definition of a nCR including 21 image features derived from endoscopy, T2W-MRI and DWI, 20 statements and 20 cases of rectal cancer patients 8–12 weeks following (chemo)radiotherapy was performed. Ten clinical experts (7 surgeons, 3 radiologists) took part. Consensus was defined as ≥80% agreement.

**Results:** Consensus was established in 38% of features, 35% of statements, and 30% of cases. Features that were agreed upon as indicative of a nCR were small flat ulcer on endoscopy (86% agreement) and focal spots of high signal on DWI (80% agreement). Agreement was reached on the following statements: (1) response on endoscopy is more decisive than response on MRI; (2) biopsies are not always needed if a nCR is found; (3) besides luminal response, nodal status should be included when defining nCR. The following cases were deemed a nCR: (1) a flat white scar, homogeneous hypo-intense fibrosis, and focal diffusion signal; (2) a flat white scar, heterogeneous irregular fibrosis, and linear focal signal on DWI; (3) a small flat ulcer, heterogeneous irregular fibrosis, and no high DWI signal.

**Conclusion:** These preliminary results are the first step towards a consensus-based definition of a nCR, aiming on more uniformity regarding selection of patients for organ-preservation.

### SS 11.5

### MRI assessment of rectal cancer response to neoadjuvant therapy: a multireader study

J.B. Yuval[1], S. Patil[1], N. Gangai[1], D.G. Akselrod[2], A. Fung[3], C.B. Harmath[4], R. Kampalath[5], K. Krehbiel[6], S. Lee[5], P.S. Liu[7], J.D. Millet[8], R.B. O'Malley[9], A.S. Purysko[7], J.C. Veniero[7], A.P. Wasnik[8], J. Garcia-Aguilar[1], M.J. Gollub[1]; [1]*New York, NY/US,* [2]*Burlington, VT/US,* [3]*Portland, OR/US,* [4]*Chicago, IL/US,* [5]*Irvine, CA/US,* [6]*Omaha, NE/US,* [7]*Cleveland, OH/US,* [8]*Ann Arbor, MI/US,* [9]*Seattle, WA/US*

**Purpose:** A watch and wait strategy with the goal of organ preservation is an emerging treatment paradigm for rectal cancer following neoadjuvant treatment. Selection of appropriate patients remains a challenge. Most previous efforts to measure the accuracy of MRI in assessing rectal cancer response used a small number of radiologists and did not report variability among them. Our aims were to measure the accuracy and describe the interobserver variability of interpretation of rectal cancer response between radiologists at different medical centers.

**Material and Methods:** Twelve radiologists from 8 institutions assessed baseline and restaging MRI scans of 39 patients. The participating radiologists were asked to assess MRI features and to categorize the overall response as complete or incomplete. The reference standard was pathological complete response or a sustained clinical response for >2 years.

**Results:** Overall accuracy was 64%, with a sensitivity of 65% for detecting complete response and specificity of 63% for detecting residual tumor. Interpretation of the overall response was more accurate than the interpretation of any individual feature. Variability of interpretation was dependent on the patient and feature investigated. Low variability of interpretation was associated with high accuracy of interpretation.

**Conclusion:** MRI-based evaluation of response at restaging is insufficiently accurate and has substantial variability of interpretation. Although some patients' response to neoadjuvant treatment on MRI may be easily recognizable, as seen by high accuracy and low variability, that is not the case for most patients.

### SS 11.6

### Role of MRI for early tumor response evaluation in anal cancer after 5 weeks of chemoradiotherapy to guide boost strategies

D. Lambregts, E. van den Hurk, F. Peters, F.E. Voncken, B.A. Grotenhuis, C.L. Deijen, B. van Triest; *Amsterdam/NL*

**Purpose:** Standard treatment for ≥cT2 and/or N+ anal cancer is chemoradiotherapy. At our institution, a sequential radiotherapy boost (3x1.8Gy) is given selectively to patients showing insufficient response after 5-week chemoradiotherapy. Aim was to assess the impact of MRI (including T2-weighted and diffusion-weighted sequences) in addition to digital rectal examination (DRE) to guide this decision.

**Material and Methods:** Retrospective analysis of 48 patients treated with chemoradiotherapy±boost. On DRE, response at week 5 was classified as insufficient when an obvious residual mass was palpable. On MRI, response was classified as insufficient in case of predominant residual mass (intermediate-T2W-/high-DWI signal) and sufficient in case of predominant fibrosis with no/minor DWI signal. The decision whether to boost was guided by the findings of DRE+MRI.

**Results:** Scenario-A: In 16/48 patients (33%), DRE was not feasible because of pain. In 8/16, MRI indicated insufficient response (88% received boost), in 7/16, MRI indicated sufficient response (boost omitted in 86%), in 1/16, MRI was inconclusive. Scenario-B: In 21/48 patients (44%), MRI confirmed DRE findings and in 81% boost strategies were planned accordingly; in the remaining 19%, alternative strategies were guided by patient preference/comorbidities. Scenario-C: in 11/48 patients (23%), MRI contradicted DRE findings; these were mostly (8/11) cases where DRE suspected residual tumor, but MRI indicated sufficient response. Boost was omitted in the majority (75%).

**Conclusion:** Addition of MRI to DRE seems helpful to assess response during CRT and guide the decision whether or not to give a sequential radiotherapy boost, in specific when DRE is not feasible (±1/3) and in cases where DRE underestimates the response.

### SS 11.7

### Intraindividual comparison of diagnostic efficacy of orally administered liver-specific contrast agent Mangoral (Orviglance) (manganese chloride tetrahydrate) and intravenous gadobenate dimeglumine (Multihance) in patients with colorectal liver metastases

K. Shamsi[1], T. Brismar[2], N. Kartalis[2], C. Bjartmar[3];
*[1]Conshohocken, PA/US, [2]Stockholm/SE, [3]Malmö/SE*

**Purpose:** To compare the diagnostic efficacy of Mangoral-enhanced MRI (Mn-MRI) with gadobenate dimeglumine-enhanced MRI (Gd-MRI)

**Material and Methods:** Twenty patients with colorectal liver metastases were included in a single-center phase II study. All patients underwent both Mn-MRI and Gd-MRI. Efficacy of Mn-MRI and Gd-MRI was assessed by 3 independent radiologists (R1, R2, R3) in terms of number and size of the lesions, border delineation and lesion contrast using a 4-point scale, and quantitative parameters (signal-to-noise ratio (SNR) and lesion-to-liver contrast (LLC)). Descriptive statistics and confidence interval (CI) were used to assess the differences.

**Results:** A higher number of liver metastases were detected by Mn-MRI compared to Gd-MRI for all 3 readers (mean lesion number: R1: 2.15 vs 1.60; R2: 2.40 vs 2.15; 3.00 vs 2.65 for Mn-MRI and Gd-MRI, respectively, with overlapping CIs). Compared to unenhanced MRI, both Mn-MRI and Gd-MRI detected smaller lesions (average mean diameter for Mn-MRI 15.49 mm vs 17.01 for unenhanced MRI; mean diameter for Gd-MRI 15.07 mm vs 18.3 mm for unenhanced MRI). For border delineation, the scores for Mn-MRI ranged from 6.40 to 9.37 compared to 4.41 to 7.79 for GMRI. For lesion contrast, two out of three readers had higher scores for Mn-MRI compared to the liver with overlapping CIs (score range for Mn-MRI vs. Gd-MRI: 6.80–10.84 vs. 5.18–8.74)). SNR/LLC was 95/1.97 for Mn-MRI and 96/1.65 for Gd-MRI, respectively.

**Conclusion:** Orally administered Mn-MRI showed similar efficacy in terms of lesion detection, lesion visualization and lesion delineation of liver metastases as compared to intravenous Gd-MRI.

### SS 11.8

### Impact of liver MRI in addition to CT on the local treatment plan of patients with colorectal liver metastases: a systematic review and meta-analysis

B. Görgec[1], I.M. Verpalen[1], M. Abu Hilal[1], S. Bipat[1], C. Verhoef[2], R.J. Swijnenburg[1], M. Besselink[1], J. Stoker[1];
*[1]Amsterdam/NL, [2]Rotterdam/NL*

**Purpose:** Computed tomography (CT) is the standard imaging method in the diagnostic workup of patients with colorectal liver metastases (CRLM). Compared to CT, magnetic resonance imaging (MRI) is superior in detection and characterization of CRLM, but there are limited data with regard to its effect on patient management. This meta-analysis summarizes studies with data on the impact of liver-MRI in addition to CT on the local treatment plan in the staging of CRLM.

**Material and Methods:** Searches of PubMed, EMBASE, and Cochrane Library databases were performed through January 2021. Studies concerning change in local treatment plan between CT findings solely and CT and liver-MRI findings combined in patients with CRLM were included. Pooled weighted proportions for the primary outcome were calculated using random effect meta-analysis.

**Results:** Ten studies (n=1413) were included that reported on possible change in local treatment plan in 450 patients (31.8%). Nine studies used a contrast-enhanced liver-MRI with eight studies administering gadoxetic acid. Liver-MRI with DWI was used in eight studies. Pooling of the results of all studies showed that 24.4% (95% CI 15.70–34.40%) of the patients had a change in local treatment plan based on the liver-MRI findings. Sensitivity analysis of the five studies (n=268) focusing on monophase portal venous CT and gadoxetic acid-enhanced liver-MRI with DWI showed a change of local treatment plan of 17.9% (95% CI 7.69–31.24%).

**Conclusion:** Additional liver-MRI changes local treatment plan in almost one fourth of the patients and implies significant clinical added value for liver-MRI in the staging of CRLM.

### SS 11.9

### Imaging characteristics of colorectal liver metastases before and after chemotherapy on gadoxetic acid-enhanced MRI

D. Van der Reijd, E. Soykan, B. Heeres, D. Lambregts, T. Buffart, K. Kuhlmann, R.G.H. Beets-Tan, M. Maas, E. Klompenhouwer; *Amsterdam/NL*

**Purpose:** To investigate to what extent colorectal liver metastases (CRLM) show typical imaging characteristics, such as arterial rim enhancement, moderate hyperintensity to liver on T2W-MRI, diffusion restriction and non-enhancement in hepatobiliary phase, on gadoxetic acid-enhanced MRI before and after chemotherapy.

**Material and Methods:** From November 2015 until February 2021, we retrospectively identified 247 patients with gadoxetic acid-enhanced MRI before and/or after chemotherapy for pathologically proven CRLM. 664 CRLM were included, with a maximum of 5 CRLM per patient. MRIs with low image quality and mucinous CRLM were excluded. MRI pre-chemotherapy was available in 654 CRLM (n=157 patients), MRI post-chemotherapy in 160 CRLM (n=49 patients) and both pre- and post-therapy MRI in 150 CRLM (n=41 patients). Two expert radiologists assessed size, location, enhancement patterns, signal intensity on multiple sequences, and hyperintensity on DWI.

**Results:** On pre-chemotherapy MRI, only 58% of CRLM showed peripheral rim enhancement, the others appeared homogenous (30%) or heterogeneous (12%) on arterial and portal-venous phase. On T2W-MRI, CRLM presented moderately hyperintense (94%), isointense (5%) and severely hyperintense (1%). Diffusion restriction was found in 87% of CRLM. On post-chemotherapy MRI, CRLM showed similar patterns on contrast series with peripheral enhancement (47%), homogeneous (32%) or heterogeneous (8%) appearance, or disappeared (13%). On T2W-MRI, CRLM presented less often moderately hyperintense (76%) and more often isointense (24%). Post-chemotherapy, diffusion restriction was found in 60% of CRLM. All CRLM (100%) were non-enhancing on hepatobiliary phase, both before and after chemotherapy.

**Conclusion:** CRLM do not always show typical imaging characteristics on gadoxetic acid-enhanced MRI like arterial rim enhancement and diffusion restriction, especially after chemotherapy.

### SS 11.10

### External validation of a CT-based radiomics model for the prediction of local tumour progression after ablation in colorectal liver metastases

D. Van der Reijd[1], F. Staal[1], C. Guerendel[1], M. Busard[1], S. Benson[1], M. Taghavi[1], S. Roberti[1], E. Klompenhouwer[1], A. Moelker[2], C. Verhoef[2], M. Starmans[2], R.G.H. Beets-Tan[1], M. Maas[1]; *[1]Amsterdam/NL, [2]Rotterdam/NL*

**Purpose:** After thermal ablation (TA) of colorectal liver metastases (CRLM), local tumour progression (LTP) is reported in 6–46%. Early identification of patients at risk for LTP may avoid delay in additional treatment. We aimed to validate a previously published clinical-radiomics model (c statistic: 0.78; 95%CI: 0.58–0.84) to identify patients at risk for LTP in an independent external cohort.

**Material and Methods:** 53 patients (n=79 CRLM) treated with TA were retrospectively included. Clinical features were collected. Portal-venous CT images 2–8 weeks after TA were used for segmentation. Radiomics features (with different Laplacian of Gaussian (LoG) filters) as used in the previous model were extracted from the ablation zone (AZ) and a 10 mm periablational rim (PAR). The three multivariable stepwise Cox regression models (clinical-only, radiomics-only, clinical-radiomics combined) were applied on the data and performances (concordance [c] statistics) were compared to the previous results.

**Results:** After a median follow-up of 26 months (range 6–149), LTP occurred in 25 ablation zones (32%). Both the clinical-only and radiomics-only models demonstrated poor predictive performance with c statistics 0.53 (95%CI: 0.41–0.65) and 0.42 (95%CI: 0.31–0.54), respectively. The combined model included T-stage, metastasis size, adjuvant chemotherapy treatment, AZ_Uniformity_LoG-1.5, AZ_Skewness, PAR_Mean_LoG-0.5, PAR_Skewness_LoG-0.5 and PAR_Uniformity_LoG-1.5, and yielded a poor predictive performance, with c statistic 0.52 (95%CI: 0.40–0.61).

**Conclusion:** Previously published clinical and radiomics models could not predict LTP in CRLM after TA in an independent external cohort. This inability to reproduce the earlier findings could be explained by overtraining and scanner differences. These results underline the importance of external validation. Single-center radiomics models could be hospital specific and currently prohibit clinical use.

**11:00 - 12:30** **Auditorium 3 + 4**

*Scientific Session Live SS 12*

### Peritoneum/technique

### SS 12.1

### The value of providing a second read of external imaging for patients referred to a specialist peritoneal malignancy centre

J. El-Sheikha, A. Bhagwanani, O. Duxbury, D. Patel, N. Shah, A. Thrower; *Basingstoke/UK*

**Purpose:** To investigate the value in providing a second read of externally obtained imaging for patients referred to a tertiary centre specialising in pseudomyxoma peritonei (PMP) and colorectal peritoneal metastases (CPM).

**Material and Methods:** Between 1st September 2019 and 1st September 2021, all patients referred with PMP and CPM with images second read were analysed, with external reports graded on a four-point discrepancy scale; 0=no local report available, 1=agree, 2=minor disagreement, and 3=significant disagreement.

**Results:** Of 1967 eligible reports, 76.8% (n=1512) were referred with PMP and 23.1% (n=455) were referred with CPM. The overall median age was 60 (i.q.r 51–70) with a prominence of females referred with PMP (female proportion; PMP 64.2% vs CPM 49% P<0.001). Most second reads were in patients which had been previously reviewed (86.5% n=1702), with a minority of new referrals (13.5% n=265). Second read agreement was greater overall with PMP referrals (PMP n=1105 (73.1%) vs CPM n=295 (64.8%), p<0.001). Disagreement was greater overall among CPM referrals, in both the minor (PMP n=270 (17.9%) vs CPM n=104 (22.9%), p=0.011) and major discrepancy categories (PMP n=81 (5.4%) vs CPM n=42 (9.2%) p=0.003. 70 reports did not have a local report provided (PMP n=56 (3.7%) vs CPM n=14 (3.0%), p=0.319).

**Conclusion:** There is a significant value in providing a second read of externally obtained imaging for patients referred to a specialist tertiary centre for PMP or CPM, with a substantial proportion of reports in disagreement and a significant minority with severe discrepancies.

### SS 12.2

### FDG-PET/MRI for the preoperative diagnosis and staging of peritoneal carcinomatosis: a prospective multireader pilot study

N. Vietti Violi[1], S. Gavane[2], P. Argiriadi[2], A. Law[2], S. Heiba[2], M. Ghesani[2], E. Bekhor[3], J. Babb[2], D. Labow[2], B. Taouli[2]; *[1]Lausanne/CH, [2]New York, NY/US, [3]Petah Tikva/IL*

**Purpose:** To assess the diagnostic performance of FDG-PET/MRI for the preoperative diagnosis and staging of peritoneal carcinomatosis (PC) using surgical Sugarbaker's PC index as the reference in a multireader pilot study.

**Material and Methods:** Fourteen adult patients (M/F: 3/11, mean age: 57±12y) with PC were prospectively included in this single-center study. Patients underwent FDG-PET/MRI prior to surgery (mean delay: 14d, range: 1–63d). Images were reviewed independently by 2 abdominal radiologists and 2 nuclear medicine physicians. The abdomen was divided into 13 regions, scored from 0 to 3. The radiologists assessed contrast-enhanced abdominal MR images, while the nuclear medicine physicians assessed PET images fused with T2w images. A hybrid FDG-PET/MRI radiological PCI was created by combining the study data. Inter-reader agreement was evaluated. Radiological PCI was compared to the surgical PCI on a per-patient and per-region basis.

**Results:** Mean surgical PCI was 10±8 (range: 0–24). Interreader agreement was almost perfect for all sets for radiologic PCI (Kappa: 0.81–0.98). PCI scores for all reading sets significantly correlated with the surgical PCI score (r range: 0.57–0.74, p range: <0.001–0.003). Pooled per-patient sensitivity, specificity and accuracy were 75%/50%/71.4% for MRI, 66.7%/50%/64.3% for FDG-PET, and 91.7%/50%/85.7% for FDG-PET/MRI, without significant difference. FDG-PET/MRI achieved 100% sensitivity and specificity for a cutoff PCI of 20. Per-region sensitivity, specificity and accuracy were 37%/78.4%/61.8% for MRI, 17.8%/95%/64.3% for FDG-PET and 52.7%/66.5%/60.4% for FDG-PET/MRI, with significant difference between sets for sensitivity and specificity.

**Conclusion:** FDG-PET/MRI achieved an excellent diagnostic accuracy per-patient and weaker performance per-region for detection of PC.

### SS 12.3

### Dual-energy CT for the identification and quantification of peritoneal metastases: stepping forward CT contrast resolution

G. Bagnacci, S. Guerrini, F. Gentili, L. Volterrani, M. Mazzei, L. Sansotta, I. Monteleone; *Siena/IT*

**Purpose:** To investigate the benefits of dual-energy CT (DECT) in patients with peritoneal carcinomatosis (PC).

**Material and Methods:**Patients' selection: ten patients with and ten without PC were selected. Inclusion criteria comprehended: Bioptic confirmation and evaluation of peritoneal cancer index (PCI) with laparotomy within 30 days from CT. No chemotherapy treatment. CT comprises at least a portal (70s) and an equilibrium (180–300s) phase (the latter performed with dual energy). Readers' selection: readers were selected based on experience (3 levels, 3 readers/level). Workflow: CT examinations from the 20 patients were anonymized in two different series, one comprising portal and equilibrium phase and one with the addition of low virtual monoenergetic and iodine maps. Readers filled in a form about the presence of PC and evaluation of PCI in the 40 examinations.

**Results:** Accuracy in the identification of PC for single patients passed from 73% to 81% with the second set. Inexperienced readers progressed from 60 to 75% while experts from 88 to 90%. Regarding the identification of PC for a single region, accuracy improved from 76 to 80% with profit even for the expert reader (accuracy 83 to 88%, diagnostic odds ratio from 39 to 132). Interreader agreement resulted to be improved with DECT. The mean difference between laparotomic and CT PCI was 14 with the first set of images and 10 for the second; for expert readers from 12 to 8.

**Conclusion:** DECT at the equilibrium phase showed a benefit for all the readers in the identification and quantification of peritoneal carcinomatosis.

### SS 12.4

### Deep-learning image reconstruction for low-dose liver CT

A. Del Gaudio, G. Guido, D. Caruso, N. Ubaldi, D. Valanzuolo, G. Bona, D. Pugliese, A. Laghi; *Rome/IT*

**Purpose:** To evaluate image quality, image noise and potential dose reduction of low-dose CT scans of the abdomen with deep-learning image reconstruction (DLIR) and to compare with images reconstructed with the adaptive statistical iterative reconstruction at a level of 50% (ASIR-V50%) and 100% (ASIR-V100%).

**Material and Methods:** Consecutive patients who underwent abdominal enhanced CT were prospectively enrolled from 1st August to 30th September, 2021. Exclusion criteria were: contraindication to CT and severe motion artifacts on CT. Datasets were acquired with ASiR-V50% and then reconstructed with ASiR-V100% and DLIR at high levels (DLIR-H), and data regarding radiation dose were collected. Two radiologists in consensus performed the objective image quality analysis of images using standardized regions of interest (ROIs) to record mean attenuation value and standard deviation (SD) in Hounsfield units (HU) for the liver, aorta, portal vein and muscle. Contrast-to-noise (CNR) and signal-to-noise (SNR) were assessed. Two radiologists independently evaluated subjective image contrast, image noise and conspicuity of structures using a 5-point Likert scale.

**Results:** Sixty patients were enrolled (39 male, mean age 67±13y). SNR of liver parenchyma in arterial phase was significantly higher for DLIR compared to ASIR-V100% (p=0.04), whereas no significant differences were observed for SNR of the liver in the portal phase (p=0.09). No significant differences were observed in terms of CNR of the liver parenchyma in arterial phase between DLIR and ASIR-V100% (p=0.52), whereas DLIR CNR was significantly higher in portal phase (p=0.04). According to the subjective analysis, DLIR had higher image contrast, lower image noise and better conspicuity of structures than ASIR-V100% (all p<0.001), with excellent inter-rater agreement (k=0.81). With respect to ASIR-V100%, radiation dose was significantly lower in DLIR (p=0.031).

**Conclusion:** DLIR significantly improved the image quality and reduced image noise compared to ASIR-V100% while reducing the radiation dose.

### SS 12.5

### Are dilution and slow injection with fluoroscopic triggering technique the solution to mitigating arterial-phase artifacts in gadoxetic acid-enhanced liver MRI?

R. Ambros, S. Poetter-Lang, A. Messner, A. Kristic, J.C. Hodge, N. Bastati-Huber, A. Ba-Ssalamah; *Vienna/AT*

**Purpose:** To evaluate the effect of dilution and slow injection of gadoxetic acid (GA) using automated fluoroscopic triggering on the frequency of arterial-phase artifacts.

**Material and Methods:** Three independent readers evaluated 1985 liver-MRIs routinely done with a fixed bolus of 10 ml GA diluted with 10 ml saline, injected at 1 mL/s using automated fluoroscopic triggering on a 3T MR machine. All readers graded severity of artifacts and their impact on diagnostic performance on a 5-point-scale system. Two readers assessed the type of artifacts: Gibbs, TSM or both. One reader evaluated arterial-phase acquisition-timing and the presence of ascites, pleural effusions and cirrhosis.

**Results:** A total of 1793 exams (male = 852 (49.7%), female = 863 (50.3%)), with a mean age of 56.3 y, were included. In 366 (20.4%) exams, there was ascites, in 319 (17.8%), pleural effusions and liver cirrhosis in 352 (19.6%). Arterial-phase images of diagnostic quality included 1163 (64.9%) images without artifacts, 415 (23.1%) with minimal, and 171 (9.5%) with moderate artifacts. In only 44 patients (2.5%), 39 (2.2%) with severe arterial-phase artifacts and 5 (0.3%) with uninterpretable images were the exams non-diagnostic.The inter-rater agreement (Kappa = 0.670, p<0.001) was substantial. Acquisition-timing for AP imaging was optimal in 1567 (87.4%) exams. The number of artifacts found was significantly higher in the presence of ascites (p=0.002) and pleural effusions (p=<0.001), as well as in advanced age (p=0.006).

**Conclusion:** The combination of a diluted and slowly injected bolus of gadoxetic acid using MRI fluoroscopic triggering provides properly timed arterial phase imaging and reduced severe- or non-diagnostic artifacts in the arterial phase to only 2.5%.

### SS 12.6

### Reduction of contrast agent volume and radiation dose in pancreatic dynamic CT by a combination of 90KVP tube voltage, high tube current modulation, and advanced iterative reconstruction algorithm

M.S. Park, H.I. Ha, J.-H. Ahn; *Anyang-si/KR*

**Purpose:** To evaluate the image quality and the reduction of radiation dose in CT performed with a 90-kVp tube voltage, high tube current modulation, and 70% of the standard amount of contrast medium (case group) compared to CT performed using a 100-kVp tube voltage and the standard amount of contrast medium (control group).

**Material and Methods:** 51 patients (65.9 ± 9.4 years) who underwent both CT protocols were included in this retrospective study. One reviewer measured the attenuation values of the abdominal organs and image noise for objective image quality analysis. Two radiologists evaluated the five categories of images for subjective image quality analysis. All statistical analyses were performed using pair-wise comparisons.

**Results:** Compared with the control group, in the case group, the total amount of the contrast agent, the radiation dose, and image noise decreased by 24.4%, 31.7%, and 20.6%, respectively ($p < 0.001$). The intraobserver and interobserver agreements were moderate to substantial. The SNR and CNR of the aorta, liver, main portal vein, pancreas, spleen, and kidney in the case group were significantly higher ($p < 0.001$), whereas the SNR of the psoas muscle was not significantly different ($p = 0.135$). Except for lesion conspicuity, both reviewers judged that the subjective image quality of the case group was better than that of the control group ($p < 0.001$).

**Conclusion:** The combination of the 90-kVp tube voltage and 70% of the standard contrast agent dose with iterative reconstruction and high tube current achieved radiation dose reduction of 31.7%, as well as better image quality and diagnostic confidence.

### SS 12.7

### Comparison of CAIPIRINHA-VIBE and conventional-VIBE sequences in liver MRI

M. Orvieto, D. Urso, F. Crimì, E. Quaia, F. Vernuccio; *Padua/IT*

**Purpose:** The aim of this study was to assess whether a short breath-hold technique T1 VIBE sequence acquired using the CAIPIRINHA sequence can improve image quality compared with a conventional long breath-hold VIBE.
**Material and Methods:**We retrospectively included consecutive patients undergoing liver MRI, with conventional T1W-VIBE on precontrast phase and CAIPIRINHA-VIBE both on precontrast and arterial phases. An expert abdominal radiologist assessed image quality (CNR, breath hold, spatial resolution, overall imaging quality, adequacy of hepatic arterial phase). If prior liver MRI was available, analysis of conventional T1W-GRE on arterial phase was also performed. Image quality was then compared between conventional T1W-VIBE and CAIPIRINHA-VIBE on precontrast and arterial phase.
**Results:** Our study included 28 patients (16M, 12W, mean age, 59 years). Of these, 6 had prior liver MRI performed with conventional T1W-VIBE. On precontrast, both the conventional T1W-VIBE and the CAIPIRINHA-VIBE sequences showed good to excellent CNR in 26/28 (89.3%) patients and 24/28 (85.7%) and overall quality in 25/28 (89.3%) and 26/28 (92.9%), respectively; breath-hold was always adequate with CAIPIRINHA-VIBE while it was inadequate in 1/28 (0.04%) patients with conventional T1W-VIBE. On arterial phases, timing of acquisition was adequate in all patients with CAIPIRINHA-VIBE, while it was inadequate in 2/6 (33.3%) patients imaged with conventional T1W-VIBE; CNR, spatial resolution and overall quality of images were at least sufficient in all patients.
**Conclusion:** CAIPIRINHA-VIBE seems to reduce breath-hold artifacts and improve adequacy of hepatic arterial phase compared to conventional T1W-VIBE, while maintaining similar image quality.

### SS 12.8

### Contribution of cine MRI sequences in local staging of esophageal cancer

L. Haefliger, M. Jreige, N. Villard, C. Du Pasquier,
J.-B. Ledoux, N. Vietti Violi, C. Dromain; *Lausanne/CH*

**Purpose:** To assess the diagnostic potential of cine MRI sequences for T staging of esophageal cancer (EC).
**Material and Methods:** This prospective monocentric study included 54 patients with histologically proven EC. Each patient underwent MRI for initial staging. MR was acquired on 3T scanner using morphological (T2-TSE Blade, gated T2-TSE Dark-Blood, T1-VIBE Dixon with and without contrast IV), functional (DWI and ADCmap) and cine sequences (steady-state-free precession and real-time TrueFISP during water ingestion). Three readers reviewed static and cine-MR images to assess T-staging. Reference standard was histologic or T-staging resulting from the analysis of all endoscopic and imaging procedures.
**Results:** Tumors were classified T1/T2 (n=7, 13%), T3 (n=29, 53.7%), T4a (n=12, 22.2%) and T4b (n=6, 11.1%) on static sequence (S-MRI) and T1/T2 (n=7, 13.2%), T3 (n=27, 51%), T4a (n=13, 24.5%) and T4b (n=6, 11.3%) on static+cine sequences (SC-MRI). SC-MRI T-staging showed a significant correlation with reference Tstaging (rs = 0.667, P < 0.001). SC-MRI showed similar performance than S-MRI in distinguishing T1/T2 from T3/T4 with a sensitivity, specificity and AUC of 95.8%, 75% and 0.864, but a slightly better performance in distinguishing T1–T3 from T4 with a sensitivity, specificity and AUC of 76.5%, 83.8% and 0.801 and 70.6%, 83% and 0.772, respectively. Intrareader agreement for T3 and T4 stages was higher for SC-MRI than S-MRI (k=0.403 and 0.498 vs 0.376 and 0.1222). SC-MRI showed increased T-staging confidence over S-MRI for all readers (17%–24%).
**Conclusion:** Adding cine sequences to static MRI increased sensitivity and improved interreader agreement on T staging, notably for higher stages.

### SS 12.9

### MR angiography with inflow-sensitive inversion recovery technique for upper abdominal arteries assessment: comparison with contrast-enhanced MRI and CT angiography

C. Cazzella[1], P. Bonaffini[1], R. Simonini[1], M. Porta[2],
C. Maino[3], L. Dulcetta[1], F. Carbone[1], P. Marra[1], P. Brambilla[1],
S. Sironi[3]; [1]*Bergamo/IT,* [2]*Monza/IT,* [3]*Milan/IT*

**Purpose:** To assess unenhanced inflow-sensitive inversion recovery (IFIR) sequence accuracy in the evaluation of upper abdominal arteries, compared to contrast-enhanced MRI (CE-MRI) and CT angiography (CTA).
**Material and Methods:** Patients undergoing upper abdomen MRI for several clinical purposes were prospectively enrolled. The MRI protocol included contrast-enhanced study and IFIR-MRA sequence. MR images were compared with CTA (whenever available), used as reference standard. All images were independently analysed by two readers using a qualitative 4-point scale (1 = poor image quality, 4 = excellent image quality). Celiac trunk (CA), common–proper–left–right hepatic artery (C–P–L–R HA), left gastric artery (LGA), gastroduodenal artery (GA), splenic artery (SA), renal arteries (RA) and superior mesenteric artery (SMA) were assessed.
**Results:** Seventy patients (M/F = 45/25; mean age 58 years, range 12–86) were included. CE-MRI was performed in 65 cases and CTA was available in 42. IFIR-MRA images were better rated compared to CE-MRI: particularly, all arteries obtained a statistically significant higher qualitative rating value (all $p<0.05$). IFIR-MRA and CE-MRI exhibited acceptable intraclass correlation coefficients (ICC) values for CA, C–L–R HA, and SMA (ICC 0.507, 0.591, 0.615, 0.570, 0.525). IFIR-MRA and CTA showed significant correlations in C–P–L–R HA ($\tau=0.362$, 0.261, 0.308, 0.307, respectively; $p<0.05$), and in RA ($\tau=0.279$, $p<0.05$).
**Conclusion:** IFIR-MRA is superior to CE-MRI in terms of visualization of upper abdomen arteries, especially the main left and right hepatic branches. IFIR-MRA and CTA reported significant correlation, contrary to CE-MRI and CTA. Accordingly, IFIR could be routinely included in upper abdomen MR studies, especially in cases where contrast is contraindicated, or life-long follow-up is expected.

ON DEMAND

### *Scientific Session On Demand SSD 1*
### Miscellaneous

### *SSD 1.1*
### Safety of percutaneous, image-guided biopsy of hepatocellular carcinoma with and without concurrent ablation
J. Tse[1], K. Terashima[2], L. Shen[1], D. Lu[3], S. Raman[3];
[1]*Stanford, CA/US,* [2]*San Francisco, CA/US,*
[3]*Los Angeles, CA/US*

**Purpose:** To determine the prevalence of adverse events after image-guided biopsy of histologically proven hepatocellular carcinomas (HCC) using a standardized, indirect access, coaxial biopsy technique in a contemporary series.
**Material and Methods:** In this institutional review board-approved, Health Insurance Portability and Accountability Act-compliant, and retrospective study, we evaluated all consecutive adult patients from 2011 to 2016 who underwent image-guided biopsy of HCC with and without concurrent ablation. Tumor seeding was defined as any new lesion along the needle tract on subsequent imaging. Adverse events were graded using both the Clavien–Dindo Complication Classification system and the most recently proposed Society of Interventional Radiology (SIR) Adverse Event Classification System.
**Results:** A total of 383 patients underwent 398 biopsies (64±11 years; 112 women, 271 men). Most patients (282; 71%) underwent concurrent ablation. Adverse events occurred after 18 biopsies (4.5%): 13 were Grade I (Clavien–Dindo) or minor (SIR) and included hematoma (7), hepatic vein thrombus (2), portal vein thrombus (2), moderate pleural effusion (1), and small pneumothorax (1). The remaining 5 (1.3%) adverse events were classified as Grade II–IIIa (Clavien–Dindo) or moderate (SIR) and included hematoma requiring blood products (n=1), arrhythmia (n=1), and symptomatic pleural effusions requiring treatment (n=3). Baseline age, etiology of liver disease, HCC diameter, and HCC grade were not associated with adverse events. There were no tumor seeding events after a median follow-up of 611 days (interquartile range 211–1104).
**Conclusion:** Percutaneous image-guided tissue sampling using a standardized, indirect access, coaxial technique can be performed safely without observed tumor seeding in a contemporary series.

### *SSD 1.2*
### Growth kinetics of pancreatic neuroendocrine tumors by histopathologic grade
J. Tse, L. Shen, A. Kamaya; *Stanford, CA/US*

**Purpose:** To determine if growth kinetics of pancreatic neuroendocrine tumors (PNETs) are associated with histologic grade.
**Material and Methods:** 50 treatment-naïve PNETs from 50 adult patients (57±14 years; 29 women, 21 men) from 2010 to 2021 with serial CT/MRI separated by at least 3 months and histopathology were included for analysis. Orthogonal dimensions of PNETs were measured for each PNET. Growth kinetics were assessed with volume doubling time (VDT) using the modified Schwartz equation. Qualitative imaging features were evaluated by two abdominal radiologists blinded to histopathology. Histopathologic grade was assigned using the 2017 WHO classification system.
**Results:** Of 50 PNETs, 38 (76%) were grade 1 and 12 (24%) were grade 2–3. Median VDT for grade 1 PNETs was 1.8 years (interquartile range IQR 1.4–6.4 years) while median VDT for grade 2–3 PNETs was 9.4 years (IQR 3.7–70 years; p=0.003). Grade 2–3 PNETs were more likely to have local mass effect (defined as vascular involvement, main duct dilation, or distal pancreatic atrophy; 58% vs 21%; p=0.027), radiologic evidence of metastases at baseline imaging (50% vs 13%; p=0.014), and VDT <3 years (75% vs 18%) compared to grade 1 PNETs. Baseline size, presence of calcifications, and tumor vascularity relative to the rest of the pancreas were not associated with grade. At multivariable analysis, VDT was the most significant predictor of PNET grade (odds ratio OR 3.9) followed by radiologic evidence of metastases (OR 2.1) and local mass effect (OR 1.6).
**Conclusion:** High growth kinetics defined by volume doubling time are associated with higher grade pancreatic neuroendocrine tumors.

### *SSD 1.3*
### Colonoscopy versus catheter angiography for lower gastrointestinal bleeding following localization on CT angiography
J. Tse[1], E. Felker[2], G. Tse[2], T. Liang[1], J. Shen[1], A. Kamaya[1];
[1]*Stanford, CA/US,* [2]*Los Angeles, CA/US*

**Purpose:** To compare catheter angiography (CA) and colonoscopy outcomes following successful CT angiographic (CTA) localization for patients with overt lower gastrointestinal bleeding (LGIB).
**Material and Methods:** In this retrospective study, 71 consecutive adult patients (24 women, 47 men; 69±17 years) from two institutions between 2010 and 2020 had both contrast extravasation on CTA in the lower gastrointestinal tract and subsequent CA or colonoscopy. The primary outcome was confirmation of active bleeding during CA or colonoscopy (defined as confirmation yield). The secondary outcomes were to determine therapeutic yield (hemostatic therapy), time-to-procedure, rebleeding rate, and adverse outcome rates (defined as surgery, acute kidney injury, initiation of dialysis, overall mortality). Univariate analyses and multivariable analyses with p<0.05 were used to determine statistical significance.
**Results:** 44 patients underwent CA and 27 underwent colonoscopy. CA had higher overall confirmation yield (55 vs 26%; p=0.026) while therapeutic yields were similar (70 vs 56%; p=0.214). Time-to-procedure was 5.1±3.4 hours for CA and 15.5±13.6 hours for colonoscopy (p<0.0001). At multivariable analyses, shorter time-to-procedure was the only statistically significant predictor for confirmation yield (p=0.037) and therapeutic yield (p=0.013), while procedure type, nadir hemoglobin, number of transfusions, and hemodynamic instability were not. Adverse events and rebleeding were not statistically different between patients who underwent CA and colonoscopy (p>0.05).
**Conclusion:** Shorter time-to-procedure was the only statistically significant predictor for confirmation and therapeutic yield after CTA localization of LGIB. Because CA can be performed sooner than colonoscopy without increased rates of adverse outcomes or rebleeding, CA may be a reasonable first-line treatment option in patients after CTA localization of LGIB.

### *SSD 1.4*
### Application of apparent diffusion coefficients derived from intratumoral and peritumoral zone for assessing pathologic prognostic factors in rectal cancer
H. Li, Y. Yuan, H. Pu; *Chengdu/CN*

**Purpose:** To investigate the diagnostic performance of the apparent diffusion coefficient (ADC) derived from intratumoral and peritumoral zone for the assessment of pathologic prognostic factors in resectable rectal cancer.
**Material and Methods:** A total of 146 patients with rectal cancer, who underwent radical surgery within 1 week of MRI, were prospectively enrolled. Two radiologists independently placed free-hand regions of interest (ROIs) in the largest tumor cross section and three small ROIs on the peritumoral zone adjacent to the tumor contour. Maximum values of tumor ADC (ADCtmax), minimum values of tumor ADC (ADCtmin), mean values of tumor ADC (ADCtmean), mean values of peritumor ADC (ADCpmean), and ADCpmean/ADCtmean (ADC ratio) were obtained on ADC maps and correlated with prognostic factors using univariate, multivariate, and receiver operating characteristic (ROC) curve analyses.
**Results:** Interobserver agreement was excellent for ADCtmax, ADCtmin, ADCtmean and ADC ratio (intraclass correlation coefficient [ICC], 0.815–0.928) and moderate for ADCpmean (ICC, 0.562). Both ADC ratio and ADCpmean had higher areas under the ROC curve (AUC) of 0.700 and 0. 664, 0.707 and 0.663, 0.776 and 0.748, 0.677 and 0.686, 0.848 and 0.819, and 0.778 and 0.735 for discriminating well-moderate from poor differentiation, T1–2 from T3–4 classification, lymph node metastasis (LNM) negative from LNM positive, extranodal extension (ENE) negative from ENE positive, tumor deposit (TD) negative from TD positive, lymphovascular invasion (LVI) negative from LVI positive, respectively.
**Conclusion:** Both ADC ratio and ADCpmean could be used to assess prognostic factors. Particularly, ADC ratio showed most capability for the assessment of prognostic factors in resectable rectal cancer.

European Society
ESGAR
Gastrointestinal and Abdominal Radiology

### A

Abbas N.: SS 8.4
Abkhoo A.: SS 9.1
Abu Hilal M.: SS 11.8
Abyzov A.: SS 10.5
Acquafredda F.: SS 5.8
Agarwal A.: SS 7.9
Agarwal A.: SS 7.9
Agarwal G.R.: **SS 8.6**
Ahn J.-H.: SS 12.6
Akinosoglou K.: SS 6.9
Akselrod D.G.: SS 11.5
Alan B.: **SS 9.2**, **SS 9.3**
Alan S.: SS 9.2
Albazaz R.: SS 10.3
Alderson S.: SS 3.7
Ali S.: SS 7.6
Aluffi G.: SS 2.8
Amaddeo G.: SS 4.3
Ambros R.: SS 2.3, SS 8.2, **SS 12.5**
Ansett J.: SS 1.6
Argiriadi P.: SS 12.2
Arndtz K.: SS 8.4
Assouline J.: **SS 8.7**
Aubé C.: SS 4.1, SS 4.4, SS 4.6
Auernhammer C.: SS 8.10
Autelitano D.: SS 2.7, SS 2.8, SS 2.9, **SS 5.2**, SS 10.9
Ayoobi Yazdi N.: SS 9.1

### B

Ba-Ssalamah A.: SS 2.3, SS 8.2, SS 10.6, SS 12.5
Babb J.: SS 12.2
Bacquet R.: SS 5.4
Bae J.: SS 8.5
Bagnacci G.: **SS 12.3**
Bakers F.: SS 3.2
Balcar L.: SS 10.1
Banerjee M.: SS 7.9
Banerjee R.: SS 5.3, SS 7.6, SS 8.3
Bastati-Huber N.: SS 2.3, SS 8.2, **SS 10.6**, SS 12.5
Bates D.D.: SS 1.5
Baubeta-Fridh E.: SS 8.3
Beek K.J.: **SS 6.4**, SS 6.6, **SS 6.8**
Beer A.: SS 6.5
Beer L.: SS 10.1, SS 10.6
Beets G.L.: SS 3.2, SS 3.10, SS 11.4
Beets-Tan R.G.H.: SS 3.2, SS 3.10, SS 11.1, SS 11.2, SS 11.3, SS 11.9, SS 11.10
Bekhor E.: SS 12.2
Ben Abdallah I.: SS 9.4, SS 9.6
Bengtsson J.: SS 8.3
Benson S.: SS 11.10
Berger F.: SS 8.10
Bergmann M.: SS 6.5
Bergquist A.: SS 8.3
Berzigotti A.: SS 10.7
Besa C.: SS 4.2
Besselink M.: SS 11.8
Bhagwanani A.: SS 12.1
Bhatnagar G.: SS 6.1
Bijot J.-C.: SS 5.4, SS 9.6
Bilreiro C.: **SS 2.2**
Bipat S.: SS 11.8
Bjartmar C.: SS 11.7
Boers T.: SS 2.5
Bogner P.: SS 7.2
Bogveradze N.: **SS 3.2**, SS 11.1, SS 11.2, SS 11.3
Bona G.: SS 12.4
Bonaffini P.A.: SS 5.6, SS 5.7, SS 9.5, SS 12.9
Bonanomi A.: SS 9.5
Bouktif I.: SS 7.1
Boutron I.: SS 5.10
Brady M.: SS 5.3
Brambilla P.: SS 12.9
Bricault I.: SS 4.6
Brismar T.: SS 11.7
Broekmans J.: SS 2.5
Brouwer M.A.: SS 2.4
Brown P.J.: SS 3.1, SS 3.7, SS 3.8
Buffart T.: SS 11.9
Burr N.E.: SS 1.2
Busard M.: SS 11.10
Buskens C.: SS 6.8

### C

Caballol B.: SS 6.2
Calderaro J.: SS 4.3
Canivet C.M.: SS 4.4
Cannella R.: **SS 4.5**, SS 8.7, SS 10.2
Capanu M.: SS 1.5
Capozzi N.: SS 6.2
Carbone F.: SS 5.6, SS 5.7, SS 12.9
Cardobi N.: SS 10.9
Carli A.: SS 2.7, SS 2.9, SS 5.2, SS 10.9
Caruso D.: SS 1.7, SS 10.10, SS 12.4
Carvalho T.: SS 2.2
Casanova N.: SS 10.3
Cassinotto C.: SS 4.4
Castier Y.: SS 9.4, SS 9.6
Castillo-Martin M.: SS 2.2
Catucci D.: SS 10.7
Cauchy F.: SS 10.2
Cazzella C.: **SS 12.9**
Cercek A.: SS 1.5
Chan W.Y.: SS 5.3
Chandramohan A.: **SS 3.3**
Chaniotaki N.: **SS 6.9**
Chavarrias C.: SS 2.2
Chong Toh H.: SS 5.3
Choo S.P.: SS 5.3
Choon T.H.: SS 5.3
Chouard A.: SS 4.1, SS 5.4
Choudhary G.: SS 7.9
Chow P.: SS 5.3
Christe A.: SS 10.7
Chua C.: SS 5.3
Chua H.S.: SS 5.3
Cicalo' F.: SS 2.8, SS 10.9
Coates J.D.: SS 3.8
Cockmartin L.: SS 2.10
Collegaro D.: SS 9.10
Connell J.: SS 5.3
Conticchio M.: SS 5.8
Contro A.: SS 5.9
Corallo C.: **SS 10.3**
Corcos O.: SS 9.4, SS 9.6
Corso R.: SS 1.3, SS 7.3, SS 10.4
Cortese F.: SS 5.8
Couvelard A.: SS 7.4
Crimì F.: SS 12.7
Curvo-Semedo L.: SS 11.1, SS 11.2, SS 11.3
Custers P.: **SS 3.10**, **SS 11.4**
Cuthbertson D.J.: SS 7.6
Cyr D.: SS 9.10

### D

D'Haens G.R.: SS 6.8
D'Onofrio M.: SS 2.7, SS 2.8, SS 2.9, SS 5.2, SS 10.9
Dam C.: SS 1.8
Danti G.: SS 2.1, SS 9.9
de Jong J.E.: SS 7.7
de Jonge C.S.: SS 6.6
de Louis L.: SS 8.7
de Mestier L.: SS 7.1
De Robertis R.: SS 2.7, **SS 2.9**, SS 5.2, SS 10.9
De Robertis Lombardi R.: SS 5.9
de With P.: SS 2.5
Deijen C.L.: SS 11.6
Del Gaudio A.: **SS 12.4**
Delagnes A.: **SS 4.6**
Di Fazio B.: **SS 5.7**
Dickson B.: SS 9.10

Dioguardi Burgio M.: SS 4.5, **SS 5.4**, SS 7.4, SS 8.7, SS 9.6, SS 10.2, SS 10.5, **SS 10.8**
Dionysopoulos K.: SS 6.9
Dogan H.: SS 7.10
Dondero F.: SS 10.8
Dresen R.: SS 2.10
Dromain C.: SS 12.8
Du Pasquier C.: SS 12.8
Dulcetta L.: **SS 5.6**, SS 5.7, SS 12.9
Duran R.: SS 8.7
Durand F.: SS 10.8
Duxbury O.: SS 12.1

### E

Eapen A.: SS 3.3
Ebner L.: SS 10.7
Eddowe P.: SS 8.4
Eichert N.: SS 7.6
El Khababi N.: SS 3.2, **SS 11.1**, **SS 11.2**, **SS 11.3**
El-Sheikha J.: **SS 12.1**
Esengur O.T.: SS 7.10

### F

Faluhelyi N.: **SS 7.2**
Fedeli F.: **SS 2.1**, SS 9.9
Felker E.: SSD 1.3
Fernandes F.F.: SS 2.2
Fernandez-Clotet A.: SS 6.2
Ferreira C.: **SS 8.4**
Fichera A.: SS 5.3
Firat C.: SS 1.5
Fogel E.: SS 7.5
Forsman C.: SS 8.3
Franco P.N.: **SS 9.5**
Fung A.: SS 11.5

### G

Galli A.: SS 2.1
Gandhi A.: SS 8.6
Ganesan K.: SS 4.10
Gangai N.: SS 11.5
Garcia-Aguilar J.: SS 1.5, SS 11.5
Garg M.K.: SS 7.9
Garg P.: SS 7.9
Garrett J.: SS 1.4
Garteiser P.: SS 10.5
Garzelli L.: SS 5.4, **SS 9.4**, **SS 9.6**
Gasparini C.: SS 5.9
Gasperment M.: SS 7.1
Gavane S.: SS 12.2
Gecse K.: SS 6.8
Gentili F.: SS 12.3
Geraci L.: **SS 2.7**, SS 2.8, SS 2.9, SS 5.2, SS 10.9
Ghesani M.: SS 12.2
Giandola T.: SS 1.3, SS 7.3, SS 10.4
Gibbons K.: SS 7.6
Gladdy R.: SS 9.10
Gogna A.: SS 5.3
Goh B.: SS 5.3
Gollub M.J.: SS 1.5, SS 11.5
Goody R.: SS 10.3
Görgec B.: **SS 11.8**
Goyal H.: SS 7.9
Grazzini G.: SS 9.9
Greco G.: SS 9.10
Gregory J.: SS 5.4, **SS 5.10**, SS 9.6
Grigoriadis A.: **SS 8.3**
Gronchi A.: SS 9.10
Grotenhuis B.A.: SS 11.6
Gruzdev I.: **SS 8.9**
Guenthner C.: SS 7.8
Guerendel C.: SS 11.10
Guerrini S.: SS 12.3
Guido G.: SS 12.4
Guilloux A.: SS 7.1
Guiu B.: SS 4.6
Guneyli S.: **SS 7.10**
Guo J.: SS 7.7
Gupta G.: SS 8.6
Gupta Y.: SS 8.6
Gurel S.: SS 9.2, SS 9.3

### H

Ha H.I.: SS 12.6
Hacker M.: SS 6.5
Haefliger L.: **SS 12.8**
Halligan S.: SS 6.1
Hamid A.: SS 7.6
Hamon A.: SS 9.4
Han S.: SS 4.9, SS 9.8
Handley K.: SS 1.9
Hansen T.F.: SS 1.8
Harmath C.B.: SS 11.5
Hassoy H.: SS 7.10
Haug A.: SS 6.5
Heeres B.: SS 11.9
Heiba S.: SS 12.2
Hentic O.: SS 8.7
Hermans J.J.: SS 2.4, **SS 2.5**
Hirschfield G.M.: SS 8.4
Hodge J.C.: SS 12.5
Hogenes A.M.: SS 2.4
Horsthuis K.: SS 6.8
Huber A.: SS 10.7
Huibregtse I.: SS 3.10
Huisman H.: SS 2.5
Hulkower M.: SS 4.2

### I

Iannicelli E.: SS 1.7, SS 10.10
Ianus A.: SS 2.2
Inchingolo R.: **SS 5.8**
Ingenerf M.K.: **SS 8.10**
Innocenti T.: SS 2.1
Išgum I.: SS 6.6
Ismail M.: SS 1.1

### J

Jagirdar N.: SS 3.9
Jais B.: SS 7.1
Jalkote S.: SS 4.10
Janse M.: SS 2.5
Jemal Turki A.: SS 7.1
Jeon S.K.: SS 9.8
Jhaveri K.S.: SS 8.3
Jo S.J.: SS 3.5
Jreige M.: SS 12.8

### K

Kalogeropoulou C.: SS 6.9
Kamaya A.: SSD 1.2, SSD 1.3, SS 4.7, SS 4.8
Kampalath R.: SS 11.5
Kang H.-J.: **SS 4.9**, SS 8.5
Karabiber Deveci C.: **SS 2.6**
Karmazanovsky G.: SS 8.9
Kartalis N.: SS 8.3, SS 11.7
Kelly M.: SS 5.3
Kemp G.: SS 7.6
Khalili K.: **SS 9.10**
Kharrat R.: SS 4.3
Khera P.: SS 7.9
Kiesl S.: SS 8.10
Kim J.: SS 4.9
Kim J.H.: **SS 8.5**
Kim J.H.: SS 8.5
Kim M.G.: SS 9.8
Kim S.H.: **SS 9.8**
Kim S.H.: **SS 3.5**
Kirchner A.: SS 10.8
Klompenhouwer E.: SS 11.9, SS 11.10
Kondratyev E.: SS 8.9
Koo S.L.: SS 5.3
Korsavidou-Hult N.: SS 8.3
Kothari N.: SS 7.9
Kranz C.: SS 7.5
Krehbiel K.: SS 11.5
Kristic A.: **SS 2.3**, SS 8.2, SS 12.5

Kuhlmann K.: SS 11.9
Kumar S.: **SS 6.1**
Kusters M.: SS 3.2

### L

Labow D.: SS 12.2
Laghi A.: SS 1.7, SS 10.10, SS 12.4
Lahaye M.J.: SS 3.2, SS 11.1, SS 11.2, SS 11.3
Lambregts D.: SS 3.1, SS 3.2, SS 3.10, SS 11.1, SS 11.2, SS 11.3, SS 11.4, **SS 11.6**, SS 11.9
Lampichler K.: SS 10.1
Latiff J.A.: SS 5.3
Laurent A.: SS 4.3, SS 10.2
Laurent V.: SS 4.6
Law A.: SS 12.2
Lebtahi R.: SS 10.8
Leclercq I.: SS 10.5
Ledoux J.-B.: SS 12.8
Lee J.: SS 4.9
Lee M.H.: SS 1.4
Lee S.: SS 11.5
Lesurtel M.: SS 10.8
Levy P.: SS 7.1
Lewis S.: SS 4.2
Li H.: **SSD 1.4**
Liang T.: SSD 1.3, SS 4.7
Lim K.H.: SS 5.3
Lindebjerg J.: SS 1.8
Litjens G.: SS 2.5
Littlejohns A.: SS 10.3
Liu P.S.: SS 11.5
Lo R.H.: SS 5.3
Lokhandwala D.: SS 4.10
Longo C.: SS 2.7, **SS 2.8**, SS 2.9, SS 5.2, SS 10.9
Lorenzo D.: SS 7.1
Lu D.: SSD 1.1
Lubner M.G.: SS 1.4
Luciani A.: SS 4.3, SS 10.2

### M

Maas M.: SS 3.2, SS 3.10, SS 11.1, SS 11.2, SS 11.3, SS 11.4, SS 11.9, SS 11.10
Magill L.: SS 1.9
Maino C.: **SS 1.3**, **SS 7.3**, **SS 10.4**, SS 12.9
Maire F.: SS 7.1
Mallett S.: SS 6.1
Mambrin F.: **SS 5.9**
Mandorfer M.: SS 10.6
Mang T.: SS 6.5
Mansueto G.: SS 5.9
Marangos M.: SS 6.9
Mardighian A.: SS 5.8
Marijnen C.: SS 3.10
Marra P.: SS 5.6, SS 5.7, SS 9.5, SS 12.9
Masci B.: SS 10.10
Matos C.: SS 2.2
Matteini F.: **SS 10.2**
Mazzei M.: SS 12.3
Mehta D.: **SS 4.10**
Meij M.: SS 2.4
Meischl T.: SS 10.1
Melling P.: SS 1.2
Memeo R.: SS 5.8
Messner A.: SS 2.3, SS 8.2, SS 12.5
METRIC study Investigators: SS 6.1
Miele V.: SS 2.1, SS 9.9
Millet J.D.: SS 11.5
Miseur B.: **SS 2.10**
Mittal R.: SS 3.3
Moelker A.: SS 11.10
Møller J.: SS 1.8
Monteleone I.: SS 12.3
Moro F.: SS 10.9
Morosi C.: SS 9.10
Morris E.: SS 1.2
Morton D.: SS 1.9
Muglia R.: SS 5.6
Mulders L.: SS 6.8
Mulé S.: SS 4.3, SS 10.2
Müller C.: SS 10.1
Multicenter MRI rectal study group: SS 11.1, SS 11.2, SS 11.3
Muthoo C.: **SS 3.7, SS 3.8**

### N

Nacci I.: **SS 1.7**
Nachit M.: **SS 10.5**
Nakuz T.: SS 6.5
Narayan S.: SS 8.6
Nault J.-C.: SS 10.2
Nederveen A.J.: SS 7.7, SS 7.8
Nelissen J.L.: SS 7.7, SS 7.8
Nellore S.: SS 4.10
Ng D.C.: SS 5.3
Nguyen H.: SS 5.9
Nics L.: SS 6.5
Nilsson E.: SS 8.3
Nougaret S.: SS 11.1, SS 11.2, SS 11.3
Nuñez L.: SS 5.3
Nuzzo A.: SS 9.4, SS 9.6

### O

O'Malley R.B.: SS 11.5
Obmann V.: SS 10.7
Olivieri A.: SS 2.7
Ordás I.: SS 6.2
Orvieto M.: **SS 12.7**
Osuji V.: SS 7.5
Ozdemir D.: SS 2.5
Özen Atalay H.: **SS 6.7**

### P

Paisant A.: SS 4.1, **SS 4.4**, SS 4.6
Palatresi D.: SS 2.1, **SS 9.9**
Panes J.: SS 6.2
Panis Y.: SS 9.4
Pansini M.: **SS 5.3**, **SS 7.6**, SS 8.4
Paradis V.: SS 10.5
Park E.J.: SS 3.5
Park M.S.: **SS 12.6**
Parry T.: SS 6.1
Patel D.: SS 12.1
Patil S.: SS 11.5
Paulatto L.: SS 5.4, SS 9.4, SS 9.6
Pedersen M.R.: SS 1.8
Perez A.A.: SS 1.4
Peters F.: SS 3.10, SS 11.6
Pickels E.: SS 5.3
Pickhardt P.J.: **SS 1.4**
Pickhardt S.G.: SS 1.4
Pilisi R.: SS 7.2
Pinter M.: SS 10.1
Platt J.: **SS 1.6**
Plumb A.: SS 6.1
Podesta C.: SS 10.3
Poetter-Lang S.: SS 2.3, **SS 8.2**, SS 10.6, SS 12.5
Polici M.: SS 1.7, SS 10.10
Pomej K.: SS 10.1
Porrello G.: SS 8.7
Porta M.: SS 12.9
Pradella S.: SS 9.9
Previtali C.: SS 7.4
Priyanthi T.: SS 5.3
Prokop M.: SS 2.4, SS 2.5
Pu H.: SSD 1.4
Pucciarelli F.: **SS 10.10**
Pugliese D.: SS 12.4
Purysko A.S.: SS 11.5

### Q

Quaia E.: SS 12.7
Quirke P.: SS 1.2, SS 1.9, SS 3.1, SS 3.7

Scientific Sessions

Authors' Index

### R

Rafaelsen S.R.: **SS 1.8**
Ragusi M.: SS 1.3, SS 7.3, SS 10.4
Rahr H.: SS 1.8
Rajagopal R.: SS 7.9
Raman S.: SSD 1.1
Ramkhelawon R.: SS 7.6
Rasul S.: SS 6.5
Raynaud L.: SS 5.4, SS 9.4, SS 9.6
Rebours V.: SS 7.1, SS 7.4
Reiberger T.: SS 10.6
Reinisch W.: SS 6.5
Reizine E.: **SS 4.3**
Ricke J.: SS 8.10
Rimola J.: **SS 6.2**
Ringe K.I.: SS 8.3
Roberti S.: SS 11.10
Rodriguez S.: SS 6.2
Ronot M.: **SS 4.1**, SS 4.5, SS 5.4, SS 5.10, **SS 7.1**, SS 7.4, SS 8.7, SS 9.4, SS 9.6, SS 10.2, SS 10.8
Rorsman F.: SS 8.3
Roskams T.: SS 2.10
Rossi V.: SS 2.8
Rossington H.: SS 3.1, SS 3.7
Roth A.A.: SS 7.5
Roux M.: SS 4.4, SS 4.6
Rübenthaler J.: SS 8.10
Runge J.: SS 6.4, SS 7.7, SS 7.8

### S

Sabnis S.: SS 4.10
Sack I.: SS 7.7
Saeed O.: SS 7.5
Salahshour F.: **SS 9.1**
Sansotta L.: SS 12.3
Sartoris R.: SS 4.5, **SS 7.4**, SS 10.2
Sathyakumar K.: SS 3.3
Sauvanet A.: SS 7.4
Scarsbrook A.: SS 10.3
Scatton O.: SS 10.2
Schaik C.: SS 2.5
Scharitzer M.: **SS 6.5**, SS 10.1
Scheiner B.: SS 10.1
Schinas G.: SS 6.9
Schindl M.: SS 2.3
Schmid-Tannwald C.: SS 8.10
Schrauben E.M.: SS 7.7, SS 7.8
Schurink N.W.: SS 3.2, SS 11.1, SS 11.2, SS 11.3
Schwartz M.: SS 4.2
Seligmann J.: SS 1.6, SS 1.9
Seror O.: SS 10.2
Seymour M.: SS 1.9
Shah N.: SS 12.1
Shaker E.: SS 9.1
Shamsi K.: **SS 11.7**
Shemesh N.: SS 2.2
Shen J.: SSD 1.3
Shen L.: SSD 1.1, SSD 1.2, SS 4.7, SS 4.8
Shia J.: SS 1.5
Shojaeshafiei F.: SS 9.1
Sibert A.: SS 5.4
Siddiqui S.: SS 4.10
Sigel K.: SS 4.2
Simões R.V.: SS 2.2
Simonini R.: SS 12.9
Singh N.: SS 8.6
Singh P.: SS 8.6
Sinkus R.: SS 7.8
Sironi S.: SS 5.6, SS 5.7, SS 9.5, SS 12.9
Sjöström M.: SS 1.8
Soydan L.: SS 2.6, SS 6.7
Soykan E.: SS 11.9
Staal F.: SS 11.10
Starmans M.: SS 11.10
Stoker J.: SS 6.4, SS 6.6, SS 6.8, SS 7.7, SS 7.8, SS 11.8
Straube U.: SS 7.2
Struchkov V.: SS 8.9
Summers R.M.: SS 1.4
Sureka B.: SS 7.9
Suriano R.: SS 7.6
Suter Y.: SS 10.7
Sutter O.: SS 4.4, SS 4.6
Swallow C.: SS 9.10
Swijnenburg R.J.: SS 11.8
Sznitman R.: SS 10.7

### T

Taghavi M.: SS 11.10
Talei Franzesi C.: SS 1.3, SS 7.3, SS 10.4
Tamandl D.: SS 10.1
Tan I.B.: SS 5.3
Tanis P.J.: SS 3.2
Taouli B.: SS 4.2, SS 12.2
Tarique U.: SS 9.10
Taylor J.: SS 1.2, SS 3.1
Taylor S.: SS 6.1
Telford A.: SS 8.4
Teodorescu-Arghezi E.: SS 3.9
Terashima K.: SSD 1.1
Thanabalasingham G.: SS 7.6
Thomaides-Brears H.: SS 7.6
Thompson H.: SS 1.5
Thrower A.: SS 12.1
Tielbeek J.: SS 6.8
Tikhonova V.: SS 8.9
Ting M.: SS 3.8
Tirkes T.: **SS 7.5**
Tissier R.: SS 11.1, SS 11.2
Tiwari S.: SS 7.9
Todesco M.: SS 2.7, SS 2.8, SS 2.9, SS 5.2, SS 10.9
Tolan D.: **SS 1.2**, SS 1.6, **SS 1.9**, **SS 3.1**, SS 3.7
Tomaiuolo L.: SS 2.7, SS 2.8, SS 2.9, SS 5.2, **SS 10.9**
Topal B.: SS 2.10
Trauner M.: SS 10.1
Trillaud H.: SS 4.6
Trivedi P.J.: SS 8.4
Troelstra M.: SS 7.8
Tse G.: SSD 1.3
Tse J.: **SSD 1.1**, **SSD 1.2**, **SSD 1.3**, **SS 4.7**, **SS 4.8**

### U

Ubaldi N.: SS 12.4
Unger L.W.: SS 6.5
Urso D.: SS 12.7
Uzer E.: SS 7.10

### V

Vagn-Hansen C.A.: SS 1.8
Valanzuolo D.: SS 12.4
Valle C.: SS 9.5
Van Beers B.: SS 10.5
Van Cutsem E.: SS 2.10
van den Hurk E.: SS 11.6
van den Hurk M.: SS 2.5
Van der Kolk M.: SS 2.4
van der Reijd D.: SS 3.10, SS 11.4, **SS 11.9**, **SS 11.10**
van der Sommen F.: SS 2.5
van der Woude R.: SS 7.7
van Geenen E.: SS 2.5
van Griethuysen J.: SS 11.1, SS 11.2, SS 11.3
van Harten L.D.: **SS 6.6**
Van Laarhoven C.J.: SS 2.4
van Laarhoven H.W.: SS 7.7
van Laarhoven K.: SS 2.5
van Leerdam M.: SS 3.10, SS 11.4
van Rosmalen B.I.: **SS 2.4**
van Schelt A.-S.: SS 6.4, SS 7.7, **SS 7.8**
van Triest B.: SS 3.10, SS 11.4, SS 11.6
Vandecaveye V.: SS 2.10
Varshney V.: SS 7.9
Veniero J.C.: SS 11.5
Verhoef C.: SS 11.8, SS 11.10

Vernuccio F.: SS 12.7
Verpalen I.M.: SS 11.8
Verrengia F.: SS 2.7
Vietti Violi N.: **SS 4.2**, **SS 12.2**, SS 12.8
Viktil E.: **SS 3.4**
Vilgrain V.: SS 4.1, SS 4.4, SS 4.5, SS 4.6, SS 5.4, SS 5.10, SS 7.4, SS 8.7, SS 9.4, SS 9.6, SS 10.2, SS 10.5, SS 10.8
Villard N.: SS 12.8
Vinayagam R.: SS 1.1
Volterrani L.: SS 12.3
Voncken F.E.: SS 11.6
Vullierme M.-P.: SS 8.7

### W

Wagner M.: SS 4.2, SS 10.2
Wai Meng Tai D.: SS 5.3
Wasnik A.P.: SS 11.5
Wassenaar N.P.: SS 6.4, **SS 7.7**, SS 7.8
Weber M.: SS 6.5
West N.: SS 1.6, SS 1.9
Widmar M.: **SS 1.5**
Woolgar J.: SS 7.6

### Y

Yadav T.: SS 7.9
Yagnik R.: SS 8.6
Yan S.X.: SS 5.3
Yoon J.-H.: SS 3.5
Yoon L.: SS 4.7, SS 4.8
Young L.: SS 8.4
Yousif A.O.: **SS 1.1, SS 3.9**
Yuan Y.: SSD 1.4
Yuval J.B.: SS 1.5, **SS 11.5**

### Z

Zampakis P.: SS 6.9
Zamyatina K.: SS 8.9
Zbinden L.: **SS 10.7**
Zea R.: SS 1.4
Zerunian M.: SS 1.7, SS 10.10
Zheng J.: SS 1.5
Zurlo M.T.: SS 5.8
Zyromski N.: SS 7.5
